# The impact of p53 mutation on tumor immune evasion: mechanistic insights and clinical implications

**DOI:** 10.3389/fimmu.2026.1753215

**Published:** 2026-02-06

**Authors:** Luo Liang, Weidong Wang

**Affiliations:** 1 Department of Radiation Oncology, Sichuan Clinical Research Center for Cancer, Sichuan Cancer Hospital and Institute, Sichuan Cancer Center, School of Medicine, University of Electronic Science and Technology of China, Chengdu, China; 2 Department of Head and Neck Radiation Oncology, Radiation Oncology Key Laboratory of Sichuan Province, Sichuan Clinical Research Center for Cancer, Sichuan Cancer Hospital and Institute, Sichuan Cancer Center, University of Electronic Science and Technology of China, Chengdu, China

**Keywords:** epigenetic regulation, immune evasion, immunotherapy, metabolic reprogramming, p53 mutation, tumor microenvironment

## Abstract

Mutant p53(Mtp53) not only loses its canonical tumor-suppressive functions but also acquires oncogenic gain-of-function properties, positioning it as a central orchestrator in reshaping the tumor immune microenvironment. This review systematically delineates how Mtp53 actively establishes and sustains an immunosuppressive niche through multiple interconnected mechanisms, including chronic inflammation, immune cell dysfunction, reprogramming of cancer-associated fibroblasts, metabolic dysregulation, epigenetic hijacking, and potentially aberrant liquid–liquid phase separation, thereby promoting immune evasion and therapeutic resistance. We integrate current evidence to propose a conceptual “metabolism–epigenetics–immunity” axis: Mtp53-driven metabolic reprogramming—such as accumulation of lactate or α-ketoglutarate—can modulate chromatin modifications and immune gene expression. Notably, the full *in vivo* causal chain of this axis remains unestablished; existing support derives primarily from stepwise experimental data and strong correlations. The immunological impact of Mtp53 is highly context-dependent, shaped by co-mutations and tissue origin. In TP53/KRAS co-mutant non-small cell lung cancer (NSCLC), Mtp53 enhances tumor immunogenicity and improves response to immune checkpoint inhibitors (ICIs); conversely, in immunologically “cold” tumors—such as triple-negative breast cancer, pancreatic ductal adenocarcinoma, and colorectal cancer—it promotes T-cell exhaustion or myeloid suppression, reflecting marked cancer-type heterogeneity. Therapeutic approaches include Mtp53 reactivators (e.g., APR-246, PC14586), degraders, synthetic lethal strategies, and neoantigen vaccines. Although APR-246 showed efficacy in a phase II trial (NCT03072043), it failed to improve survival in phase III (NCT03745716) due to lack of TP53 mutation stratification. Its combination with pembrolizumab (NCT04383938) demonstrated acceptable safety (immune-related adverse events in ∼12%) but limited efficacy, underscoring the need for biomarker-guided, precision-based combinations. Thus, a multidimensional biomarker platform is urgently needed—one integrating TP53 mutation subtypes (e.g., R175H *vs*. nonsense mutations), dynamic ctDNA monitoring (VAF ≥ 0.01%), tumor immune microenvironment (TIME) features (e.g., TILs, MDSCs), and spatial multi-omics—to enable precise molecular stratification and personalized intervention in Mtp53-driven cancers.

## 1 Introduction

The tumor suppressor protein p53 was first identified in 1979 in cells transformed by simian virus 40 (SV40) and remains one of the most extensively studied proteins in cancer biology (1). Classically, p53 orchestrates cellular responses to genotoxic and other stresses by inducing cell cycle arrest, apoptosis, DNA repair, and senescence. Beyond these canonical tumor suppressive functions, p53 also acts as a central regulator of immunity, modulating inflammatory responses, immune cell proliferation and differentiation, and the immunosuppressive landscape of the tumor microenvironment (TME) (2).

The composition and functional state of the tumor immune microenvironment (TIME) are critical determinants of response to immunotherapy. Tumors are increasingly classified as “hot” (inflamed) or “cold” (immune desert or immune excluded) based on the abundance and activity of tumor-infiltrating lymphocytes (TILs), particularly CD8^+^ T cells (3). Hot tumors exhibit robust T cell infiltration, efficient antigen presentation, and active interferon signaling, features generally associated with favorable responses to immune checkpoint blockade (ICB). In contrast, cold tumors are characterized by defective antigen presentation, upregulation of immune checkpoint molecules such as PD-L1, and enrichment of immunosuppressive cell populations, including regulatory T cells (Tregs), tumor-associated macrophages (TAMs), and myeloid-derived suppressor cells (MDSCs) (4, 5). Collectively, these features confer intrinsic resistance to immunotherapy. Importantly, this immunosuppressive phenotype is often actively driven by oncogenic signaling pathways that remodel the TIME to facilitate immune evasion and therapeutic resistance (6).

Wild-type p53 (Wtp53) suppresses tumorigenesis and enhances antitumor immunity through mechanisms such as immunogenic cell death and antigen presentation. In contrast, Mtp53 frequently acquires gain of function (GOF) properties that actively reprogram the TIME toward an immunosuppressive and therapy-resistant state (7). These GOF activities include metabolic rewiring, epigenetic dysregulation, aberrant exosome secretion, dysregulated cytokine and chemokine signaling, suppression of cytotoxic lymphocyte function, and recruitment of immunosuppressive myeloid and regulatory T cells. Paradoxically, certain Mtp53 variants can also serve as immunogenic neoantigens capable of eliciting antitumor immune responses, highlighting the dual and context-dependent role of p53 dysfunction in immune regulation (8).

Accumulating evidence indicates that p53 inactivation is not merely a passive loss of tumor suppression but an active driver of immune escape. Loss of p53 function leads to derepression of endogenous retroviruses (ERVs) and transposable elements, triggering a viral mimicry state in which self-nucleic acids are recognized as foreign, resulting in chronic activation of type I interferon pathways. However, rather than promoting immunity, this persistent signaling often fosters immune tolerance and T cell exhaustion (9). Conversely, pharmacologic restoration of Mtp53 function using compounds such as APR-246, which covalently modifies Mtp53 to restore its wild type conformation, has been shown to reverse immunosuppression, enhance T cell infiltration, and induce tumor regression (10). These seemingly contradictory observations underscore p53 as a pivotal nexus linking genomic integrity to immune surveillance. Its wild type and mutant forms dictate divergent evolutionary trajectories of the TIME.

Building on these insights, we propose a conceptual framework—the “metabolism–epigenetics–immunity axis”—to systematically explain how Mtp53 drives remodeling of the TIME. In this model, Mtp53 initiates metabolic reprogramming, leading to aberrant accumulation or depletion of key metabolites such as lactate, α-ketoglutarate, and acetyl-CoA. These metabolites serve as substrates or cofactors for epigenetic-modifying enzymes, thereby directly reshaping chromatin architecture and transcriptional programs. While individual components of this axis have been supported by separate studies, the complete *in vivo* causal cascade—from Mtp53-driven metabolic alterations through epigenetic reprogramming to functional immune evasion—has not yet been fully validated in genetically engineered animal models. Nevertheless, the crosstalk between metabolism and epigenetics is thought to stabilize an immunosuppressive TIME, thereby promoting immune escape and therapy resistance. This mechanistic cascade provides a theoretical foundation for understanding the GOF properties of Mtp53 and reveals multiple potential nodes for therapeutic intervention.

Liquid liquid phase separation (LLPS) is a biophysical process that drives the formation of membraneless organelles such as nucleoli and stress granules. It has emerged as a key mechanism in pathological protein aggregation in neurodegenerative diseases and cancer (11). Recent studies suggest that Mtp53 may exploit aberrant LLPS to acquire oncogenic activity. However, how Mtp53 harnesses LLPS to specifically rewire immune signaling pathways and promote immune escape remains poorly understood and represents a critical frontier for future investigation.

Moreover, the functional consequences of Mtp53 exhibit marked heterogeneity across tumor types and genomic contexts. For example, in TP53/KRAS co-mutant non-small cell lung cancer (NSCLC), Mtp53 is frequently associated with a high tumor mutational burden and enhanced immunogenicity, and paradoxically correlates with improved clinical responses to immune checkpoint inhibitors (ICIs) (12). In contrast, in immunologically “cold” tumors—such as pancreatic ductal adenocarcinoma (PDAC), colorectal cancer (CRC), and triple-negative breast cancer (TNBC)—Mtp53 drives T-cell exclusion or exhaustion through cancer-type-specific mechanisms, including CXCL1-mediated expansion of polymorphonuclear myeloid-derived suppressor cells (PMN-MDSCs), exosome-mediated myeloid polarization, or loss of miR-34a, thereby promoting more aggressive disease progression (13, 14).

This profound context-dependent functional plasticity limits the utility of TP53 mutation status as a universal biomarker for ICI response.

In this review, we integrate recent advances in understanding the molecular mechanisms by which Mtp53 establishes an immunosuppressive network, with a focus on metabolic reprogramming, epigenetic alterations, and immune signaling dysregulation. We not only use triple-negative breast cancer (TNBC)—a subtype characterized by high TP53 mutation frequency and limited therapeutic options—as a paradigm, but also systematically compare the divergent roles of Mtp53 across multiple tumor types, including those with high tumor mutational burden and those exhibiting immune-excluded phenotypes. Furthermore, we critically evaluate ongoing clinical trials, particularly those testing APR-246 in combination with azacitidine (NCT03072043/NCT03745716) or pembrolizumab (NCT04383938). The phase III failure underscores the necessity of stratifying patients by TP53 mutation subtype, while early combination data indicate manageable safety (with immune-related adverse events in ∼12%) yet modest efficacy—highlighting the need for more precisely designed therapeutic strategies. By bridging mechanistic insights with clinical translation, this review aims to provide a conceptual foundation for precision targeting of Mtp53 driven immune evasion and to advance the development of more effective cancer therapies.

## 2 Functional alterations of Mtp53

As a transcriptional regulator, TP53 is expressed at low basal levels in normal, unstressed cells, and its protein stability is primarily controlled by MDM2-mediated ubiquitination and subsequent proteasomal degradation. Upon exposure to cellular stresses such as DNA damage or oxidative stress, p53 rapidly accumulates and becomes activated, triggering cell cycle arrest. In addition, p53-dependent apoptosis can be initiated through the extrinsic pathway involving death receptors (e.g., Fas, DR5) or the intrinsic mitochondrial pathway, which is predominantly governed by the Bcl-2 protein family (e.g., PUMA, NOXA, BAX) (15).

Beyond these canonical tumor-suppressive functions, p53 also plays a critical role in maintaining tissue and developmental homeostasis by regulating cellular metabolism, ferroptosis, and antitumor immunity.

**Figure 1:**
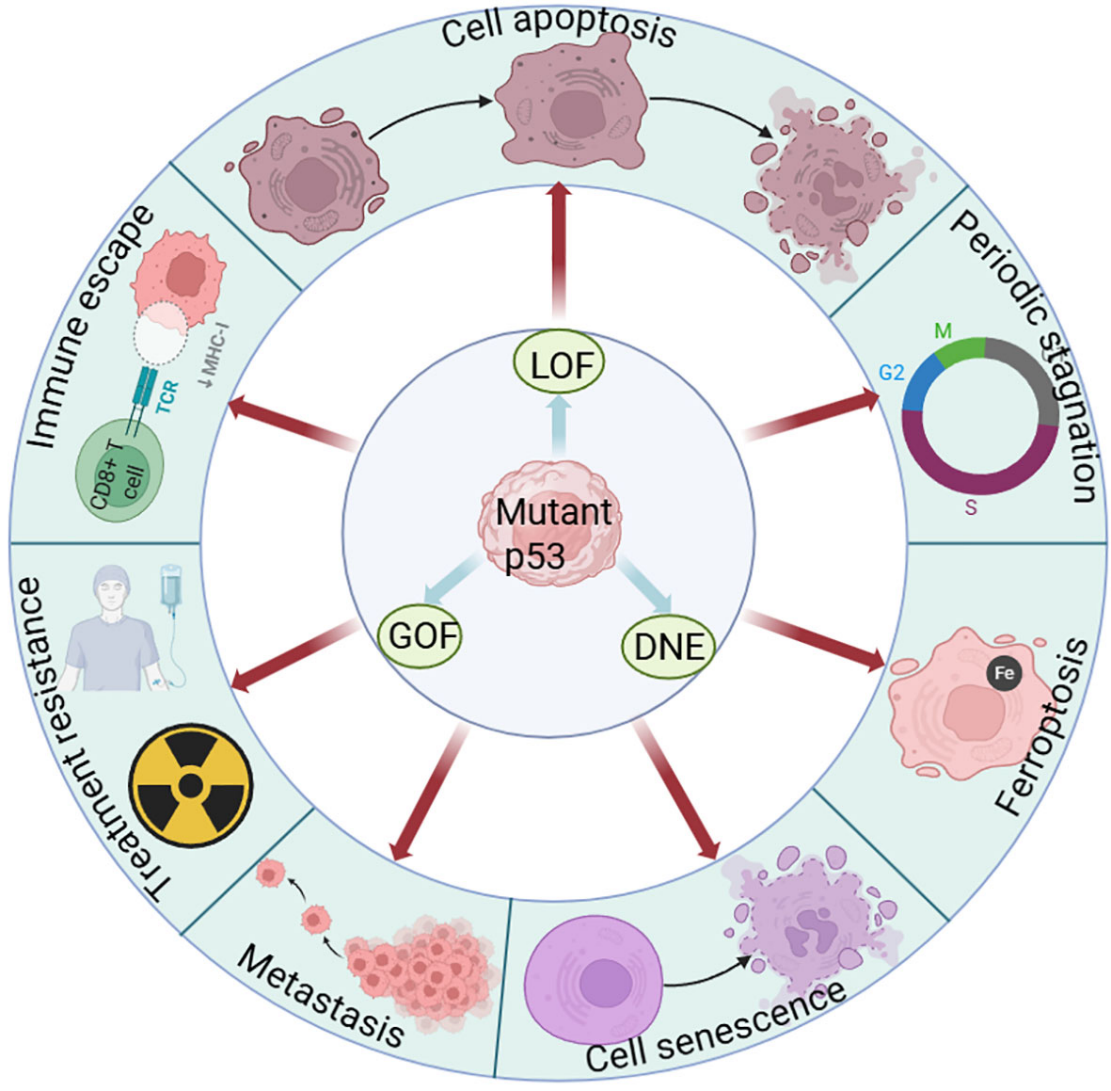
TP53 mutation subtypes drive tumor progression. TP53 mutations are classified into three types: loss-of-function (LOF), dominant-negative effect (DNE), and gain-of-function (GOF), which drive tumor metastasis, immune evasion, and therapy resistance by inhibiting key mechanisms such as cell cycle arrest, apoptosis, and cellular senescence.

TP53 mutations can be broadly categorized into three functional classes: loss of function (LOF), dominant-negative effect (DNE), and gain of function (GOF). LOF mutations disrupt critical residues within the DNA-binding domain—most commonly positively charged arginine residues—or impair structural motifs essential for proper protein folding, thereby abolishing p53’s ability to transactivate genes involved in cell cycle control, apoptosis, senescence, and immune surveillance (16). DNE occurs when Mtp53 forms transcriptionally inactive heterotetramers with Wtp53, thereby compromising the transcriptional activity of any remaining functional Wtp53 allele (17) (**Figure 1**). Notably, the phenotypic consequences of TP53 mutations typically arise from a combination of these mechanisms, with their relative contributions dynamically shaped by cellular context, tissue microenvironment, and disease stage. This functional complexity is clearly demonstrated in genetically engineered mouse models: mice expressing the p53R172H GOF mutant develop tumors earlier, exhibit significantly higher metastatic burden, and have markedly shorter median survival compared with mice harboring complete p53 loss (LOF) (18). Collectively, these findings indicate that different functional classes of Mtp53 exert distinct—and often more aggressive—effects on tumor progression and antitumor immunity.

Therefore, to optimize therapeutic efficacy, strategies targeting Mtp53 must be grounded in precise molecular characterization of the specific mutant variant.

## 3 Emerging mechanisms: phase separation and reprogramming of the tumor microenvironment

The tumor suppressor p53 is among the most frequently mutated genes in human cancer. Traditionally recognized for its roles in cell cycle arrest and apoptosis, p53 is now appreciated as a central guardian of cellular homeostasis. However, Mtp53 often acquires gain-of-function (GOF) activities that actively reprogram the TIME. This section focuses on two emerging paradigms: LLPS as a novel mechanistic framework, and the intricate, multifaceted reprogramming of immune and stromal components.

### 3.1 Phase separation: a new paradigm for Mtp53 in tumorigenesis and immune regulation

Liquid–liquid phase separation (LLPS) is a biophysical process driven by multivalent molecular interactions that mediates the formation of membraneless organelles—such as nucleoli and stress granules—and plays a pivotal role in subcellular compartmentalization and gene expression regulation. Dysregulation of LLPS can cause biomolecular condensates to transition from dynamic liquid states into pathological gel-like or solid aggregates, a phenomenon implicated in neurodegenerative diseases and cancer (19, 24). Recent *in vitro* studies suggest that certain TP53 hotspot mutants exhibit enhanced liquid–liquid phase separation (LLPS) propensity compared to wild-type p53. Petronilho et al. observed in overexpression systems that Mtp53 (e.g., R175H) forms more stable and persistent nuclear condensates (20). Yu et al. further proposed, using molecular dynamics simulations, that oncogenic mutations (e.g., R337H) may accelerate LLPS by strengthening electrostatic and hydrophobic interactions within the C-terminal domain (23). Together, these findings support a hypothesis: Mtp53 may transition from a passive “client” within physiological phase-separated complexes to an active “scaffold” that drives aberrant condensate formation (19).

However, it must be emphasized that no study to date has demonstrated a causal role for Mtp53 LLPS in tumorigenesis or immune evasion under physiologically relevant conditions—i.e., at endogenous expression levels or *in vivo* tumor models—using either genetic (e.g., IDR mutations) or pharmacological disruption of LLPS. All current evidence relies on purified proteins, overexpression cell lines, or computational modeling. Consequently, whether LLPS constitutes a necessary or sufficient mechanism for Mtp53 gain-of-function (GOF) remains purely theoretical.

In principle, Mtp53-containing nuclear condensates could serve as platforms for transcriptional reprogramming. Super-enhancers—phase-separated hubs enriched in BRD4 and RNA Pol II—drive oncogenic and immunosuppressive gene networks. Mahat et al. reported that in PDAC, Mtp53-R172H is recruited by NF-κB to the CXCL1 enhancer, where it co-activates the CXCL1–CXCR2 axis to expand polymorphonuclear myeloid-derived suppressor cells (PMN-MDSCs) and tumor-associated neutrophils (TANs), thereby suppressing CD8^+^T-cell function (21). Although this study highlights a critical immunosuppressive role of Mtp53, it did not assess condensate formation, perform fluorescence recovery after photobleaching (FRAP) to confirm liquid-like properties, or test LLPS dependence via mutagenesis of Mtp53’s intrinsically disordered region (IDR). Thus, whether this immunosuppressive phenotype is mediated by LLPS remains undetermined.

**Figure 2:**
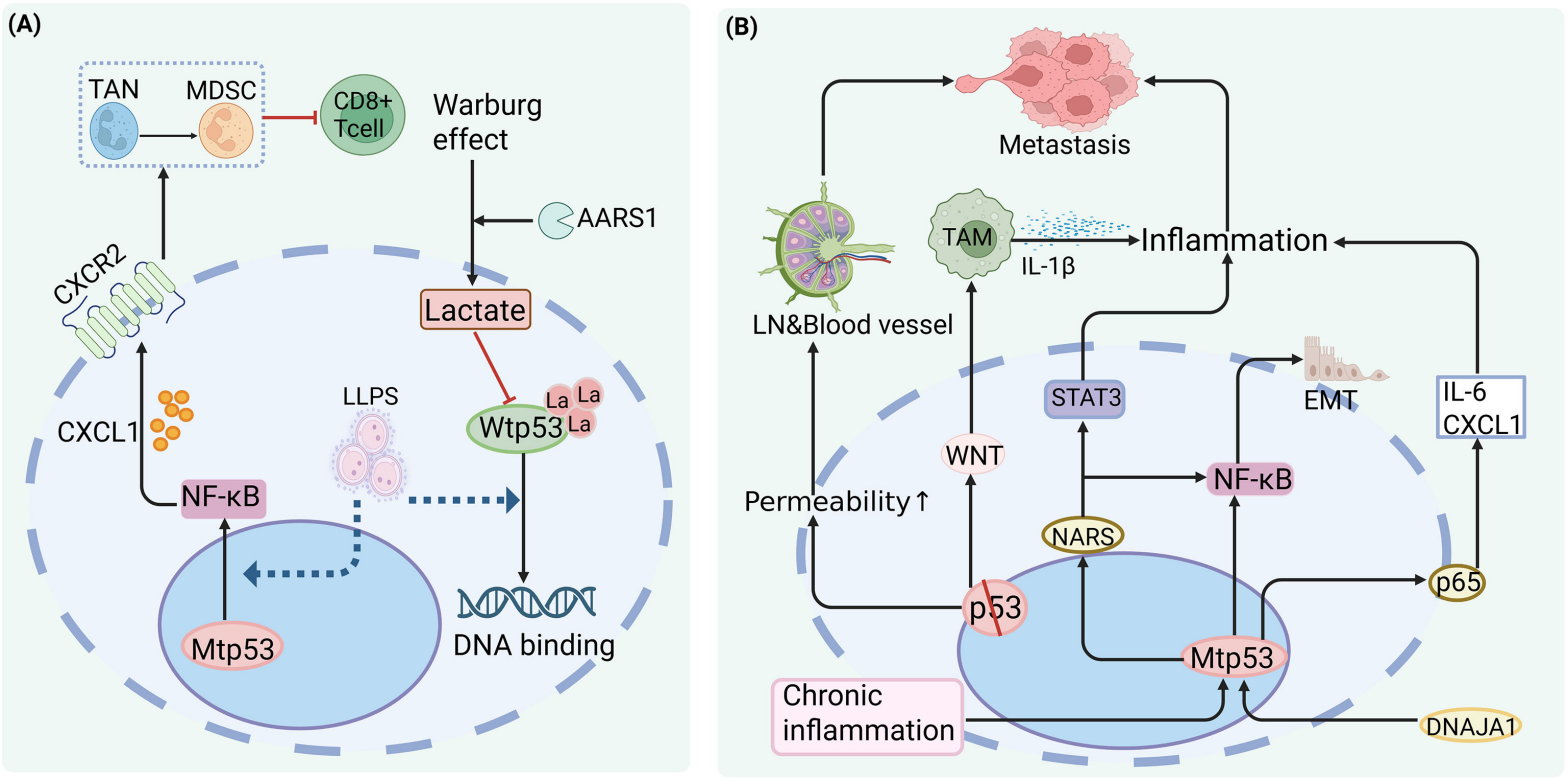
Mtp53 drives tumor immune evasion via the NF-κB signaling pathway; liquid–liquid phase separation (LLPS) may be involved in its regulation, but direct experimental evidence is currently lacking. **(A)** ① Mtp53 (e.g., R172H) cooperates with the NF-κB p65 subunit at the CXCL1 enhancer to upregulate CXCL1 expression, promoting neutrophil differentiation into polymorphonuclear myeloid-derived suppressor cells (PMN-MDSCs) and tumor-associated neutrophils (TANs), which suppress CD8^+^ T cell function. ② Lactate accumulation in tumor cells induces lysine lactylation of wild-type p53 (Wtp53) at K120/K139, impairing its DNA-binding capacity. ③ LLPS may facilitate Mtp53-mediated transcriptional regulation, though this remains to be experimentally validated. **(B)** ① The chaperone DNAJA1 stabilizes Mtp53 and enhances its interaction with NF-κB p65, boosting transcription of pro-inflammatory cytokines such as IL-6 and CXCL1. ② Mtp53 cooperates with NF-κB to activate epithelial–mesenchymal transition (EMT)-related genes, promoting tumor cell migration. ③ In the context of NRAS mutation, Mtp53 co-activates both NF-κB and STAT3 pathways, accelerating tumor progression. ④ Complete p53 loss (rather than mutation) compromises intestinal epithelial barrier integrity, leading to microbial translocation, chronic inflammation, and a pro-metastatic microenvironment.

Notably, a potential tension exists between the LLPS model and metabolic signaling in the tumor microenvironment. Zong et al. found that lactate induces lysine lactylation of p53 at K120/K139 via AARS1, impairing its DNA-binding capacity (22). Given that the C-terminal domain of p53—rich in modifiable lysines—is implicated in LLPS, lactylation could theoretically alter its phase separation behavior, though this remains experimentally unverified (**Figure 2A**). Paradoxically, lactate itself is a potent immunosuppressive metabolite (e.g., inhibiting T-cell function and promoting Treg/M2 polarization; see Section 4.1.1). If lactate simultaneously suppresses Mtp53 LLPS yet enhances immune evasion, this implies that Mtp53-driven immunosuppression may operate primarily through LLPS-independent mechanisms—such as direct protein–protein interactions or transcriptional co-activation—rather than condensate formation. In other words, LLPS may not be the primary effector of lactate-mediated immune escape.

In summary, Mtp53 likely influences the tumor immune microenvironment through both LLPS-dependent pathways (e.g., forming aberrant condensates to concentrate transcription factors) and LLPS-independent pathways (e.g., acting as a chromatin-bound co-activator). Current data are insufficient to discern their relative contributions *in vivo*. Future studies urgently require genetically engineered tools—such as IDR point mutations—that specifically disrupt Mtp53 LLPS without perturbing its overall structure or protein interactions, coupled with functional assessment in immunocompetent mouse models to define the immunological impact of Mtp53 condensates.

### 3.2 Remodeling the tumor–immune landscape: cellular and molecular mechanisms

The role of Mtp53 in reshaping the tumor immune microenvironment represents a key mechanism of immune evasion. Beyond its cell-autonomous oncogenic effects, Mtp53 broadly modulates stromal and immune compartments to construct an immunosuppressive niche. This subsection details Mtp53’s multidimensional regulatory functions across three axes: inflammatory signaling, immune cell functionality, and cancer-associated fibroblasts (CAFs), elucidating the molecular mechanisms driving immune evasion.

#### 3.2.1 Tumor-associated inflammation and the NF-κB signaling pathway

Acute, controlled inflammation can activate innate and adaptive immunity, serving protective and tumor-suppressive roles. In contrast, chronic inflammation is a well-established driver of tumorigenesis, promoting genomic instability, angiogenesis, epigenetic dysregulation, and accelerated cell proliferation (25). Central to this process is the nuclear factor-kappa B (NF-κB) signaling pathway, which is constitutively activated in many cancers. Sustained NF-κB activity maintains a pro-tumorigenic inflammatory milieu, recruits immune cells, and releases inflammatory mediators that foster a permissive ecosystem for disease progression and therapeutic resistance.

Notably, Mtp53 not only loses tumor-suppressive capacity but also actively hijacks the NF-κB pathway through GOF mechanisms to amplify pro-tumorigenic inflammation. In breast cancer, the chaperone DNAJA1 stabilizes Mtp53 and facilitates its interaction with the NF-κB subunit p65, enhancing transcription of target genes such as IL-6 and CXCL1, thereby driving tumor proliferation and metastasis (26). In head and neck squamous cell carcinoma, p53 mutation cooperates with NF-κB to promote epithelial–mesenchymal transition (EMT) and invasive phenotypes—an effect reversible upon NF-κB inhibition (27). In hematologic malignancies, Mtp53 synergizes with oncogenic NRAS mutations to sustain activation of both NF-κB and signal transducer and activator of transcription 3 (STAT3) pathways, accelerating the onset and progression of acute myeloid leukemia (AML) (28).

Loss of p53 function—whether through genetic deletion or point mutation—can disrupt tissue homeostasis and trigger NF-κB–dependent chronic inflammation. For example, intestinal epithelium-specific p53 deficiency compromises barrier integrity, leading to local NF-κB activation and a pro-inflammatory microenvironment that promotes tumor invasion and lymph node metastasis (29). Similarly, p53 loss in mammary epithelium induces WNT ligand secretion, which activates TAMs to produce IL-1β, triggering systemic inflammation and facilitating distant metastasis (30).

As previously noted, Mtp53 directly interacts with NF-κB and co-occupies the CXCL1 enhancer region, synergistically driving expression of the CXCL1–CXCR2 axis to promote infiltration of immunosuppressive neutrophils and impair anti-tumor immunity (21). Importantly, this relationship is bidirectional: the chronic inflammatory milieu itself can reciprocally shape the p53 mutational landscape. In secondary AML (sAML), persistent inflammatory signals—such as interferon-gamma (IFNγ)—suppress wild-type p53 function in hematopoietic stem and progenitor cells, conferring a strong selective advantage to TP53-mutant clones and accelerating their clonal expansion and evolution (31) (**Figure 2B**).

Collectively, Mtp53 and NF-κB engage in a self-reinforcing positive feedback loop that constitutes a core engine of cancer-associated inflammation. This axis not only deepens our understanding of immune evasion but also offers novel therapeutic opportunities for targeting the immunosuppressive tumor microenvironment in cancer immunotherapy.

#### 3.2.2 Immune cell dysfunction

##### 3.2.2.1 T cells

The functional dichotomy between effector T cells and Tregs underscores the centrality of immunometabolism. Activated effector T cells typically rely on robust aerobic glycolysis to meet their energetic and biosynthetic demands. However, Mtp53 in tumor cells impairs T cell metabolic fitness and cytotoxic capacity by suppressing key glycolytic rate-limiting enzymes such as pyruvate kinase M2 (PKM2). This effect is particularly pronounced in glucose-deprived microenvironments and may even accelerate T cell apoptosis (32). These findings suggest that therapeutic reprogramming of T cell metabolism could represent a viable strategy to counteract Mtp53-mediated immunosuppression.

The immunomodulatory influence of Mtp53 extends beyond cell-autonomous mechanisms. It also indirectly shapes an immunosuppressive niche by remodeling the tumor secretome. For example, p53 loss promotes the secretion of multiple chemokines—including ligands for CXCR3/CCR2 and macrophage colony-stimulating factor (M-CSF)—which recruit and “educate” CD11b^+^ myeloid cells to produce abundant IL-1 and IL-6. This not only suppresses CD4^+^/CD8^+^ T cell function but also unexpectedly drives Th17 differentiation, revealing a dual role for Mtp53-driven inflammation in immune evasion (33).

Recent studies have proposed that Mtp53 may influence T-cell metabolism through non–cell-autonomous mechanisms. Dong et al. reported that in an *in vitro* co-culture system, tumor-derived Mtp53 can be actively secreted and subsequently internalized by CD4^+^ T cells. Immunofluorescence and subcellular fractionation experiments revealed its partial mitochondrial localization, accompanied by downregulation of hexokinase-I (HK-I) and PFKP, leading to bioenergetic exhaustion in T cells (32).

Although this study provides preliminary evidence for the “secretion–uptake” phenomenon of Mtp53, the prevalence of this process in the *in vivo* tumor microenvironment and its functional causality—such as validation using secretion-deficient Mtp53 mutants—remain to be rigorously established.

A similar phenomenon occurs in hematologic malignancies, albeit through more complex mechanisms. In AML, Mtp53 not only causes intrinsic defects in antigen presentation—such as downregulation of major histocompatibility complex (MHC) class I and II—but also systemically induces high expression of exhaustion markers (e.g., PD-1, TIM-3) on CD8⁺ T cells and significantly impairs NK cell degranulation. Concurrently, the Mtp53 AML microenvironment exhibits marked expansion of Tregs and MDSCs, reinforcing a broad immunosuppressive network. Notably, treatment with p53 conformational restorers such as eprenetapopt partially reverses these phenotypes, restoring T and NK cell functionality and highlighting the immunotherapeutic potential of p53-targeted reactivation (34). Furthermore, Mtp53 AML cells release exosomes enriched in heat shock protein 70 (HSP70) and immunosuppressive microRNAs (e.g., miR-155, miR-21), which are taken up by T cells, suppress their proliferation and IFN-γ production, and upregulate PD-1 and cytotoxic T-lymphocyte-associated protein 4 (CTLA-4), ultimately driving T cell exhaustion and immune escape (35). However, validation of this mechanism in patient-derived samples and the direct targets of the miRNA require further in-depth investigation.

**Figure 3:**
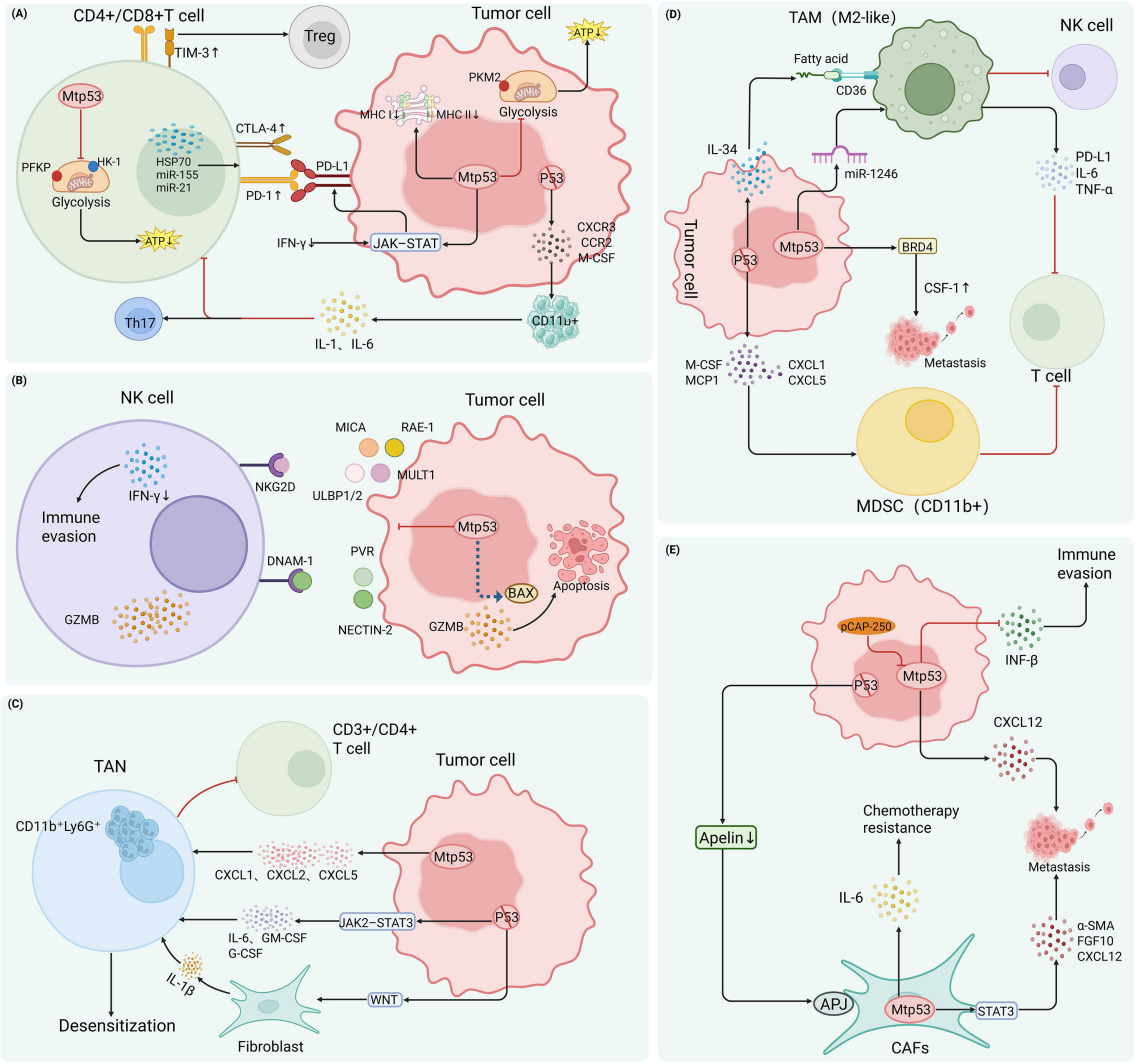
Mtp53 mediates immune evasion by modulating T cells, NK cells, tumor-associated neutrophils (TANs), myeloid-derived suppressor cells (MDSCs), tumor-associated macrophages (TAMs), and cancer-associated fibroblasts (CAFs). **(A)** Mtp53 suppresses T cell function through multiple mechanisms: ① In tumor cells, Mtp53 represses the glycolytic rate-limiting enzyme PKM2, impairing metabolic fitness and cytotoxicity of effector T cells. ② p53 loss promotes secretion of CXCR3/CCR2 ligands and M-CSF, recruiting CD11b^+^ myeloid cells that produce IL-1/IL-6, thereby suppressing CD4^+^/CD8^+^ T cells and driving Th17 differentiation. ③ Tumor-derived Mtp53 is taken up by CD4^+^ T cells, downregulating HK-I and PFKP, leading to glycolytic impairment and bioenergetic failure. ④ Mtp53 induces downregulation of MHC class I on tumor cells and defects in antigen presentation, while upregulating exhaustion markers (e.g., PD-1, TIM-3) on CD8^+^ T cells and promoting Treg expansion. ⑤ Mtp53 drives release of exosomes containing HSP70 and miR-155/21; upon internalization by T cells, these suppress proliferation and IFN-γ production while upregulating PD-1 and CTLA-4. *Notably, under high tumor mutational burden, Mtp53 can paradoxically enhance T cell infiltration and IFN-γ secretion, which in turn activates JAK–STAT signaling to induce PD-L1 expression, establishing adaptive immune resistance.***(B)** Mtp53 impairs NK cell-mediated immune surveillance: ① Mtp53 loses the ability to transcriptionally activate NKG2D ligands, resulting in marked downregulation of ULBP1 and ULBP2; it also suppresses MULT1 (murine ULBP homolog) and RAE-1 family ligands, reducing NK cell infiltration and IFN-γ production, enabling immune escape *in vivo*. ② Mtp53 fails to directly drive transcription of PVR (CD155) and Nectin-2 (CD112), leading to reduced ligand expression and weakened NK cell co-stimulatory signaling. ③ Due to loss of wild-type p53’s transcription-independent activation of BAX, Mtp53 compromises granzyme B–induced mitochondrial apoptosis, enhancing tumor cell resistance to NK killing. **(C)** Mtp53 drives neutrophil-mediated immunosuppression via multiple pathways: ① Mtp53 upregulates CXCL1/CXCL2/CXCL5, continuously recruiting CD11b^+^Ly6G^+^ neutrophils and reducing the CD3^+^/CD4^+^ T cell ratio. ② In the context of p53 loss, tumor-secreted WNT ligands induce stromal IL-1β release, triggering systemic inflammation and recruitment of activated neutrophils. ③ p53 loss activates the JAK2–STAT3 axis, promoting secretion of IL-6, GM-CSF, and G-CSF, which enhance the pro-tumorigenic functions of neutrophils and reduce therapeutic sensitivity. **(D)** Mtp53 or p53 loss reprograms myeloid cells toward an immunosuppressive phenotype: ① p53-deficient tumor cells upregulate MCP1, CXCL1/5, and M-CSF, recruiting CD11b^+^ myeloid cells that suppress T cell responses. ② p53 loss drives secretion of IL-34, which—via CD36—enhances fatty acid oxidation in TAMs, promoting “foamy” M2-like polarization. ③ The Mtp53–BRD4 complex enhances CSF-1 secretion, while Mtp53-containing exosomes deliver miR-1246 to macrophages, inducing M2-like reprogramming with elevated PD-L1, IL-6, and TNF-α, facilitating immune escape. ④ These M2-polarized TAMs concurrently suppress both T cell and NK cell anti-tumor activity. **(E)** Mtp53 enhances the pro-tumorigenic functions of CAFs through cell-autonomous and non-autonomous mechanisms: ① Mtp53 expression in CAFs activates STAT3, upregulating α-SMA, FGF10, and CXCL12 to promote tumor migration. ② Aberrant IL-6 secretion confers chemoresistance to tumor cells and suppresses IFN-β production, dampening stromal anti-tumor immunity. ③ p53 loss in tumor cells reduces apelin secretion, weakening inhibitory signaling through the APJ receptor on CAFs and indirectly enhancing CAF activation. ④ The small-molecule conformational corrector pCAP-250 reverses Mtp53-driven pro-tumorigenic effects in CAFs.

In addition, Mtp53 can indirectly modulate immune checkpoint expression to suppress T cell activity. For instance, in urothelial carcinoma, although Mtp53 does not directly activate the IFN-γ signaling pathway, it is frequently associated with high tumor mutational burden and neoantigen load, which promote T cell infiltration and enhanced IFN-γ secretion. Tumor cells then respond to IFN-γ via the JAK–STAT pathway, upregulating PD-L1 expression and establishing a classic “adaptive immune resistance” phenotype. This mechanism partly explains why patients with Mtp53-expressing urothelial carcinomas show better responses to combination therapy with PD-1/PD-L1 inhibitors and chemotherapy (36) (**Figure 3A**). Collectively, Mtp53 suppresses T cells through direct metabolic interference, induction of immune checkpoints, or recruitment of inhibitory immune populations—exhibiting remarkable context dependency and multidimensional regulation.

##### 3.2.2.2 ·Natural killer cells

Mtp53 disrupts natural killer (NK) cell–mediated immune surveillance through multiple mechanisms, thereby facilitating tumor immune escape. First, intratumoral Mtp53 loses the ability to transcriptionally activate NKG2D ligands, leading to significant downregulation of UL16-binding proteins 1 and 2 (ULBP1 and ULBP2) and enabling tumor cells to evade NK cell recognition (37). This defect is especially prominent in breast cancer models harboring the p53 G242A missense mutation: tumor cells exhibit reduced surface expression of MULT1 (the murine ULBP homolog) and early transcript 1 (RAE-1) family ligands, diminished NK cell infiltration, impaired IFN-γ production, and ultimately escape immune clearance *in vivo* (38).

Second, Mtp53 suppresses activating signals through the DNAX accessory molecule-1 (DNAM-1) pathway. Wild-type p53 directly drives transcription of PVR (CD155) and Nectin-2 (CD112); Mtp53 loses this capacity, resulting in low ligand expression and weakened NK cell costimulation (39). The concurrent impairment of both NKG2D and DNAM-1 pathways—leading to dual ligand deficiency—markedly reduces NK cell degranulation and cytotoxicity.

More importantly, Mtp53 may confer intrinsic resistance to NK cell–mediated killing. Natural killer (NK) cells primarily induce target cell apoptosis by releasing granzyme B, which triggers mitochondrial outer membrane permeabilization (MOMP)—a process critically dependent on the activation of the pro-apoptotic protein BAX (40). Given that wild-type p53 can directly bind to and conformationally activate BAX (41), whereas Mtp53 typically loses this transcription-independent pro-apoptotic function, it is plausible that Mtp53 elevates the threshold for mitochondrial apoptosis. Consequently, even when NK cells successfully recognize tumor targets, they fail to efficiently induce cell death.

Importantly, pharmacological intervention can partially reverse Mtp53-mediated NK suppression. For example, in Mtp53-expressing breast cancer cells, restoration of p53 activity enhances autophagy and sensitizes tumor cells to granzyme B–dependent lysis, thereby improving NK cell killing efficiency (42) (**Figure 3B**). In summary, Mtp53 disrupts the NK cell regulatory network at multiple levels—impairing activation, ligand expression, and susceptibility to killing—and promotes immune escape. Restoring p53 function or targeting its downstream effectors may offer new avenues to enhance NK cell–mediated antitumor immunity.

##### 3.2.2.3 ·Tumor-associated neutrophils

Mtp53 plays a pivotal role in recruiting neutrophils and modulating their functional polarization, profoundly influencing pro-tumorigenic signaling. Studies in PDAC models show that Mtp53 drives significant enrichment of CD11b^+^Ly6G^+^ neutrophils while reducing the proportions of CD3^+^ T cells, CD8^+^ T cells, and CD4^+^ Th1 cells, thereby establishing a neutrophil-dominated immunosuppressive microenvironment (43). Similarly, in breast cancer models, p53 loss stimulates tumor cells to secrete WNT ligands, which in turn induce stromal cells (e.g., fibroblasts) to release IL-1β, triggering systemic inflammation and recruiting activated neutrophils that accelerate metastasis and immune evasion (30).

Moreover, in p53-deficient pancreatic tumors, activation of the JAK2–STAT3 pathway promotes massive secretion of cytokines such as IL-6, granulocyte-macrophage colony-stimulating factor (GM-CSF), and granulocyte colony-stimulating factor (G-CSF), indirectly enhancing neutrophil recruitment and pro-tumor functions (44). Notably, Mtp53 upregulates chemokine ligands including CXCL1, CXCL2, and CXCL5, sustaining continuous neutrophil recruitment and local accumulation within tumors—a change that may reduce therapeutic sensitivity(**Figure 3C**). Antibody-mediated neutrophil depletion has been shown to effectively restore tumor responsiveness to immunotherapy (43), suggesting that targeting neutrophils or their recruitment pathways could be a promising strategy to overcome Mtp53-associated immunosuppression and treatment resistance.

##### 3.2.2.4 ·Tumor-associated macrophages and myeloid-derived suppressor cells

Macrophages can polarize into pro-inflammatory M1 (classically activated) or immunosuppressive M2 (alternatively activated) phenotypes, with M2 polarization strongly linked to immune suppression and tumor progression. Evidence indicates that p53 loss or mutation drives macrophage polarization toward the M2 phenotype through multiple mechanisms, thereby fostering an immunosuppressive TME.

In PDAC models, tumor cells with complete p53 deletion markedly upregulate chemokines such as CCL2 (MCP1), CXCL1, CXCL5, and M-CSF, recruiting CD11b^+^ myeloid cells and reprogramming them into an immunosuppressive state that inhibits T cell function (33). In hepatocellular carcinoma, p53-inactivated tumor cells secrete IL-34, which enhances fatty acid oxidation in TAMs in a CD36-dependent manner, promoting their conversion into lipid-laden “foamy” M2-like cells that potently suppress CD8^+^ T cell activity and accelerate immune escape (45).

Mtp53 with gain-of-function (GOF) properties exhibits even stronger pro-tumorigenic effects. In breast cancer, Mtp53 interacts with bromodomain-containing protein 4 (BRD4) to promote secretion of colony-stimulating factor 1 (CSF-1), establishing a novel BRD4–CSF-1 signaling axis that drives TAM infiltration and lung metastasis (46). In colorectal cancer, Mtp53 tumor cells release exosomes enriched in miR-1246, which are taken up by macrophages and suppress TERF2IP, leading to NF-κB activation, M2-like reprogramming, and upregulation of PD-L1, IL-6, and TNF-α—further amplifying local immunosuppression (47).

Notably, M2 macrophages not only directly inhibit T cell responses but also impair NK cell antitumor activity, helping tumors construct a multi-layered immunosuppressive network (48) (**Figure 3D**). Together, these findings suggest that targeting M2 polarization may represent a promising therapeutic approach to reverse immunosuppressive TME.

#### 3.2.3 Cancer-associated fibroblasts

Cancer-associated fibroblasts (CAFs) are a major cellular component of the tumor stroma and play critical roles in tumor progression. Mtp53 regulates CAF pro-tumorigenic functions through both direct and indirect mechanisms. In a breast cancer model, fibroblasts harboring the p53^N^²³^6S^ mutation exhibited activation of the STAT3 signaling pathway and upregulated α-smooth muscle actin (α-SMA), fibroblast growth factor 10 (FGF10), and CXCL12, thereby enhancing tumor cell migration in an *in vitro* co-culture system (49). In gastric cancer, stable overexpression of Mtp53 in CAFs confers chemoresistance to tumor cells through aberrant IL-6 secretion (50).

Beyond cytokine regulation, the p53 status of tumor cells can influence CAF activation via paracrine signaling. For example, in gastric cancer, p53-inactivated tumor cells reduce apelin secretion, weakening APJ receptor signaling on CAFs and thereby enhancing their activation and pro-tumor capacity (51). Moreover, GOF Mtp53-expressing fibroblasts exhibit significantly increased secretion of stromal cell-derived factor-1 (SDF-1/CXCL12), with stronger tumor-promoting effects than p53 loss alone (52).

Mtp53 also compromises the antitumor immune functions of stromal cells. For instance, Mtp53-expressing fibroblasts secrete markedly less interferon-beta (IFN-β), which may attenuate their growth-inhibitory effects on neighboring tumor cells (53).

In Li-Fraumeni syndrome models, fibroblasts that undergo loss of heterozygosity (LOH) and accumulate Mtp53 significantly enhance the malignant phenotype of adjacent cancer cells. Conversely, treatment with the small-molecule p53 conformational corrector pCAP-250 reverses CAF-mediated tumor promotion (54), providing a potential therapeutic rationale for targeting the Mtp53–CAF axis (**Figure 3E**).

## 4 The metabolism–epigenetics–immunity axis: the core triad regulated by Mtp53

Mtp53 is not merely a loss-of-function tumor suppressor but functions as a bona fide oncoprotein with gain-of-function (GOF) properties that systematically reprograms the TIME. It integrates metabolic dysregulation, epigenetic remodeling, and immune suppression into a highly coordinated “metabolism–epigenetics–immunity axis.” This tripartite network not only endows cancer cells with proliferative advantages and stress adaptability but also establishes an immunosuppressive ecosystem that promotes invasion, metastasis, and therapeutic resistance. The following sections detail how Mtp53 orchestrates this immunosuppressive landscape through glycolytic reprogramming, oxidative phosphorylation and redox regulation, lipid metabolism dysregulation, amino acid uptake, and epigenetic control.

### 4.1 Metabolic reprogramming: constructing a pro-tumorigenic and immunosuppressive microenvironment

Mtp53 reprograms tumor cell metabolism to meet energetic and biosynthetic demands while actively reshaping the microenvironment to impair immune cell function. Its core strategy involves creating a hostile niche characterized by nutrient deprivation, acidosis, and elevated oxidative stress—conditions that collectively hinder immune cell survival and activation. The following subsections delineate four interconnected metabolic dimensions through which Mtp53 coordinates and sustains immunosuppression.

#### 4.1.1 Glycolytic reprogramming and lactate production

Accumulating evidence indicates that Mtp53 potently enhances glycolytic flux through multiple mechanisms, conferring growth and invasive advantages to tumor cells while simultaneously shaping a profoundly immunosuppressive TME via lactate accumulation—thereby promoting therapy resistance and metastasis.

**Figure 4:**
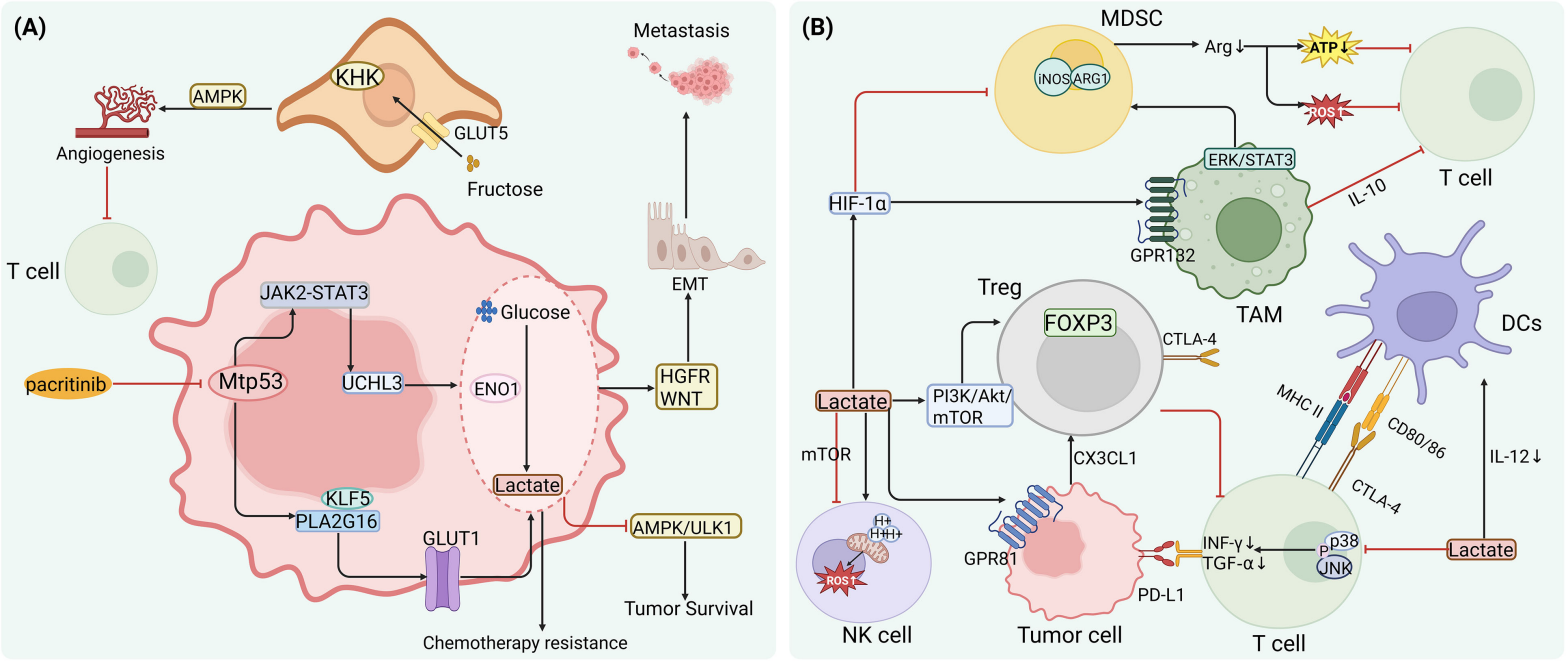
Mtp53 drives immune evasion by enhancing glycolysis and lactate accumulation. **(A)** Mtp53 reinforces glycolysis and supports tumor survival through multiple pathways: ① Activates the JAK2–STAT3–UCHL3 axis to stabilize ENO1, promoting lactate production and conferring chemoresistance (reversible by pacritinib). ② Cooperates with KLF5 to activate PLA2G16, facilitating GLUT1 membrane translocation and increasing glucose uptake and lactate accumulation. ③ Suppresses the AMPK/ULK1 pathway, amplifying glycolysis while blocking autophagy. ④ Activates hepatocyte growth factor receptor (HGFR) and WNT signaling to drive epithelial–mesenchymal transition (EMT) and promote metastasis. ⑤ Fructose metabolism via SLC2A5 (GLUT5)/KHK activates AMPK, inducing aberrant angiogenesis that impedes T cell infiltration. **(B)** Lactate acts as a key immunosuppressive metabolite, broadly impairing anti-tumor immunity: ① Inhibits p38/JNK phosphorylation in T cells, reducing IFN-γ and TNF-α production, and upregulates tumor PD-L1 via the lactate receptor GPR81. ② Specifically enhances CTLA-4 and FOXP3 expression in regulatory T cells (Tregs) and upregulates CX3CL1 to promote their recruitment. ③ Induces intracellular acidification and mitochondrial damage in NK cells, suppresses mTOR activity, and impairs cytotoxic function. ④ Downregulates MHC-II, CD80/86, and IL-12 in dendritic cells (DCs), weakening cytotoxic T lymphocyte (CTL) priming. ⑤ In MDSCs, lactate stabilizes HIF-1α to drive ARG1 and iNOS expression and ROS production; in TAMs, it signals through GPR132 to activate ERK–STAT3, inducing IL-10 secretion—collectively promoting M2 polarization and T cell suppression.

In colorectal cancer, Mtp53 activates the JAK2–STAT3 signaling pathway, upregulating ubiquitin C-terminal hydrolase L3 (UCHL3). UCHL3 stabilizes enolase 1 (ENO1) through deubiquitination, driving a hyper-glycolytic state that generates abundant lactate and ATP, ultimately inducing resistance to 5-fluorouracil (5-FU). Pharmacological inhibition of JAK2 with pacritinib effectively disrupts this axis, reverses metabolic reprogramming, and restores chemosensitivity (55). Notably, ENO1 also exerts non-metabolic pro-invasive functions: in lung cancer, it activates hepatocyte growth factor receptor (HGFR) and WNT signaling to drive EMT, facilitating extracellular matrix degradation and metastasis (56). In pancreatic cancer, Mtp53 forms a complex with the transcription factor Krüppel-like factor 5 (KLF5) to directly activate phospholipase A2 group XVI (PLA2G16) transcription, promoting glucose transporter 1 (GLUT1) translocation to the plasma membrane, increasing glucose uptake, and enhancing lactate accumulation to fuel tumor proliferation (57). Moreover, in TP53-mutant PDAC, high expression of solute carrier family 45 member 4 (SLC45A4) further amplifies glycolysis and suppresses autophagy via inhibition of the AMPK/ULK1 pathway, enabling tumor cell survival under metabolic stress—a hallmark of Mtp53-driven adaptive evolution (58). Interestingly, fructose metabolism also contributes to vascular remodeling. In tumor endothelial cells, fructose is imported via solute carrier family 2 member 5 (SLC2A5, also known as GLUT5) and metabolized by ketohexokinase (KHK), activating AMPK signaling to enhance mitochondrial respiration and angiogenesis. The resulting abnormal vasculature forms a physical barrier that impedes effector T cell infiltration (59) (**Figure 4A**).

As the key end-product of glycolysis, lactate serves as a central mediator linking Mtp53-driven metabolic reprogramming to immune suppression. Lactate severely impairs T cell function by inhibiting phosphorylation of p38 and JNK, thereby reducing IFN-γ and TNF-α secretion (60); Moreover, lactate can upregulate the expression of immune checkpoint molecules such as PD-L1 in tumor cells, further suppressing T-cell activity (61). Critically, lactate modulates RNA splicing to specifically increase CTLA-4 expression in Tregs, thereby strengthening their suppressive capacity over effector T cells (62). Furthermore, lactate binding to its receptor G-protein-coupled receptor 81 (GPR81) upregulates PD-L1 expression on tumor cells, suppressing CD8^+^ T cell cytotoxicity (63).

In NK cells, lactate induces intracellular acidification, disrupts mitochondrial function, exacerbates oxidative stress, and triggers apoptosis; it also inhibits mTOR signaling, impairing NK cell activation and effector functions (64). In dendritic cells (DCs), lactate suppresses MHC class II and co-stimulatory molecules (e.g., CD80, CD86) and limits IL-12 production, thereby weakening their ability to prime cytotoxic T lymphocytes (CTLs) (65, 66). In Tregs, lactate activates the PI3K/Akt/mTOR pathway to enhance FOXP3 expression, reinforcing their immunosuppressive phenotype. Additionally, in gastric cancer, lactate signaling through GPR81 upregulates CX3C motif chemokine ligand 1 (CX3CL1), promoting Treg recruitment and further intensifying immune resistance (67, 68). In MDSCs, lactate stabilizes hypoxia-inducible factor 1-alpha (HIF-1α), driving high expression of arginase 1 (ARG1) and inducible nitric oxide synthase (iNOS), which deplete arginine and generate reactive oxygen species (ROS) and nitric oxide (NO)—directly damaging T and NK cell functions (69).

In TAMs, lactate promotes polarization toward an M2-like phenotype by activating the GPR132 receptor and downstream ERK/STAT3 signaling, leading to increased secretion of vascular endothelial growth factor (VEGF), arginase 1 (ARG1), and IL-10 (70, 71). These M2-polarized TAMs release immunosuppressive cytokines such as IL-10, which inhibit T-cell function and collectively establish a highly immunosuppressive tumor immune microenvironment (TIME) (**Figure 4B**). Collectively, Mtp53 leverages enhanced glycolysis and lactate secretion to simultaneously drive tumor malignancy and erect an immunosuppressive barrier. Targeting this metabolism–immunity axis may reverse immune evasion and enhance therapeutic responsiveness.

#### 4.1.2 Remodeling of oxidative phosphorylation and ROS regulation

Mtp53 reshapes mitochondrial oxidative phosphorylation (OXPHOS) and redox homeostasis to lay the metabolic foundation for tumor progression and immune escape. On one hand, Mtp53 can reshape transcriptional and metabolic programs through GOF mechanisms. For example, Mtp53 forms aberrant complexes with NF-Y, altering the transcription of NF-Y target genes and driving dysregulated expression of cell cycle–related genes (72). At the metabolic level, the p53–SCO2 axis is critical for mitochondrial respiration: inhibition or loss of p53 downregulates SCO2, reduces oxygen consumption, and enhances glycolysis, whereas SCO2 reconstitution partially restores respiratory function and hypoxia tolerance (73). Moreover, in tumor models such as cervical cancer, Mtp53 expression is associated with decreased oxidative phosphorylation (OXPHOS), increased glycolysis, and mitochondrial dysfunction (74, 75). Although direct evidence that NF-Y or E2F family members *mediate* Mtp53-dependent transcriptional repression of SCO2 is currently lacking, the well-documented capacity of Mtp53 to rewire transcriptional networks—combined with established data on the p53–SCO2 axis—supports a plausible model: Mtp53 may promote an OXPHOS-deficient phenotype by attenuating p53–SCO2–driven metabolic programs and synergizing with broader metabolic reprogramming (73–75).

On the other hand, in specific contexts, Mtp53 stabilizes peroxisome proliferator-activated receptor gamma coactivator 1-alpha (PGC-1α) protein, paradoxically enhancing OXPHOS to promote metastasis (76). This bidirectional regulation supports tumor proliferation and stress adaptation but also creates metabolic vulnerabilities tied to nutrient dependency (77).

Notably, Mtp53’s regulation of OXPHOS is highly context-dependent: in PDAC, Mtp53 upregulates respiratory chain complex expression, enhances OXPHOS, and promotes mitochondrial fission to drive migration (78); in basal-like breast cancer, Mtp53 suppresses OXPHOS via the miR-200c–phosphoenolpyruvate carboxykinase 2 (PCK2) axis, disrupting the tricarboxylic acid (TCA) cycle and enhancing stemness (79); in colorectal cancer under chemotherapy pressure, the Mtp53-R273H variant boosts mitochondrial biogenesis and OXPHOS activity to mediate therapy resistance and migration (80).

**Figure 5:**
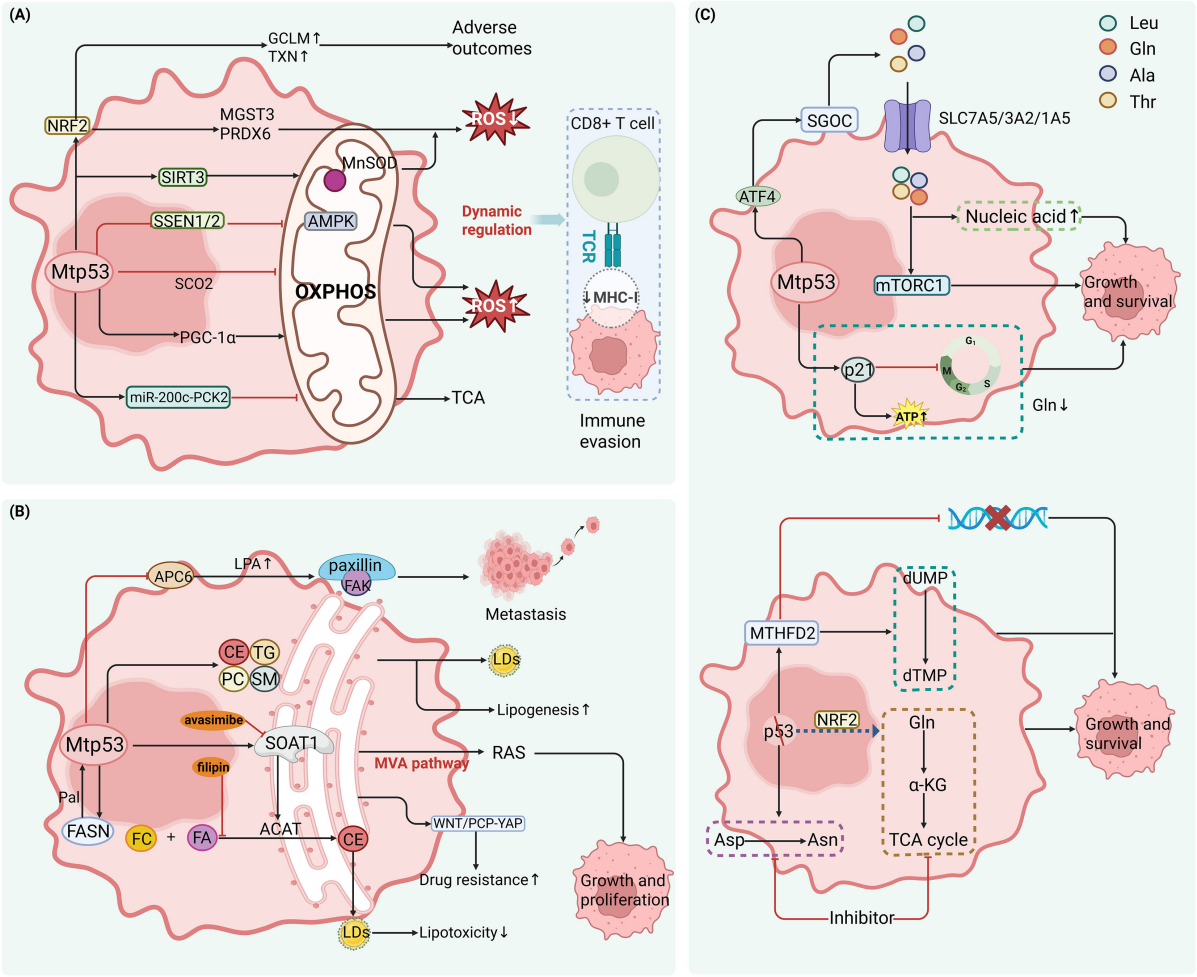
Mtp53 shapes an immunosuppressive tumor microenvironment by reprogramming oxidative phosphorylation, lipid, and amino acid metabolism. **(A)** Mtp53 bidirectionally regulates OXPHOS and fine-tunes ROS homeostasis: ① Represses respiratory chain genes (e.g., SCO2), dampening oxidative phosphorylation (OXPHOS). ② In specific contexts, stabilizes PGC-1α to enhance OXPHOS and promote metastasis, or activates the miR-200c–PCK2 axis to suppress OXPHOS and reinforce stemness. ③ Induces the SIRT3–MnSOD axis to mitigate oxidative damage and activates NRF2 to upregulate TXN, GCLM, PRDX6, and MGST3; simultaneously suppresses SLC7A11 and SESN1/2, maintaining ROS within a pro-survival “safe window.” **(B)** Mtp53 drives mutation-specific lipid reprogramming: ① Upregulates SOAT1 to esterify free cholesterol, preventing lipotoxicity and sustaining mevalonate (MVA) pathway flux and RAS membrane localization (Mtp53-harboring PDAC is sensitive to avasimibe). ② R175H enriches monounsaturated fatty acid (MUFA)-modified cholesteryl esters (CE) and triglycerides (TG); R273H elevates phosphatidylcholine (PC) and sphingolipids—both associated with lipid droplet accumulation. ③ Forms a positive feedback loop with FASN: palmitate promotes S-palmitoylation and stabilization of Mtp53, while Mtp53 transcriptionally upregulates FASN. ④ Downregulates ACP6, leading to lysophosphatidic acid (LPA) accumulation that activates FAK/paxillin to drive metastasis; co-treatment with filipin blocks WNT/PCP–YAP-mediated resistance triggered by SOAT1 inhibition. **(C)** Mtp53 and p53 loss differentially rewire amino acid metabolism: ① Mtp53 stabilizes ATF4, activating the serine–glycine–one-carbon (SGOC) pathway and upregulating SLC7A5/3A2/1A5 to enhance serine/glycine synthesis and leucine/glutamine uptake, fueling mTORC1 and nucleotide biosynthesis. ② Under glutamine deprivation, Mtp53 induces p21 to slow cell cycle progression and preserve ATP, promoting survival. ③ p53 loss upregulates MTHFD2 to boost one-carbon flux and support dTMP synthesis. ④ Mtp53 may cooperate with NRF2 to create dependency on specific amino acids (e.g., asparagine or glutamine), revealing a targetable metabolic vulnerability.

At the redox level, Mtp53 fine-tunes reactive oxygen species (ROS) levels to maintain a pro-tumorigenic microenvironment. For example, in melanoma, Mtp53 induces sirtuin 3 (SIRT3) expression, promoting deacetylation of manganese superoxide dismutase (MnSOD) to reduce oxidative damage and support survival (81). In breast cancer, Mtp53 exhibits selective regulation of nuclear factor erythroid 2-related factor 2 (NRF2) target genes: it upregulates thioredoxin (TXN) and the glutamate–cysteine ligase modifier subunit (GCLM), while suppressing solute carrier family 7 member 11 (SLC7A11). High TXN expression correlates significantly with poor patient prognosis (82). Additionally, Mtp53 inhibits sestrin 1/2 (SESN1/2), weakening AMPK signaling and leading to mitochondrial ROS accumulation (83). In triple-negative breast cancer, Mtp53 cooperates with NRF2 to upregulate microsomal glutathione S-transferase 3 (MGST3) and peroxiredoxin 6 (PRDX6), counteracting ferroptosis and other forms of oxidative stress–induced cell death (84) (**Figure 5A**).

Together, these mechanisms demonstrate that Mtp53 maintains ROS within a “safe window” that favors tumor adaptation. Thus, Mtp53 coordinates energy metabolism with antioxidant defense—not only conferring survival advantages but also exposing metabolic dependencies that can be therapeutically exploited through targeting SIRT3, NRF2 pathways, or induction of ferroptosis.

#### 4.1.3 Lipid metabolism dysregulation

Mtp53 profoundly rewires lipid metabolism to supply tumor cells with structural components, energy reserves, and signaling molecules, while simultaneously fostering a tumor microenvironment that favors immune evasion and therapeutic resistance. In PDAC, tumor cells harboring Mtp53 exhibit upregulated expression of sterol O-acyltransferase 1 (SOAT1, also known as ACAT1), an enzyme that esterifies free cholesterol into cholesteryl esters. This process mitigates lipotoxicity and maintains homeostasis of mevalonate (MVA) pathway intermediates—such as geranylgeranyl pyrophosphate (GGPP)—thereby supporting membrane localization of oncoproteins like RAS (85). Notably, studies in PDAC mouse models and organoids have demonstrated that tumors carrying Mtp53 (R172H, equivalent to human R175H) are highly sensitive to the SOAT1 inhibitor avasimibe, whereas p53 wild-type tumors show minimal response (85), suggesting that SOAT1 represents a metabolic vulnerability specific to Mtp53-driven tumors.

Different hotspot Mtp53 mutants drive distinct lipidomic phenotypes. Cotton et al. performed lipidomic profiling in PDAC cells and found that the R175H mutant enriches monounsaturated fatty acid (MUFA)-containing triglycerides and cholesteryl esters, whereas R273H elevates phosphatidylcholine and sphingolipid levels; both mutants exhibit increased lipid droplet accumulation and enhanced *de novo* fatty acid synthesis (86). These findings indicate that Mtp53 gain-of-function (GOF) activities are mutation-specific, although broader *in vivo* validation is needed to assess their generalizability.

Moreover, a feedback loop exists between Mtp53 and lipid metabolism. Liu et al. reported that in multiple cancer cell lines, palmitate produced by fatty acid synthase (FASN) stabilizes Mtp53 via S-palmitoylation and enhances its interaction with transcriptional coactivators. ChIP-qPCR confirmed Mtp53 enrichment at the FASN promoter, and Mtp53 overexpression was shown to upregulate FASN mRNA (87). While these data suggest a potential positive feedback circuit, its necessity in tumorigenesis remains unproven due to the lack of *in vivo* models or mutation-specific rescue experiments.

In high-grade serous ovarian carcinoma, Mtp53 downregulates the lysophosphatidic acid phosphatase ACP6, leading to autocrine accumulation of lysophosphatidic acid (LPA), which activates the FAK/paxillin pathway to promote migration and peritoneal metastasis (88). This study, based on cell line models and correlative analyses of clinical samples, provides preliminary evidence for Mtp53-mediated regulation of phospholipid signaling.

Importantly, while SOAT1 inhibition effectively kills Mtp53-expressing tumors, the resulting buildup of free cholesterol may inadvertently activate the WNT/PCP–YAP signaling axis, triggering adaptive resistance. Co-treatment with a cholesterol-chelating agent (e.g., filipin) blocks this compensatory activation and significantly enhances therapeutic efficacy (89) (**Figure 5B**).

In summary, current evidence indicates that Mtp53 influences lipid metabolism through multiple mechanisms—including modulation of key nodes such as SOAT1, FASN, and ACP6. Targeting these pathways shows therapeutic promise in specific genetic contexts; however, most proposed mechanisms still require rigorous *in vivo* validation to establish their causality and general applicability.

#### 4.1.4 Amino acid uptake

Tumor cells frequently reside in nutrient-poor microenvironments, and TP53 abnormalities, whether gain-of-function resulting from missense mutations or loss-of-function due to complete deletion, can confer a significant survival advantage by reprogramming amino acid metabolism.Mtp53 not only loses its tumor-suppressive function but actively drives metabolic adaptation. In TNBC, Mtp53 mutants such as R175H and R280K have been shown to stabilize ATF4 protein and upregulate key enzymes in the serine–glycine–one-carbon (SGOC) metabolic pathway—including PHGDH and PSAT1—as well as amino acid transporters (SLC7A5, SLC3A2, and SLC1A5). Functional studies demonstrated that these alterations enhance *de novo* serine/glycine synthesis and increase uptake of leucine and glutamine, thereby supporting mTORC1 activation and nucleotide biosynthesis, and promoting tumor growth under nutrient-limited conditions (90). This study, based on Mtp53 knock-in cell lines, xenograft models, and metabolomic profiling, provides a relatively comprehensive mechanistic evidence chain.

Additionally, under glutamine deprivation, Mtp53 has been reported to induce p21 expression, slowing cell cycle progression while preserving mitochondrial membrane potential and ATP levels, thereby enhancing cancer cell survival (91). However, this work was primarily conducted *in vitro* and did not establish whether p21 acts downstream of the Mtp53–ATF4 axis; thus, the integration of this pathway with SGOC metabolism requires further validation.

In contrast, p53 loss—lacking GOF activity—derepresses metabolic genes. Li et al. showed in p53-null mouse embryonic fibroblasts and colon cancer models that p53 deficiency leads to upregulation of MTHFD2, increasing one-carbon flux to promote dTMP synthesis, alleviate replication stress, and reduce DNA damage (92). Direct transcriptional repression by p53 was confirmed by ChIP-qPCR, making this a canonical example of metabolism rewiring driven by p53 LOF.

Recent studies further suggest that TP53 aberrations may confer specific amino acid dependencies. In patient-derived organoids and PDX models of castration-resistant prostate cancer (CRPC), tumors with TP53 mutation or deletion exhibited heightened dependence on asparagine, and asparaginase treatment selectively induced their death (93), implicating asparagine metabolism as a potential vulnerability in TP53-altered CRPC.

Regarding glutamine dependency, Hamada et al. found in KRAS-mutant pancreatic cancer that NRF2 activation enhances glutamine metabolism and increases sensitivity to glutaminase inhibitors (94) (**Figure 5C**). Although TP53 mutations frequently co-occur with NRF2 pathway dysregulation, this study did not directly assess the role of Mtp53; therefore, whether Mtp53 cooperates to amplify glutamine dependence remains to be verified.

In summary, current evidence indicates that Mtp53 actively reprograms amino acid metabolism via the ATF4–SGOC axis, whereas p53 loss enhances one-carbon flux through derepression of MTHFD2. Although mechanistically distinct, both scenarios can lead to dependency on specific amino acids—such as asparagine or glutamine. Nevertheless, certain associations (e.g., the Mtp53–NRF2–glutamine axis) remain in early exploratory stages and require more *in vivo* causal studies for confirmation.

### 4.2 Epigenetic hijacking: locking cells into an immunosuppressive state

Beyond metabolic regulation, Mtp53 also acts as a chromatin regulatory hub, systematically manipulating epigenetic mechanisms to lock cancer cells into a pro-oncogenic and immunosuppressive transcriptional state. By recruiting DNA methyltransferases, histone-modifying enzymes, and non-coding RNA regulators, Mtp53 remodels gene expression programs, reinforces oncogenic traits, and remotely regulates immune cell activity, thereby promoting immune escape. The following sections detail how Mtp53 reshapes chromatin structure through coordinated control of DNA and histone modifications and reconstructs non-coding RNA networks to drive immune escape.

#### 4.2.1 DNA methylation and histone modifications

Mtp53 profoundly reshapes the tumor epigenetic landscape by modulating DNA methylation and histone modifications, thereby driving malignant progression and mediating immune evasion. Regarding DNA methylation, Guo et al. reported in human breast cancer cell lines that Mtp53 (R175H and R273H) interacts with DNMT1 and enhances its methyltransferase activity at the CDKN2A/p16 promoter, leading to transcriptional silencing of p16 (95). However, this study relied primarily on co-immunoprecipitation and methylation-specific PCR, without providing whole-genome DNA methylation profiling or *in vivo* functional validation; thus, the generalizability of this mechanism remains to be confirmed.

At the level of histone modifications, Efe et al. used ChIP-seq in triple-negative breast cancer and pancreatic cancer models to demonstrate that Mtp53 co-occupies the CSF1 enhancer with BRD4, promoting H3K27ac enrichment and transcriptional activation of CSF1. Secreted CSF-1 recruits and polarizes TAMs, induces STAT3 phosphorylation, and thereby drives epithelial–mesenchymal transition (EMT) and metastasis (46). This study integrated *in vivo* metastasis assays with single-cell RNA-seq, establishing a relatively complete mechanistic chain.

Rahnamoun et al., through ChIP-seq and CRISPRi screening in colon cancer cells, found that Mtp53 interacts with the histone methyltransferase MLL4 and co-localizes with it at enhancers of inflammatory and invasive genes such as MMP9 and CCL2, facilitating H3K4me1 deposition and establishing an active enhancer state (96). Although this work provides genome-wide binding evidence, it lacks functional validation using Mtp53–MLL4 interaction–deficient mutants, and the pathological relevance *in vivo* awaits confirmation in animal models.

**Figure 6:**
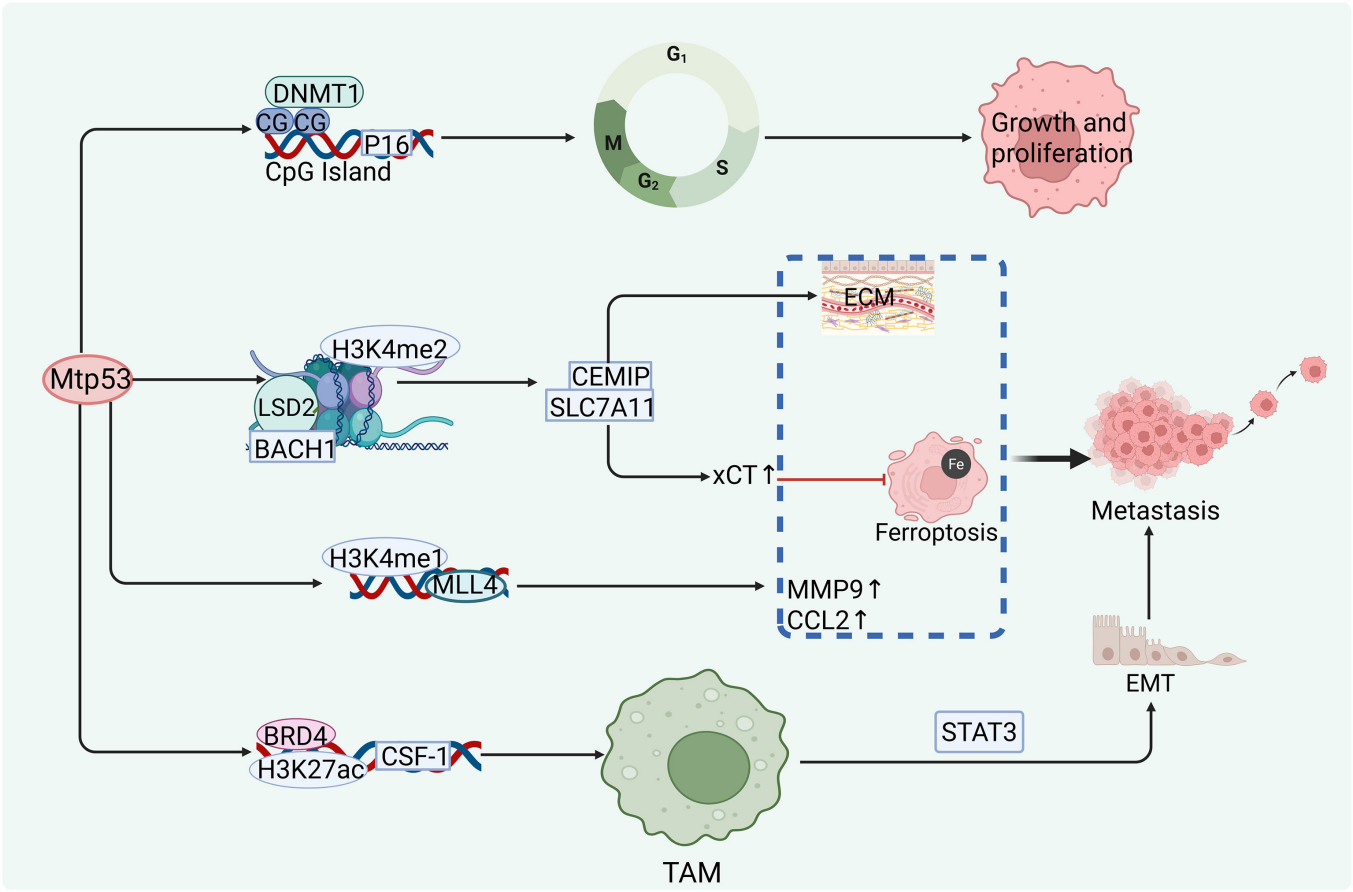
Mtp53 cooperates with multiple epigenetic regulators to drive cell cycle dysregulation, immune suppression, and metastasis. ① Mtp53 binds DNMT1 to induce hypermethylation of the CDKN2A/p16 promoter, silencing its expression and promoting tumor proliferation and dedifferentiation. ② Mtp53 co-occupies the CSF1 enhancer with BRD4, enriching H3K27ac to activate CSF-1 secretion, which paracrinely stimulates the TAM–STAT3 axis, driving EMT and metastasis. ③ Mtp53 collaborates with MLL4 to deposit H3K4me1 at enhancers of MMP9 and CCL2, enhancing extracellular matrix degradation and inflammatory cell recruitment. ④ Mtp53 acts as a scaffold to form a ternary complex with BACH1 and LSD2: at the SLC7A11 locus, LSD2 demethylates H3K4me2 to relieve transcriptional repression and suppress ferroptosis; at the CEMIP locus, the complex enhances BACH1-mediated transactivation to promote invasion.

Notably, Mtp53 mutants with distinct conformational states can selectively recruit epigenetic regulatory complexes to achieve bidirectional gene regulation. For example, the hotspot mutant Mtp53-R175H acts as a molecular scaffold, forming a ternary complex with the transcription factor BACH1 and the histone demethylase lysine-specific demethylase 2 (LSD2, also known as KDM1B). At the SLC7A11 promoter, LSD2 catalyzes demethylation of H3K4me2, relieving BACH1-mediated transcriptional repression and upregulating xCT expression to suppress ferroptosis. Conversely, at loci such as CEMIP, the same complex enhances BACH1’s transcriptional activation function, promoting extracellular matrix degradation and tumor invasion (97) (**Figure 6**).

In summary, current evidence indicates that Mtp53 can reshape local chromatin states by recruiting epigenetic regulators—including DNMT1, BRD4, MLL4, and LSD2. Among these, the CSF1 axis and the SLC7A11/CEMIP regulatory module are supported by relatively robust functional validation, whereas the roles of p16 promoter methylation and MLL4-dependent enhancer activation require further *in vivo* causal studies for confirmation.

#### 4.2.2 Non-coding RNA networks

Mtp53 not only loses the wild-type regulatory functions over non-coding RNAs (ncRNAs), but also actively rewires the expression networks of long non-coding RNAs (lncRNAs), microRNAs (miRNAs), and circular RNAs (circRNAs) through GOF mechanisms, thereby cooperatively driving EMT, tumor stemness, migration, invasion, and immune evasion. To clarify this complex regulatory landscape, we systematically organize current evidence by ncRNA class and their dominant biological functions (**Table 1**).

**Table 1:** Mtp53-regulated non-coding RNA network: classification by ncRNA type, cancer context, mechanism, and biological function.

ncRNA type	ncRNA Name	Cancer type	Key mechanism	Biological function	Reference(s)
lncRNA	LINC00857	Pancreatic ductal adenocarcinoma (PDAC)	Acts as a scaffold bridging FOXM1 and deubiquitinase OTUB1 → stabilizes FOXM1	EMT, migration, metastasis	(98)
	LINC00460	Pancreatic ductal adenocarcinoma (PDAC)	Sponges USP10; activates miR-4689/UBE2V1 axis	Proliferation, p53 stabilization	(99)
	CARMN	Colorectal cancer (CRC)	m^6^A hypermethylation → RNA decay → loss of miR-5683 sponge → FGF2 upregulation	Proliferation, migration	(100)
	MIR205HG	Head and neck squamous cell carcinoma (HNSCC)	Sponges miR-590-3p → de-repression of Cyclin B, CDK1, and YAP	Cell cycle progression, proliferation	(101)
miRNA	miR-182-5p	Pan-cancer	Directly transactivated by Mtp53 → targets FOXO1 and MITF	Migration, invasion	(102)
	miR-155	Breast cancer	Upregulated by Mtp53	EMT, metastasis	(103)
	miR-223-3p	Lung squamous cell carcinoma (LSCC)	Targets Mtp53 mRNA; forms a negative feedback loop	Tumor suppression, inhibition of invasion	(106)
circRNA	circCFL1	Triple-negative breast cancer (TNBC)	Scaffold for HDAC1–c-Myc interaction → c-Myc deacetylation/stabilization → enhances Mtp53 transcription	Stemness, EMT, immune evasion	(107)
	circPVT1	Head and neck squamous cell carcinoma (HNSCC)	Transcriptionally activated by Mtp53/YAP/TEAD complex	Proliferation, anti-apoptosis	(108)
	circ-Ccnb1	Breast cancer	Binds H2AX and Bclaf1 → disrupts Mtp53 GOF protein interactions	Tumor suppression	(109)

##### 4.2.2.1 lncRNAs: scaffold-mediated transcriptional and post-translational regulation

Mtp53 frequently activates oncogenic lncRNAs by directly binding to their promoters or modulating epigenetic modifications. In PDAC, Mtp53 directly transactivates *LINC00857*, which acts as a protein scaffold bridging the transcription factor FOXM1 and the deubiquitinase OTUB1. This stabilizes FOXM1 and induces EMT, with increased N-cadherin and decreased E-cadherin, thereby promoting metastasis (98). Similarly, under hypoxic conditions, Mtp53 cooperates with HIF-1α to upregulate LINC00460.This lncRNA enhances proliferation via the miR-4689/UBE2V1 axis and simultaneously sequesters the deubiquitinase USP10 to stabilize p53, establishing a positive feedback loop that drives PDAC progression (99).

In colorectal cancer (CRC), Mtp53 (e.g., R273H) directly represses the m^6^A demethylase *ALKBH5*, leading to hypermethylation of the lncRNA *CARMN* and its subsequent degradation by YTHDF2/3. Loss of *CARMN*—which normally functions as a “sponge” for miR-5683—reduces free miR-5683 levels, thereby derepressing *FGF2* and activating the PI3K/Akt/mTOR pathway to drive proliferation and migration (100). Additionally, in head and neck squamous cell carcinoma (HNSCC), *MIR205HG* sequesters miR-590-3p, releasing key effectors of the cell cycle (Cyclin B, CDK1) and Hippo pathway (YAP), accelerating tumor progression (101).

##### 4.2.2.2 miRNAs: direct transcriptional control and global biogenesis suppression

Beyond indirect regulation (e.g., via lncRNA sponges), Mtp53 can directly drive miRNA transcription or suppress their biogenesis. For instance, Mtp53 directly upregulates *miR-182-5p* in multiple cancer types; this miRNA targets several tumor suppressors (e.g., *FOXO1*, *MITF*), markedly enhancing migration and invasion (102). Similarly, in breast cancer, Mtp53 activates *miR-155* to promote EMT and metastasis (103).

More broadly, Mtp53 disrupts global miRNA processing: it binds to and inhibits the Microprocessor complex (Drosha–DGCR8), blocking pri-miRNA-to-pre-miRNA conversion (104), and may also modulate *Dicer* expression to impair mature miRNA production (105). Critically, certain miRNAs reciprocally regulate Mtp53 stability, forming feedback loops. In lung squamous cell carcinoma, *miR-223-3p* directly targets Mtp53 mRNA to suppress its expression, while Mtp53 represses *miR-223-3p* transcription—establishing a double-negative feedback loop. Disruption of this circuit leads to Mtp53 accumulation and tumor progression, whereas restoration of *miR-223-3p* effectively suppresses invasion (106).

##### 4.2.2.3 circRNAs: conformationally stable signaling hubs and therapeutic targets

Due to their covalently closed, highly stable structure, circRNAs serve as ideal mediators for Mtp53 to establish persistent malignant phenotypes. In TNBC, *circCFL1* acts as a scaffold that enhances the interaction between HDAC1 and c-Myc, promoting deacetylation of c-Myc at K148 and inhibiting its ubiquitin-mediated degradation. Stabilized c-Myc further transactivates *Mtp53*, creating an Mtp53–circCFL1–c-Myc positive feedback loop that ultimately activates the p-AKT/WIP/YAP/TAZ axis and induces both EMT and stemness markers (e.g., ALDH1A1, CD44^+^/CD24^-^) (107).

In HNSCC, *circPVT1* is directly activated by the Mtp53/YAP/TEAD transcriptional complex; its overexpression promotes proliferation and suppresses apoptosis (108). Conversely, *circ-Ccnb1* exerts tumor-suppressive effects in breast cancer: by binding H2AX and Bclaf1, it disrupts Mtp53’s interactions with these proteins, thereby inhibiting Mtp53 GOF-driven tumorigenesis (109). This finding suggests that not all circRNAs are oncogenic—some endogenous circRNAs may constitute a natural inhibitory network against Mtp53.

### 4.3 Crosstalk between metabolites and the epigenome: how metabolites shape the epigenetic landscape of TIME

Metabolic reprogramming driven by Mtp53 not only confers proliferative and survival advantages to tumor cells but also remotely shapes the tumor immune microenvironment through the accumulation of specific metabolic byproducts. Metabolites such as lactate, α-ketoglutarate (α-KG), and acetyl-CoA have transcended their canonical roles in energy production or biosynthesis and now function as key signaling molecules or direct substrates for epigenetic modifications, dynamically regulating the expression programs of immune-related genes. By covalently modifying histones—through lysine lactylation, acetylation, or demethylation—or non-histone proteins such as PD-L1 and TNFR2, these metabolites persistently lock myeloid and T cells into immunosuppressive or exhausted states without altering the DNA sequence, thereby promoting immune evasion.

Notably, although multiple studies in non-Mtp53 contexts have established causal links within the “metabolism–epigenetics–immunity axis” through integrated interventions targeting metabolic flux, site-specific epigenetic marks, and immune function, no study to date has systematically examined the integrity of this axis in the setting of Mtp53-driven tumors. This represents a critical knowledge gap in understanding how Mtp53 GOF activity reshapes the TIME (**Table 2**).

**Table 2:** Evidence strength classification of key nodes in the metabolism–epigenetics–immunity axis.

Metabolism–Epigenetics–Immunity pathway	Tumor model	Key experimental intervention	Immune phenotypic outcome	Evidence strength classification	Reference
Lactate → H3K18la (IL10/TGFB) → TAM M2 polarization → T-cell suppression	Glioblastoma (human/mouse)	LDHA knockout in macrophages; scRNA-seq + CUT&Tag	Reduced T-cell proliferation suppression; ↓ M2 markers	Causal closed-loop validation (lactate flux manipulation → lactylation dynamics → immune function)	(110)
Lactate → H3K9la (CXCL9/10) → CD8^+^T-cell exclusion → anti-PD-1 resistance	Multiple solid tumors (mouse)	Overexpression of de-lactylating enzyme; lactate transport inhibition	↑ CD8^+^T-cell infiltration; enhanced response to immunotherapy	Causal closed-loop validation	(111)
Lactate → TNFR2-K122la → Treg stability ↑ → immunosuppression	Malignant pleural effusion (human/mouse)	TNFR2-K122R site-directed mutagenesis	↓ Treg expansion; reduced immunosuppressive function	Causal closed-loop validation (non-histone lactylation)	(112)
STAT5 → H3K18la (PD-L1) → T-cell suppression	Acute myeloid leukemia	STAT5 knockout; exogenous lactate treatment	↓ PD-L1; enhanced T-cell cytotoxicity	Strong associative evidence (no direct manipulation of lactate production)	(113)
Lactate → H3K18la (CCL18) → TAM recruitment → tumor progression	Ovarian cancer	Lactate treatment; ChIP-qPCR	↑ CCL18 secretion; ↑ TAM infiltration	Strong associative evidence (lack of lactate flux or lactylase intervention)	(114)
Lactate → CBX3–H3K18la → silencing of antigen presentation genes → CD8^+^T-cell loss	Glioblastoma	CBX3 inhibition/knockdown	↑ Antigen presentation; ↑ CD8^+^T-cell infiltration	Strong associative evidence (lactate not verified as rate-limiting substrate)	(115)
Lactate → H3K18la → METTL3 ↑ → m^6^A-JAK/STAT → M2 polarization	Not specified (review-based hypothesis)	No original experimental validation	—	Hypothesis awaiting validation	(116)
α-KG → TET-dependent demethylation of PD-L1 promoter → PD-L1 expression → CD8^+^ T-cell exclusion	Renal cell carcinoma (mouse)	GLS inhibition or IDH2 knockdown in tumor cells; hMeDIP-seq/5mC analysis	↑ CD8^+^T-cell infiltration; enhanced anti-PD-1 efficacy	Causal closed-loop validation (α-KG flux manipulation → DNA demethylation dynamics → immune evasion)	(120)
α-KG → KDM6B-mediated H3K27me3 demethylation at Il12b/Tnf promoters → M1 macrophage activation → pro-inflammatory microenvironment	Non-tumor infection model (primary mouse macrophages)	Exogenous α-KG supplementation; ChIP-qPCR for H3K27me3	↑ IL-12 secretion; ↑ M1 markers; enhanced bacterial clearance	Causal closed-loop validation (α-KG intervention → histone demethylation dynamics → immune output)	(121)
α-KG → Th1/Treg balance regulation (mechanism unclear)	*In vitro* CD4^+^T-cell differentiation system	Glutamine deprivation or α-KG supplementation	↑ Th1/↓ Treg; upregulated T-bet expression	Strong associative evidence (metabolism–immune phenotype link without epigenetic dynamics)	(122)
α-KG → Treg suppression via mitochondrial metabolism and lipid remodeling	*In vitro* Treg induction system	α-KG treatment	↓ Treg differentiation efficiency	Strong associative evidence (non-epigenetic metabolism–immune association)	(123)
Acetyl-CoA → H3K27ac (FasLG promoter) → sFasL secretion → CD8^+^ T-cell apoptosis	Pancreatic neuroendocrine tumors (PNETs, mouse/human-derived)	ACSS2 inhibitor; FasL-neutralizing antibody; CUT&Tag for H3K27ac	↑ CD8^+^T-cell infiltration; tumor growth suppression	Causal closed-loop validation (Acetyl-CoA flux manipulation → histone acetylation dynamics → immune function)	(130)
Acetyl-CoA → PD-L1-K162/181ac → PD-L1 protein stabilization → T-cell suppression	Melanoma (mouse/human-derived)	ACLY inhibition; PD-L1 acetylation-site mutants (K162R/K181R)	↓ PD-L1 protein; ↑ CD8^+^ T-cell activation	Causal closed-loop validation (non-histone acetylation; protein stability regulation)	(127)
Acetyl-CoA → (presumed H3K27ac) → PD-L1 transcription → immune evasion	Liver cancer (MASH-HCC mouse model)	ACLY inhibitor treatment	↓ PD-L1 mRNA; ↑ CD8^+^T-cell infiltration; enhanced anti-PD-1 response	Strong associative evidence (metabolic intervention + immune output, but no site-specific acetylation measured)	(128)
Acetate → ACSS2 → Acetyl-CoA → c-Myc ↑ → PD-L1 transcription → impaired T-cell killing	Multiple solid tumors (*in vitro*/mouse)	Acetate treatment; c-Myc knockdown	↑ PD-L1 expression; ↓ T-cell function	Strong associative evidence (no direct manipulation of endogenous Acetyl-CoA flux or acetylation detection)	(129)

#### 4.3.1 The lactate-driven metabolism–epigenetics–immunity axis: from association to causality

In the context of Mtp53-driven Warburg effect, lactate is not merely an end product of glycolysis but also a direct substrate for lysine lactylation (Kla), enabling covalent modification of histones and non-histone proteins to modulate immune responses within the tumor microenvironment. Recent studies have sought to dissect the causal logic of the triad “lactate → site-specific Kla → immunosuppression,” with evidence ranging from mechanistic correlations to fully validated causal chains.

The most compelling causal evidence comes from glioblastoma and solid tumor models. De Leo et al. knocked out *LDHA* in human monocyte-derived macrophages, substantially reducing intracellular lactate levels. Using CUT&Tag, they demonstrated a concomitant decrease in H3K18la enrichment at the promoters of *IL10* and *TGFB*. Functionally, these macrophages exhibited significantly impaired capacity to suppress T-cell proliferation. Integrated with single-cell transcriptomics, this study established a complete causal chain within a single experimental system: reduced lactate → decreased H3K18la → weakened immunosuppressive function (110). Similarly, Wang et al. found in multiple murine solid tumor models that intratumoral H3K9la levels positively correlated with microenvironmental lactate concentration. Overexpression of a catalytically active HDAC3 mutant with delactylase activity or pharmacological inhibition of lactate transporters effectively reduced H3K9la, restored transcription of *CXCL9* and *CXCL10*, increased CD8^+^ T-cell infiltration, and markedly enhanced response to anti–PD-1 therapy—thereby closing the functional axis “lactate → H3K9la → T-cell exclusion → immunotherapy resistance” *in vivo (*111). Furthermore, Xue et al. demonstrated in clinical samples of malignant pleural effusion and murine models that a high-lactate microenvironment induces lysine lactylation of TNFR2 at K122 in Tregs. By engineering a non-lactylatable TNFR2-K122R mutant, they effectively blocked lactate-induced stabilization of TNFR2 and downstream NF-κB activation, significantly impairing Treg expansion and immunosuppressive function—thus establishing, for the first time, a causal link between lactate-driven lysine lactylation of a non-histone protein and immune regulation (112).

In contrast, other studies, while providing mechanistically plausible associations, have not yet completed the full trajectory from lactate flux manipulation to immune phenotypic output. For example, Huang et al. used ChIP-qPCR in acute myeloid leukemia cell lines and patient samples to show that STAT5 recruits p300 to the *CD274* (PD-L1) promoter, promoting H3K18la deposition and driving its expression; exogenous lactate enhanced this modification, whereas *STAT5* knockout reduced both H3K18la and PD-L1 levels (113). However, this study did not directly perturb endogenous lactate production or clearance, leaving it unclear whether lactate serves as the rate-limiting metabolic substrate in this pathway. Sun et al. reported in ovarian cancer–associated macrophages that lactate treatment induced H3K18la enrichment at the *CCL18* promoter and upregulated its expression, as confirmed by ChIP-seq and qPCR (114). Separately, Wang et al. identified CBX3 as a “reader” of H3K18la via proteomics and ChIP-seq, and showed that CBX3 inhibition or shRNA knockdown reversed silencing of antigen-presentation genes and promoted immune evasion in glioblastoma (115). Although these studies validated the functional importance of Kla through chromatin immunoprecipitation, rescue experiments, or targeted interventions, none systematically modulated lactate flux (e.g., via *LDHA* knockout or MCT inhibition), and thus could not establish lactate as a necessary upstream driver—representing strong, mechanistically coherent indirect evidence rather than complete causal chains.

Notably, earlier reviews proposed two hypotheses: that H3K18la might upregulate METTL3 to modulate m^6^A modification of JAK/STAT pathway components, or that lactylation of moesin enhances TGF-β signaling (116). Although logically sound, no primary study to date has provided direct evidence—through ChIP, site-directed mutagenesis, metabolic flux tracing, or *in vivo* models—to substantiate these ideas, leaving them as theoretical propositions awaiting validation.

In summary, while the “lactate → Kla → immunosuppression” axis has garnered experimental support across multiple tumor types, the most rigorous causal evidence derives exclusively from non-Mtp53-driven contexts. These studies employed *Ldha* knockout, overexpression of delactylases, or Kla-site mutagenesis to specifically perturb lactate levels or Kla status, observing reversible changes in both epigenetic marks and immune function. To date, however, no study has tested this axis in an Mtp53-driven tumor model by conditionally deleting *Ldha* or inhibiting MCT1/4 transporters while simultaneously monitoring dynamic changes in lysine lactylation (e.g., via quantitative mass spectrometry or site-specific antibodies) alongside immune cell infiltration and therapeutic response—thereby failing to fully validate the causal sequence: “Mtp53 activation → lactate accumulation → Kla modification → immune evasion.” Nevertheless, myeloid cells—particularly tumor-associated macrophages—remain widely regarded as central effectors of this axis due to their high metabolic plasticity (117), providing a rationale for targeting the lactate–Kla pathway in immunotherapy.

#### 4.3.2 α-Ketoglutarate: an emerging regulator in the metabolism–epigenetics–immunity axis

α-Ketoglutarate (α-KG) is a key intermediate of the tricarboxylic acid (TCA) cycle and an essential cofactor for Jumonji C (JmjC) domain–containing histone demethylases (KDMs) and TET-family DNA demethylases (118, 119). As a co-substrate for these epigenetic enzymes, α-KG levels directly influence histone and DNA methylation states, thereby regulating the expression of immune-related genes. Recent research has progressed from correlative observations between metabolites and immune phenotypes toward causal validation of the triad “α-KG → site-specific demethylation → immune functional output.” However, the strength of current evidence varies considerably, necessitating clear distinctions among fully established causal chains, strongly associated mechanistic links, and unverified theoretical models.

The strongest causal evidence comes from integrated models involving tumor cells and myeloid immune cells. Li et al. pharmacologically inhibited glutaminase (GLS) or genetically knocked down *IDH2* in renal cell carcinoma to specifically reduce endogenous α-KG levels. This intervention markedly impaired TET1/2-mediated DNA demethylation at the *PD-L1* (*CD274*) promoter (evidenced by decreased 5hmC and increased 5mC), leading to PD-L1 downregulation. Functionally, this resulted in significantly enhanced CD8^+^ T-cell infiltration and dramatically improved response to anti–PD-1 immunotherapy (120). This study completed a full causal chain within a single *in vivo* model: reduced α-KG → diminished TET activity → hypermethylation of the *PD-L1* promoter → attenuated immune evasion—representing the most rigorous experimental validation of the α-KG–driven metabolism–epigenetics–immunity axis to date.

In tumor-associated macrophages, Liu et al. found that exogenous α-KG promoted M1-like activation and suppressed M2 polarization. Mechanistically, α-KG enhanced the histone demethylase activity of KDM6B (Jmjd3), significantly reducing H3K27me3 levels at promoters of pro-inflammatory genes (e.g., *Il12b*, *Tnf*), as directly confirmed by ChIP-qPCR. Functionally, these macrophages secreted more IL-12 and exhibited enhanced antimicrobial capacity (121). Although this study did not assess T-cell responses in a tumor setting, it established a causal link in primary immune cells: α-KG → H3K27me3 demethylation → enhanced pro-inflammatory function—providing critical support for α-KG as a means to reverse immunosuppressive microenvironments.

In contrast, other studies have revealed α-KG’s role in shaping T-cell fate but have not directly validated the epigenetic intermediary step. Klysz et al. showed that glutamine-derived α-KG upregulated T-bet (encoded by *TBX21*) in *in vitro*–activated CD4^+^ T cells, promoting Th1 differentiation and suppressing regulatory T-cell (Treg) generation (122); Matias et al. reported that α-KG inhibits Treg differentiation by remodeling mitochondrial metabolism and lipid homeostasis (123). However, neither study examined DNA or histone methylation status at key immune loci such as *FOXP3* or *TBX21*, leaving the epigenetic mechanism unconfirmed. These works represent strong associative evidence with plausible mechanisms but lack the epigenetic link required to establish α-KG as a necessary upstream driver.

In summary, the “α-KG → demethylation → immune modulation” axis has received preliminary causal support in renal cancer and macrophage models (120, 121), yet all robust evidence to date originates outside Mtp53-driven genetic contexts. No study has yet conditionally deleted *Idh1/2* or *Glud1*, or inhibited glutamine transporters, in Mtp53-mutant tumors while simultaneously tracking dynamic changes at specific epigenetic sites (e.g., 5hmC at the *PD-L1* promoter or H3K27me3 at the *FOXP3* CNS2 region) alongside immune cell infiltration and therapeutic response—thus failing to fully validate the causal sequence: “Mtp53 activation → α-KG accumulation → enhanced demethylation → immune evasion or dysregulation.” Nevertheless, given the central roles of TET and KDM enzymes in both T cells and myeloid cells (124), α-KG remains a promising targetable metabolic node for combined epigenetic and immunotherapeutic strategies.

#### 4.3.3 Acetyl-CoA: an epigenetic substrate driving immune evasion programs via FasL and PD-L1

Acetyl-CoA serves as a critical substrate for histone acetyltransferases (HATs), allowing metabolic flux to be transduced into chromatin acetylation signals that rapidly regulate transcriptional outputs of immune-related genes (125, 126). Recent studies reveal that acetyl-CoA drives multiple immune evasion programs through two complementary mechanisms: transcriptional activation via histone acetylation and protein stabilization via non-histone acetylation. The existing evidence exhibits a clear gradient in causal rigor, requiring careful distinction between validated closed-loop pathways and mechanistically plausible but incompletely connected associative models.

The most compelling causal evidence comes from pancreatic neuroendocrine tumors (PNETs) and melanoma. Dang et al. demonstrated in PNETs that activation of the acetyl-CoA synthetase ACSS2 markedly elevated local acetyl-CoA levels, promoting H3K27ac enrichment and chromatin accessibility at the *FasLG* promoter. Concurrently, the anti-apoptotic factor AATF was recruited to this locus, cooperatively activating *FasLG* transcription and triggering massive secretion of soluble FasL (sFasL), which induced apoptosis of CD8^+^ T cells upon binding to Fas. Critically, pharmacological inhibition of ACSS2 or neutralization of FasL restored CD8^+^ T-cell infiltration and suppressed tumor growth. This study established a complete causal chain within one model: elevated acetyl-CoA → increased H3K27ac → upregulated FasL transcription → enhanced T-cell apoptosis—representing a paradigmatic closed-loop example of histone acetylation–mediated immune evasion (130).

In melanoma, Wang et al. found that nuclear-cytosolic acetyl-CoA enables p300 to directly acetylate PD-L1 at lysine residues K162 and K181, significantly enhancing its protein stability by inhibiting ubiquitin–proteasomal degradation. Reducing acetyl-CoA levels with an ACLY inhibitor reversed PD-L1 acetylation and restored CD8^+^ T-cell function (127). This work established, for the first time, a causal axis whereby acetyl-CoA promotes immune evasion through non-histone acetylation–mediated stabilization of an immune checkpoint molecule, thereby expanding the functional scope of acetyl-CoA in immune regulation.

By contrast, other studies offer mechanistically reasonable but incompletely closed associative evidence. Gautam et al. reported in a hepatocellular carcinoma model that ACLY inhibition lowered acetyl-CoA levels, coinciding with PD-L1 downregulation and increased CD8^+^ T-cell infiltration (128); Wang et al. showed that exogenous acetate, converted to acetyl-CoA by ACSS2, indirectly enhanced PD-L1 transcription via c-Myc upregulation, thereby impairing T-cell cytotoxicity (129). Although these studies observed clear immune phenotypic changes following metabolic intervention, neither directly measured dynamic modifications at specific sites (e.g., H3K27ac at the *CD274* promoter or PD-L1 protein acetylation), and thus cannot confirm acetyl-CoA as a necessary upstream driver—constituting strong associative, but not fully causal, evidence.

In summary, the mechanisms by which acetyl-CoA promotes immune evasion through histone or non-histone acetylation of FasL and PD-L1 have received causal support in PNETs and melanoma (127, 130), yet all current evidence lacks validation in Mtp53-driven genetic contexts. No study has yet conditionally deleted *Acly* or *Acss2*, or inhibited acetate transporters, in Mtp53-mutant tumors while simultaneously tracking dynamic changes in H3K27ac at the *FasLG* promoter, PD-L1 acetylation status, and CD8^+^ T-cell fate alongside immunotherapy response—thus failing to fully close the integrative causal chain: “Mtp53 activation → acetyl-CoA accumulation → multi-layered acetylation modifications → immune evasion.” Nonetheless, targeting acetyl-CoA metabolic pathways (e.g., with ACLY or ACSS2 inhibitors) holds promise for concurrently suppressing both FasL- and PD-L1–mediated immunosuppressive mechanisms, offering a novel strategy to reprogram the anti-tumor immune microenvironment.

#### 4.3.4 Integrated perspective: metabolites as bridges in immune epigenetic regulation

In summary, although lactate, α-ketoglutarate (α-KG), and acetyl-CoA have been convincingly shown in various non-Mtp53 models to drive immune evasion through lysine lactylation, demethylation, or acetylation, the causal integrity of these “metabolism–epigenetics–immunity axes” has not yet been directly validated in the context of Mtp53-driven tumorigenesis. Nevertheless, given that Mtp53 profoundly rewires tumor cell metabolism and promotes the accumulation of these metabolites, it is plausible that Mtp53 co-opts these metabolic byproducts to convert intrinsic metabolic reprogramming into distal immunomodulatory signals, thereby systematically sculpting an immunosuppressive microenvironment.

Specifically, lactate can promote the expression of immunosuppressive factors or T-cell exclusion via H3K18la or H3K9la (110, 111); α-KG can drive DNA or histone demethylation at loci such as *PD-L1* or pro-inflammatory genes in a TET- or KDM-dependent manner (120, 121); and acetyl-CoA can either activate *FasL* transcription through H3K27ac or enhance PD-L1 protein stability via direct acetylation (127, 130). Although these pathways target distinct molecular effectors, they share a core mechanistic principle: fluctuations in metabolite concentrations are sensed by epigenetic enzymes and translated into stable, functional immune phenotypes.

This “metabolism–epigenetics–immunity axis” provides a compelling conceptual framework for understanding how Mtp53 GOF activity non-genetically reprograms host immunity. It suggests that Mtp53 may amplify its oncogenic impact through metabolic byproducts, while simultaneously revealing multiple druggable nodes that are independent of the p53 mutation itself. For instance, pharmacological inhibition of LDHA, ACLY, or GLS—or direct targeting of lactyltransferases, acetyltransferases, or demethylases—may disrupt the homeostasis of the tumor immune microenvironment, reverse immunosuppression, and potentiate immune checkpoint blockade.

Future studies must therefore employ Mtp53-driven orthotopic tumor models to simultaneously track metabolic flux, site-specific epigenetic dynamics (e.g., H3K18la, 5hmC at the *PD-L1* promoter, or H3K27ac at the *FasLG* locus), and immune cell fate—including infiltration, exhaustion, and functional responsiveness—to causally validate this integrated axis. Such efforts will lay the foundation for precision immuno-metabolic interventions tailored to Mtp53-mutant cancers.

## 5 Cancer-type specificity, co-mutation context, and heterogeneity in Mtp53-driven immune regulation

Although *TP53* is the most frequently mutated gene in human cancers, Mtp53 is not a functionally homogeneous entity. On the contrary, Mtp53 actively reshapes the TIME through GOF activities, and this process exhibits profound context dependency. The ultimate immunological phenotype is jointly determined by three key dimensions: the specific Mtp53 mutation subtype (e.g., conformational mutant R175H versus DNA-contact mutant R273H), the tissue-of-origin–defined basal transcriptional program, and recurrent co-occurring driver events (such as *KRAS* activation in pancreatic cancer or *MYC* amplification in triple-negative breast cancer). These factors intertwine into a complex epistatic network, leading Mtp53 to deploy distinct strategies for immune evasion and metabolic reprogramming across different cancer types. Therefore, treating Mtp53 merely as a generic poor-prognosis marker severely underestimates its potential as a therapeutically actionable target and a dynamic biomarker for monitoring. This section systematically dissects the mechanistic heterogeneity of Mtp53 across major solid tumors, integrating mutation subtypes, co-mutation landscapes, and TIME features to construct a multidimensional stratification framework oriented toward clinical translation—providing both theoretical rationale and practical pathways for personalized immuno-metabolic interventions based on Mtp53 status (**Table 3**).

**Table 3:** Cancer type–specific mechanisms, co-mutation contexts, and translatable biomarkers of Mtp53-mediated immune evasion.

Cancer type	Common Co-mutations	Dominant Mtp53 mechanism(s)	Key immune phenotype	Predictive biomarkers (Clinical Readiness)	Targeted strategies
TNBC	MYC↑, PTEN−	p53–miR-34a–PD-L1 axis; BRD4–CSF1 enhancer activation; Treg/NK modulation	TIL-rich but exhausted; TAM^+^; Treg^+^	High: TP53 mut subtype (NGS panel) (134); PD-L1 IHC (131, 137) Medium: ctDNA VAF (∼0.01% limit) (148)	ICB ± BRD4 inhibitor (e.g., JQ1)
PDAC	KRAS^G12D^	NF-κB–CXCL1 → PMN-MDSC expansion; SOAT1↑ → cholesterol esterification; KLF5–PLA2G16 → glycolysis	T-cell excluded; PMN-MDSC^+^; fibrotic stroma	High: TP53 mut (NGS) (134) Medium: Serum CXCL1/CCL2; ctDNA dynamics (147, 148)	CXCR2 inhibitor + CSF1R inhibitor + avasimibe (SOAT1i)
NSCLC	EGFRmut, mTORC1↑	p53–miR-34a–PD-L1 axis; hotspot mutations predict gemcitabine response (133)	Variable TILs; PD-L1^+^/^-^ (context-dependent)	High: TP53 hotspot status (133); PD-L1 IHC Medium: ctDNA for clonal tracking (147, 148)	Gemcitabine (if hotspot+) ± ICB
CRC	APC−, KRASmut	Exosomal miR-1246 → M2 TAM polarization; CAF → IL-6 secretion; clonal selection under therapy (146)	Myeloid suppression; M2-TAM^+^; T-cell dysfunction	Medium: ctDNA MRD (post-op) (146); exosomal miR-1246 High: TP53 mut (NGS) (134)	Anti-IL-6 (siltuximab) + TAM-depleting agents
HCC	CTNNB1mut, mTORC1↑	TP53/mTORC1 axis → PD-L1 degradation; potential immune activation (136)	Inflamed or excluded (heterogeneous); PD-L1 variable	High: TP53 mut + mTORC1 status (IHC/NGS) Caution: mTOR inhibitors may ↑ PD-L1 in TP53-mut HCC	Avoid mTORi monotherapy if TP53-mut; consider ICB + VEGF inhibitor

### 5.1 Cancer-type specificity of Mtp53-mediated immune regulation and its molecular basis

The mechanisms by which Mtp53 modulates the TIME vary substantially across cancer types. This heterogeneity stems from tissue-specific transcriptional programs, unique cellular ecosystems, and characteristic co-occurring driver mutations. In TNBC, Mtp53 primarily promotes immune evasion through the “p53–miR-34a–PD-L1 axis”: loss of wild-type p53 function leads to downregulation of miR-34a, thereby relieving post-transcriptional repression of programmed death-ligand 1 (PD-L1) and resulting in its aberrant overexpression on tumor cells. Additionally, Mtp53—particularly the R175H variant—cooperates with BRD4 to bind the *CSF1* enhancer, driving infiltration of TAMs, enhancing Tregs recruitment, and suppressing NK cells activity, thereby establishing a multilayered immunosuppressive network (46, 131).

In contrast, in pancreatic ductal adenocarcinoma (PDAC), Mtp53 almost invariably co-occurs with activating *KRAS* mutations. The two synergistically activate the NF-κB pathway, enabling Mtp53 recruitment to the *CXCL1* enhancer and triggering massive CXCL1 secretion to expand PMN-MDSCs—a hallmark of the T-cell–excluded “cold tumor” phenotype. Moreover, Mtp53 upregulates SOAT1 to maintain cholesterol ester homeostasis, supporting RAS membrane localization and further amplifying oncogenic signaling (21, 85, 86).

In CRC, Mtp53 relies more heavily on extracellular vesicle–mediated long-range communication—specifically by driving tumor cells to release exosomes enriched in miR-1246. Upon uptake by macrophages, these exosomes promote M2-like polarization and activate CAFs to secrete IL-6, ultimately impairing antitumor lymphocyte function (47).

Notably, even different hotspot mutations can drive divergent biological programs. Hassin et al. directly compared R175H and R273H and found only partial overlap in their transcriptional networks: R175H preferentially activated NF-κB and lipid biosynthesis pathways, whereas R273H predominantly affected DNA repair–related genes—highlighting functional divergence among Mtp53 subtypes (132).

In NSCLC, our team recently observed that patients harboring *TP53* hotspot mutations (e.g., R248W, R273H) exhibited significantly better responses to gemcitabine, suggesting that Mtp53 status not only influences immune evasion but may also guide chemotherapy selection (133).

Collectively, these mechanisms—spanning direct signaling, immune cell recruitment, and intercellular crosstalk—demonstrate that Mtp53 does not act through a single universal pathway. Instead, it dynamically tailors its immunoregulatory strategy according to the specific features of each tumor microenvironment, exhibiting remarkable contextual plasticity.

### 5.2 Development of predictive biomarkers integrating mutation subtype and co-mutation context

Given the high prevalence of *TP53* mutations in aggressive cancers (e.g., ∼80% in TNBC and >70% in PDAC) and the central role of Mtp53 in actively sculpting an immunosuppressive microenvironment, Mtp53 holds substantial clinical potential as a predictive biomarker. Currently, targeted next-generation sequencing (NGS) panels (e.g., 24-gene platforms) routinely used in clinical practice can reliably detect *TP53* mutation status and hotspot subtypes (such as R175H or R273H) in formalin-fixed paraffin-embedded (FFPE) tumor samples, with validated sensitivity and specificity in multiple solid tumors including gastric cancer (134).

In TNBC and NSCLC, dysregulation of the “p53–miR-34a–PD-L1 axis” represents a key immune escape mechanism: Mtp53-mediated loss of miR-34a relieves translational repression of PD-L1, leading to its upregulation. This pathway is conserved across multiple cancer types, and restoration of miR-34a has been shown to enhance tumor-infiltrating lymphocyte (TIL) accumulation and alleviate T-cell exhaustion (131, 135). However, Mtp53’s regulation of PD-L1 can be complex and even bidirectional—e.g., in hepatocellular carcinoma (HCC), the TP53/mTORC1 axis paradoxically promotes PD-L1 degradation, suggesting that mTORC1 inhibition in this context might yield counterintuitive effects and underscoring the necessity of treatment personalization based on p53 status and co-mutation background (136). Clinically, *TP53* mutation correlates significantly with elevated PD-L1 expression in breast cancer, further supporting its value as a functional biomarker (137).

Compared with conventional biomarkers such as tumor mutational burden (TMB) or microsatellite instability (MSI), Mtp53 offers distinct advantages: it directly regulates PD-L1 through well-defined transcriptional mechanisms (e.g., via miR-34a), and its GOF activity systemically remodels the TIME. Thus, Mtp53 status may not only predict response to immune checkpoint inhibitors but also inform combination strategies targeting Mtp53 itself. Crucially, because Mtp53 actively drives immunosuppression, therapeutic targeting of its function—whether by restoring wild-type activity or inhibiting GOF effects—has the potential to not only reverse PD-L1 overexpression but also broadly reshape the immune landscape, providing a robust mechanistic foundation for synergistic combination therapies.

### 5.3 Dynamic monitoring of Mtp53 clonal evolution to counteract acquired resistance

To fully realize the clinical utility of Mtp53, a paradigm shift is required—from a static, binary assessment of “mutation present or absent” toward a multidimensional, dynamic biomarker platform. The interplay between Mtp53 and the TIME is not fixed; rather, it represents a continuously co-evolving process under therapeutic pressure. Tumors constitute heterogeneous ecosystems composed of competing clones, and treatment imposes strong selective pressures that may not only eliminate existing Mtp53 clones but also favor the emergence of subclones with newly acquired gain-of-function (GOF) properties or secondary mutations.

In small-cell lung cancer (SCLC), longitudinal genomic analyses have revealed that chemotherapy and immunotherapy profoundly reshape clonal architecture. In some patients, novel *TP53* subclones emerge or pre-existing ones expand—events tightly correlated with the development of an immunosuppressive microenvironment, including T-cell depletion, accumulation of MDSCs and Tregs, and downregulation of IFN-γ signaling (138).

In CRC, *TP53*-mutant clones are frequently positively selected and expanded under therapy, eventually dominating the tumor population and conferring resistance to both chemotherapy and targeted agents (139). Similarly, in *TP53/BRCA1* co-deficient models, PARP inhibitor treatment induces heterogeneous resistant clones, some of which escape therapy by restoring genomic stability or activating alternative DNA repair pathways—highlighting the remarkable adaptive evolutionary capacity of Mtp53-driven tumors (140).

In pediatric acute lymphoblastic leukemia (ALL), 78% of post-transplant relapse cases exhibit significant clonal replacement and branched evolution, demonstrating that therapy can fundamentally reconfigure the tumor genome (141). Even *TP53* mutant clones present at low frequency at diagnosis (variant allele frequency [VAF] <5%) can be positively selected during chemotherapy or targeted therapy, expand to become dominant, and ultimately drive treatment failure (142). Moreover, *TP53*-mutant clones may actively reshape the microenvironment through cytokine secretion, suppressing drug-sensitive competitors and establishing themselves as “ecologically dominant” populations (143).

Circulating tumor DNA (ctDNA) analysis provides a critical tool for monitoring this dynamic process. In biliary tract cancer, ctDNA profiling has revealed substantial fluctuations in *TP53* mutant clone abundance during treatment, with molecular changes preceding radiographic progression and correlating with clinical deterioration (144). In locally advanced NSCLC, the rate of ctDNA clearance has been shown to predict the magnitude of benefit from consolidative immunotherapy (145). In CRC, ctDNA is already being used for postoperative minimal residual disease (MRD) detection and early relapse warning, with molecular signals appearing months before imaging-based progression (146). The latest-generation MRD platforms achieve detection limits of approximately 0.01% VAF, and analytical validation studies have confirmed their robustness and reliability (147, 148).

We therefore recommend serial plasma collection—at baseline and every 6–8 weeks during treatment—for dynamic ctDNA tracking to capture shifts in Mtp53 clonal abundance, provide early warning of TIME remodeling, and guide timely therapeutic intervention.

Consequently, future research must move beyond the simplistic “mutant/wild-type” dichotomy and instead focus on the evolutionary trajectories of Mtp53 clones and their spatiotemporal control over the TIME. Only through such systems-level understanding can we effectively anticipate and overcome resistance to immunotherapy (149).

## 6 Therapeutic prospects: from functional restoration to synthetic lethality and immune combination

Mtp53 is one of the most frequent genetic alterations in human cancers and, in theory, represents a highly promising therapeutic target. However, translating its biological properties into reproducible clinical benefit remains a formidable challenge. Although therapeutic strategies have expanded beyond functional restoration to include protein degradation, read-through therapies, synthetic lethality, and combination immunotherapies, the vast majority remain in preclinical or early-phase clinical development, with no agent yet approved by the U.S. Food and Drug Administration (FDA) or the European Medicines Agency (EMA). This persistent translational gap underscores a disconnect between basic research and clinical efficacy: *in vitro* activity or early-phase responses often fail to predict success in confirmatory clinical endpoints. To address this, we systematically review the mechanisms of action, clinical progress, and limitations of current major therapeutic approaches, aiming to identify the root causes of this translational chasm and explore potential paths forward (**Table 4**).

**Table 4:** Therapeutic strategies and clinical investigations targeting p53 in oncology.

Clinical trial identifier	Therapeutic agent	Mechanism of action	Cancer type	Stage	Reference
NCT03072043	APR-246+Aza	Covalently binding to the cysteine residue of Mtp53, restoring the wild-type conformation	AML/MDS	II/III	(152)
NCT03588078			MDS/AML	II	(153)
NCT03745716			MDS	III	(155)
NCT04383938	APR-246+ Pembrolizumab		Advanced solid tumors	I/II	(156)
NCT04906031	SSG	Sb³^+^ binds to the cysteine residue of Mtp53, stabilizes the conformation and promotes ubiquitination degradation	MDS/AML	II	(160)
NCT01339871	SAHA	Inhibition of HDAC6 promotes HSP90 acetylation, leading to the release of mutant p53 (Mtp53) and its subsequent degradation via MDM2/CHIP-mediated ubiquitin-proteasome pathway.	Metastatic solid tumor	I	(162)
NCT04767984 NCT03560882 NCT03358017	Statins	Inhibit HMGCR, block RhoA isoprene, disrupt Mtp53 stabilized axis	TNBC, ovarian cancer	In progress	(167–169)
–	Bisphosphonates+Statins	Inhibit FPP/GGPP synthesis and block Rho-Mtp53 stable axis	Breast cancer	Preclinical	(166)
NCT01357161	Adavosertib (WEE1i)	Inhibits WEE1, forcing DNA-damaged cells into mitosis	Ovarian cancer	II	(174)
–			mCRC	II (FOCUS4-C)	(173)
NCT02340177	SGT-53	Deliver wild-type TP53 cDNA to restore p53 function	Metastatic pancreatic cancer	I/II	(171)
NCT03554707			CNS malignancy in children	I/II	(172)
NCT04585750	PC14586	Specific binding to p53 Y220C mutant pocket restores wild conformation	Y220C mutant advanced solid tumor	I/II	(158)
NCT01191684	p53MVA vaccine	Viral vector delivers p53 antigen, activates T cell response	Advanced gastrointestinal tumor	I	(175)
NCT03897881	mRNA-4157/V940	Personalized mRNA vaccine encodes neoantigens (with p53 epitopes)	High-risk melanoma	II	(176)

### 6.1 Direct targeting: restoration, degradation, and read-through

Direct intervention on the Mtp53 protein is the most intuitive approach, but its feasibility critically depends on whether wild-type function can be restored or the oncogenic protein selectively eliminated. Given the existence of over a thousand missense mutations and multiple conformational states of Mtp53, developing broadly effective drugs is extremely challenging.

#### 6.1.1 Functional restoration

Functional restoration aims to restore wild-type conformation and transcriptional activity through small-molecule-induced folding. APR-246 (eprenetapopt) is the most clinically advanced candidate in this category. Its active metabolite, MQ, covalently binds to cysteine residues (C124, C229, C277) in the DNA-binding domain of Mtp53, thereby restoring DNA-binding capacity in certain mutant variants (150, 151).

In TP53-mutant myelodysplastic syndromes (MDS) and acute myeloid leukemia (AML), phase II trials (NCT03072043 (152), NCT03588078 (153)) showed high overall response rates (ORR 71%) and complete response rates (CR 44%) with APR-246 in combination with azacitidine, along with reductions in variant allele frequency (VAF), indicating biological activity (154). This depth of response is markedly superior to the historical complete remission (CR) rates observed with azacitidine monotherapy (approximately 20–25%), suggesting that this combination may hold clinical value in specific patient populations. These encouraging results generated high expectations for the broad applicability of this therapy in TP53-mutant hematologic malignancies. However, the pivotal phase III randomized trial (NCT03745716) failed to meet its primary endpoints: no statistically significant differences were observed in CR rate (P = 0.13) or overall survival (OS) (155). This negative result indicates that, in an unselected, broad population of patients with *TP53*-mutant myelodysplastic syndromes (MDS), APR-246 failed to demonstrate clinical benefit beyond standard therapy, and therefore does not support its use as a routine treatment. Notably, the mechanism of action of APR-246 depends on its active metabolite, MQ, which covalently binds to cysteine residues (C124, C229, and C277) within the DNA-binding domain of Mtp53. Consequently, APR-246 is only effective against missense mutations that retain these cysteines and possess a conformationally reversible defect—such as R175H or R273H—but is ineffective against nonsense, frameshift, or structurally disruptive mutations that are not temperature-sensitive (150, 151).

The phase III trial NCT03745716 enrolled all types of *TP53* mutations, including approximately 10–15% of nonsense or frameshift variants, likely diluting any potential efficacy signal. We hypothesize that the primary reason for the phase III failure was inadequate patient stratification rather than intrinsic inefficacy of the drug itself.

To test this hypothesis, future trials should prospectively enrich for *TP53* mutation subtypes known to be sensitive to APR-246 (e.g., missense mutations adjacent to critical cysteines) and exclude patients harboring nonsense or frameshift alterations. For example, a phase II “basket trial” could be designed to enroll only patients with MDS, AML, TNBC, or PDAC who carry *TP53* missense mutations with documented conformational reversibility (as annotated in functional databases such as the IARC TP53 Database), and evaluate the complete remission (CR) rate and depth of variant allele frequency (VAF) clearance with APR-246 combined with standard therapy. Such a biomarker-driven trial design holds promise for reestablishing the clinical utility of this strategy—particularly in solid tumors like TNBC and PDAC.

This outcome not only questions the clinical viability of APR-246 but also highlights a broader translational bottleneck—positive signals in early single-arm studies may be influenced by patient selection bias or transient pharmacodynamic effects and often fail to replicate in larger randomized trials. Potential reasons include heterogeneous responses across Mtp53 subtypes, intrinsic tumor resistance mechanisms, and pharmacokinetic/pharmacodynamic limitations of the drug. Thus, while functional restoration is mechanistically sound, the phase III failure of APR-246 indicates that this strategy has not yet crossed the critical threshold from biological activity to clinical efficacy. Future success may depend on more precise biomarker-driven patient selection and optimized combination regimens.

In contrast, PC14586 exemplifies a “precision targeting” paradigm: it specifically binds to a surface cleft created by the Y220C mutation, stabilizing and restoring p53 function (157). In a phase I/II trial (NCT04585750), PC14586 demonstrated an ORR of approximately 32% in high-dose cohorts among patients with advanced solid tumors harboring the TP53 Y220C mutation, with a favorable safety profile (158). Given that Y220C-mutant solid tumors are typically highly resistant to standard therapies (historical objective response rate [ORR] <10%), this response rate holds clear clinical significance and supports further development. This suggests that structure-guided drug design may overcome the limitations of broad-spectrum restorers. However, Y220C accounts for only 1–2% of all TP53 mutations, indicating a narrow applicable population. Expanding this approach will require the development of additional mutation-specific drugs to broaden the treatable patient population.

Additionally, sodium stibogluconate (SSG) promotes Sb³^+^ binding to the DNA-binding domain (DBD), facilitating recognition by E3 ubiquitin ligases (e.g., CHIP) and subsequent proteasomal degradation—a “stabilize-degrade” dual mechanism (159). A phase II trial (NCT04906031) is currently evaluating its efficacy in temperature-sensitive p53 mutant MDS/AML (160). Although the mechanism is novel, concerns regarding long-term safety and off-target effects remain, and reliable clinical evidence is still lacking.

#### 6.1.2 Protein degradation

Rather than restoring complex transcriptional functions, directly eliminating oncogenic Mtp53 may offer a more universal strategy. Vorinostat, an HDAC6 inhibitor, disrupts the HSP90-Mtp53 chaperone complex, promoting Mtp53 degradation via the MDM2/CHIP-mediated ubiquitin-proteasome pathway (161). A phase I trial (NCT01339871) suggested that vorinostat in combination with pazopanib could counteract Mtp53-driven angiogenesis (162), but confirmatory efficacy data are lacking. Moreover, the general toxicity of HDAC inhibitors limits their long-term use.

Statins (e.g., simvastatin) and bisphosphonates (e.g., zoledronate) inhibit the MVA pathway, blocking the prenylation of Rho GTPases, disrupting the Rho-Mtp53 stabilization axis, and thereby promoting Mtp53 degradation (163–165). In breast cancer models, their combination has shown potential to overcome resistance to HER2-targeted therapies (166). Several clinical trials (NCT04767984, NCT03560882, NCT03358017) are exploring their use in Mtp53 tumors such as triple-negative breast cancer (167–169). Although well-tolerated, systemic metabolic effects may trigger compensatory mechanisms, narrowing the therapeutic window. Prospective efficacy data remain limited.

#### 6.1.3 Read-through therapies

For approximately 10% of TP53 nonsense mutations, read-through therapies aim to induce ribosomes to bypass premature termination codons (PTCs), allowing production of full-length functional p53. Metabolites of 5-FU (e.g., fluorouridine, FUr) can achieve non-specific read-through via mRNA misincorporation (170), but lack target specificity and are unsuitable as precision therapies.

SGT-53 is a liposomal nanoparticle delivering wild-type TP53 cDNA, bypassing the mutation to directly express functional p53 (171, 172). Several clinical trials (NCT02340117, NCT03554707) are evaluating its combination with chemotherapy or radiotherapy. While conceptually appealing, gene therapy faces persistent challenges, including low delivery efficiency, immunogenicity, and transient transgene expression. Its clinical utility remains uncertain, placing it in a highly exploratory category, far from widespread application.

### 6.2 Indirect and combination strategies: exploiting derived vulnerabilities

Given the difficulties in directly targeting Mtp53, increasing efforts are focusing on indirect strategies—exploiting synthetic lethal dependencies or immune microenvironment remodeling resulting from p53 loss, i.e., a “surround and suppress” rather than “direct attack” approach.

#### 6.2.1 Synthetic lethality

Loss of p53 forces tumor cells to rely on the G2/M checkpoint to manage DNA damage. The WEE1 inhibitor adavosertib prevents inhibitory phosphorylation of CDK1/2, forcing cells with DNA damage into premature mitosis, leading to mitotic catastrophe (173). In a phase II trial (NCT01357161) in TP53-mutant ovarian cancer, adavosertib combined with platinum-based chemotherapy significantly improved progression-free survival (PFS: 9.9 *vs*. 8.0 months), although overall survival was not significantly extended and hematologic toxicity increased (174). Although this progression-free survival (PFS) benefit did not translate into an overall survival (OS) advantage, it remains clinically informative in the context of *TP53*-mutant ovarian cancer—a population with an otherwise extremely poor prognosis—particularly for the subset of patients who are platinum-sensitive.

Similar PFS benefits were observed in RAS/TP53 co-mutant metastatic colorectal cancer (FOCUS4-C trial) (173). These data provide relatively strong biological and preliminary clinical support for synthetic lethality, making it one of the most promising indirect strategies. However, balancing efficacy with toxicity remains a key challenge. Future directions include dose optimization and rational combinations with PARP or ATR inhibitors to enhance efficacy and reduce adverse effects.

#### 6.2.2 Immune combination strategies

Restoring Mtp53 alone is often insufficient to eradicate tumors; synergistic effects with immunotherapy are crucial. A phase I trial (NCT04383938) showed that APR-246 combined with pembrolizumab increased CD8+ T cell infiltration and TCR clonal diversity (156), suggesting potential to convert “cold” tumors into “hot” ones. However, the overall response rate was only 8.8%, substantially lower than the historical objective response rate (ORR) of pembrolizumab monotherapy in PD-L1–high non-small cell lung cancer (NSCLC) (∼40%), indicating limited immune activation by this combination in unselected solid tumors—possibly due to deeper immunosuppressive mechanisms such as Treg enrichment, MDSC expansion, or STING pathway silencing. This again illustrates how even rational mechanisms can be thwarted by the complexity of the TIME.

Vaccine-based approaches offer an alternative. The p53MVA vaccine demonstrated safety and immunogenicity in a phase I trial (NCT01191684) (175). More promisingly, personalized mRNA vaccines (e.g., mRNA-4157/V940) combined with pembrolizumab significantly reduced the risk of recurrence in high-risk melanoma (NCT03897881) and received FDA Breakthrough Therapy designation (176). This landmark result validates that combining neoantigen vaccines with immune checkpoint inhibitors (ICIs) can yield clinically meaningful survival benefits, providing conceptual support for the future inclusion of Mtp53-derived neoantigens. Although these vaccines do not directly target Mtp53, they validate the feasibility of harnessing neoantigens to activate anti-tumor immunity. Incorporating Mtp53-derived neoantigens into future personalized vaccine designs may enhance immunogenicity, though this remains at a conceptual stage. Notably, the phase Ib trial of APR-246 combined with pembrolizumab (NCT04383938) reported a manageable safety profile, with grade ≥3 treatment-related adverse events occurring in 28% of patients and immune-related adverse events (irAEs) in 12% (including colitis and pneumonitis), with no new safety signals observed (156). This irAE incidence is comparable to historical data for pembrolizumab monotherapy in similar populations (∼10–15%), suggesting that APR-246 may not substantially exacerbate the immune toxicity of immune checkpoint inhibitors (ICIs). Pharmacokinetic/pharmacodynamic (PK/PD) analyses further revealed that APR-246 exposure levels correlated positively with CD8^+^ T-cell activation but showed no significant association with irAEs, implying a potentially dissociable efficacy–toxicity window. However, the study was limited by a small sample size (n=34) and did not specifically assess high-risk irAEs (e.g., myocarditis); thus, the safety of this combination warrants further evaluation in larger, biomarker-enriched trials.

#### 6.2.3 Targeting metabolic and epigenetic microenvironments

Mtp53 reprograms metabolism (e.g., enhancing glycolysis, lactate accumulation) and recruits epigenetic regulators (e.g., EZH2, BET proteins) to shape an immunosuppressive TME. Preclinical studies indicate that inhibition of monocarboxylate transporter 4 (MCT4) can block lactate efflux, while GLUT1 inhibitors (e.g., BAY-876 (177)) can attenuate Mtp53-driven glycolysis, thereby alleviating TME acidification and partially reversing lactate-mediated suppression of T-cell function. Similarly, EZH2 or BET inhibitors have demonstrated potential *in vitro* and in animal models to reverse Mtp53-induced chromatin silencing and re-activate immune-related genes such as *CXCL10* and interferon-responsive genes. Additionally, targeting stromal cells—such as CAFs or TAMs—to disrupt their crosstalk with Mtp53-expressing tumor cells is also being explored as a strategy to overcome immune resistance.

However, no metabolic–immune or epigenetic–immune combination strategies specifically tailored for *TP53*-mutant tumors have yet advanced to confirmatory clinical validation. Although early-phase trials of MCT1 inhibitors (e.g., AZD3965) and EZH2/BET inhibitors are underway, none of these studies have used *TP53* mutation status as an enrollment criterion, nor have they systematically evaluated the impact of these agents on the immune microenvironment or their ability to modulate the efficacy of ICIs (178, 179). GLUT1 inhibitors such as BAY-876 remain in preclinical development and have not yet entered human trials.

Moreover, combination strategies pairing Mtp53 reactivators (e.g., APR-246) with metabolic inhibitors (e.g., GLUT1 or MCT inhibitors) currently lack supporting safety data. Given that agents like BAY-876 have not been tested in humans, the potential combined toxicities—such as energy deprivation in high-glucose-demand organs or suppression of immune cell metabolism—are entirely unknown. To date, no preclinical toxicology studies have been reported that assess the safety or therapeutic window of such dual combinations. In the absence of fundamental toxicological data, clinical development of these regimens carries substantial risk and urgently requires systematic evaluation in humanized models to determine the maximum tolerated dose, metabolic compensatory mechanisms, and dual effects on the immune microenvironment.

Current support for these approaches stems largely from mechanistic studies and animal models; there is a critical lack of clinical data evaluating antitumor or immune endpoints (e.g., ORR, PFS, changes in T-cell infiltration) specifically in *TP53*-mutant populations. Thus, while “microenvironment reprogramming” holds theoretical promise for creating a more favorable ecosystem to enhance the efficacy of other therapies—such as ICIs or targeted agents—its clinical translation remains highly exploratory. Prospective trial designs stratified by *TP53* mutation status are urgently needed to validate this concept.

Despite significant progress in Mtp53-targeted therapy, most strategies remain in the “promising but unproven” stage. The phase III failure of APR-246 serves as a cautionary tale: biological plausibility does not guarantee clinical efficacy. Future breakthroughs may depend on biomarker-driven patient stratification, individualized combination strategies integrating mutation subtypes and microenvironmental features, and dynamic monitoring of clonal evolution and resistance mechanisms. Together, these approaches represent the most viable path toward translating Mtp53 targeting into meaningful clinical benefits.

## 7 Conclusions and future perspectives

Mtp53 is not merely a loss-of-function variant but an active “systemic rewirer” that drives tumor progression and immune evasion through GOF activities. This review has outlined how Mtp53 establishes a highly coordinated oncogenic network via the metabolism–epigenetics–immunity axis. From aberrant LLPS leading to pathological nuclear condensates, to metabolic rewiring—lactate accumulation, α-KG imbalance, and acetyl-CoA enrichment—acting as epigenetic messengers that reshape chromatin landscapes, and further to multilayered immunosuppression including T-cell exhaustion, NK cell inhibition, TAM/M2 polarization, and CAF activation, Mtp53 orchestrates a pro-tumorigenic ecosystem through both cell-autonomous and non-autonomous mechanisms. This multidimensional regulatory network explains its strong association with poor prognosis and resistance to immunotherapy, underscoring Mtp53’s role as a central signaling hub in cancer.

However, Mtp53 function exhibits marked mutation dependency, tissue specificity, and dynamic evolution, resulting in heterogeneous—sometimes contradictory—effects on the TIME. For instance, TP53/KRAS co-mutated lung cancers may respond to ICB, whereas TP53 mutations in triple-negative breast or colorectal cancers are often linked to “cold” tumor phenotypes. Notably, in specific genomic contexts such as TP53/KRAS co-mutated NSCLC, Mtp53 is associated with high tumor mutational burden and neoantigen load and paradoxically correlates with enhanced immune infiltration and improved response to immune checkpoint blockade (ICB), reflecting its potential immunogenic role. This context dependence challenges the utility of TP53 mutation status as a standalone biomarker and underscores the need to move beyond binary “mutant *vs*. wild-type” classification toward a multidimensional, spatiotemporally resolved understanding of Mtp53 biology.

Future breakthroughs will require synergistic advances across several fronts. First, targeting the biophysical behavior of Mtp53—particularly its enhanced LLPS capacity—could open a new therapeutic paradigm. Pathological nuclear condensates formed by Mtp53 can sequester transcriptional co-activators (e.g., BRD4, Mediator), driving immunosuppressive gene programs. Developing small molecules, peptide mimetics, or molecular glues that selectively disrupt these condensates, combined with cryo-EM structural insights, single-molecule imaging, and AI-driven drug design, may enable precise reversal of oncogenic transcription.

Second, building integrative biomarker platforms is essential for precision targeting. Genomic profiling alone is insufficient. A dynamic framework integrating mutation conformation classes (e.g., R175H, Y220C), single-cell and spatial multi-omics of the TIME, and ctDNA-based monitoring of clonal evolution is urgently needed. Such platforms could enable patient stratification and real-time detection of adaptive resistance, guiding optimal timing and selection of combination therapies.

Third, mutation-informed combination strategies are critical. Given the complexity and redundancy of the Mtp53 network, monotherapy is unlikely to succeed. Ideal regimens may combine Mtp53 reactivators (e.g., APR-246, PC14586) or degraders (e.g., HDAC6 or MVA pathway inhibitors) with ICB to restore T-cell responses; pair metabolic modulators (e.g., MCT4/GLUT1 inhibitors) or epigenetic drugs (e.g., EZH2/BET inhibitors) to reverse immune gene silencing; and explore Mtp53 neoantigen vaccines or adoptive cell therapies to turn Mtp53 into an immunogenic target. However, the phase III failure of APR-246 in MDS (NCT03745716) was likely due to a lack of stratification by mutation subtype—such as the inclusion of patients with nonsense or frameshift mutations—highlighting the need for future trials to prospectively enrich for populations with conformationally sensitive mutations. Meanwhile, although combinations of Mtp53-targeted agents with metabolic inhibitors or immune checkpoint inhibitors (ICIs) hold therapeutic promise, their cumulative toxicities (e.g., energy deprivation–related organ damage or immune-related adverse events [irAEs]) have not been systematically evaluated in preclinical models. It is therefore urgent to define the therapeutic window and maximum tolerated dose in humanized models to ensure that efficacy gains are not offset by unmanageable toxicity.

Finally, distal regulation and niche modulation warrant deeper exploration. Mtp53 communicates with stromal cells (CAFs), myeloid populations, and even the gut microbiota via exosomes, cytokines, and metabolites. These non-tumor components may feed back to stabilize Mtp53 or amplify its GOF. Targeting the tumor niche—through stromal reprogramming, microbiome modulation, or dietary interventions—could enhance therapeutic efficacy and delay resistance.

In summary, as a central integrator of metabolism, epigenetics, and immunity, Mtp53 can drive profound immunosuppression while also exhibiting immunogenic potential under specific conditions; the success of its clinical translation depends not only on mechanistic innovation but also critically on precise patient stratification and rigorous safety validation of combination regimens. Disrupting this network demands a systems-level strategy—multipronged, multilayered, and dynamically adaptive. Integrating phase separation biology, spatial omics, AI-powered drug prediction, ctDNA monitoring, and microbiome science into precision oncology will not only deepen our understanding of p53 biology but also unlock transformative therapeutic opportunities for Mtp53-driven cancers. The path forward lies in bridging the gap between mechanistic insight and clinical translation, advancing the field from “theoretically feasible” to “clinically accessible,” and ultimately achieving precise, durable control of Mtp53-driven malignancies.

## Glossary

5-FU: 5-Fluorouracil
ACAT1: Acyl-CoA:cholesterol acyltransferase 1
ACC: Acetyl-CoA carboxylase
ACP6: Lysophosphatidic acid phosphatase type 6
ACSS2: Acetyl-CoA synthetase short-chain family member 2
α-SMA: α-Smooth muscle actin
AML: Acute myeloid leukemia
ARG1: Arginase 1
BRD4: Bromodomain-containing protein 4
CAFs: Cancer-associated fibroblasts
ChIP-PS: Coupling chromatin immunoprecipitation followed by proximity ligation
circRNAs: circular RNAs
CR: Complete response rates
CRPC: Castration-resistant prostate cancer
CSF-1: Colony-stimulating factor 1
CTLs: Cytotoxic T lymphocytes
CTLA-4: Cytotoxic T-lymphocyte-associated protein 4
CX3CL1: CX3C motif chemokine ligand 1
CXCL12: C-X-C motif chemokine ligand 12
DBD: DNA-binding domain
DCs: Dendritic cells
DNE: Dominant-negative effect
DNAM-1: DNAX accessory molecule-1
EMT: Epithelial–mesenchymal transition
ENO1: Enolase 1
ERVs: Endogenous retroviruses
EMA: European Medicines Agency
FASN: Fatty acid synthase
FDA: U.S. Food and Drug Administration
FFPE: Formalin-fixed paraffin-embedded
FGF10: Fibroblast growth factor 10
FRAP: Fluorescence recovery after photobleaching
G-CSF: Granulocyte colony-stimulating factor
GGPP: Geranylgeranyl pyrophosphate
GLUT1: Glucose transporter 1
GLUT5: Glucose transporter 5
GM-CSF: Granulocyte-macrophage colony-stimulating factor
GOF: Gain of function
GPR81: G-protein-coupled receptor 81
HATs: Histone acetyltransferases
H3K18la: H3K18 lactylation
H3K27ac: H3 lysine 27 acetylation
H3K4me1: H3K4 monomethylation
H3K4me2: H3K4 dimethylation
HDAC: Histone deacetylase
HGFR: Hepatocyte growth factor receptor
HIF-1α: Hypoxia-inducible factor 1-alpha
HK-I: Hexokinase-I
HNSCC: Head and neck squamous cell carcinoma
HSP70: Heat shock protein 70
ICB: Immune checkpoint blockade
IDR: Intrinsically disordered region
IFN-β: Interferon-beta
IFNγ: Interferon-gamma
IL-6: Interleukin-6
iNOS: Inducible nitric oxide synthase
irAEs: Immune-related adverse events
KDM1B: Lysine-specific demethylase 1B
KHK: Ketohexokinase
Kla: Histone lysine lactylation
KLF5: Krüppel-like factor 5
LLPS: Liquid-liquid phase separation
lncRNAs: long non-coding RNAs
LOF: Loss of function
LOH: Loss of heterozygosity
LPA: Lysophosphatidic acid
LSD2: Histone demethylase lysine-specific demethylase 2
M2-TAMs: M2-polarized tumor-associated macrophages
M-CSF: Macrophage colony-stimulating factor
MDSCs: Myeloid-derived suppressor cells
MDS: Myelodysplastic syndromes
MEF2C: Myocyte enhancer factor 2C
MGST3: Microsomal glutathione S-transferase 3
MHC: Major histocompatibility complex
miRNAs: microRNAs
MLL4: Mixed-lineage leukemia 4
MnSOD: Manganese superoxide dismutase
MOMP: Mitochondrial outer membrane permeabilization
MRD: Minimal residual disease
MVA: Mevalonate
MUFA: Monounsaturated fatty acid
ncRNA: Non-coding RNA
NF-κB: Nuclear factor-kappa B
NF-Y: Nuclear transcription factor Y
NGS: Next-generation sequencing
NK: Natural killer
NO: Nitric oxide
NSCLC: Non-small cell lung cancer
NRF2: Nuclear factor erythroid 2-related factor 2
ORR: Overall response rates
OXPHOS: Oxidative phosphorylation
PCK2: Phosphoenolpyruvate carboxykinase 2
PDAC: Pancreatic ductal adenocarcinoma
PD-L1: Programmed death-ligand 1
PD/PK: Pharmacodynamic/Pharmacokinetic
PGC-1α: Peroxisome proliferator-activated receptor gamma coactivator 1-alpha
PKM2: Pyruvate kinase M2
PLA2G16: Phospholipase A2 group XVI
PMN-MDSCs: Polymorphonuclear myeloid-derived suppressor cells
PNETs: Pancreatic neuroendocrine tumors
PFS: Progression-free survival
PFKP: Phosphofructokinase-platelet
PGC-1α: Peroxisome proliferator-activated receptor gamma coactivator 1-alpha
PTCs: Premature termination codons
RAE-1: Early transcript 1
ROS: Reactive oxygen species
SCLC: Small cell lung cancer
SCO2: Synthesis of cytochrome c oxidase 2
SESN1: Sestrin 1
SESN2: Sestrin 2
SGOC: Serine–glycine–one-carbon
SLC2A5: Solute carrier family 2 member 5
SLC45A4: Solute carrier family 45 member 4
SLC7A11: Solute carrier family 7 member 11
SOAT1: Sterol O-acyltransferase 1
SDF-1: Stromal cell-derived factor-1
STAT3: Signal transducer and activator of transcription 3
SV40: Simian virus 40
TAMs: Tumor-associated macrophages
TCA: Tricarboxylic acid
TGF-β: Transforming growth factor-beta
TIME: Tumor immune microenvironment
TILs: Tumor-infiltrating lymphocytes
TMB: Tumor mutational burden
TNBC: Triple negative breast cancer
Tregs: Regulatory T cells
TXN: Thioredoxin
UCHL3: Ubiquitin C-terminal hydrolase L3
ULBP1: UL16-binding protein 1
ULBP2: UL16-binding protein 2
VAF: Variant allele frequency
VEGF: Vascular endothelial growth factor
Wtp53: Wild type p53

## References

[1] Lane DP Crawford LV . T antigen is bound to a host protein in SV40-transformed cells. Nature. (1979) 278:261–3. doi: 10.1038/278261a0, PMID: 218111

[2] Carlsen L Zhang S Tian X De La Cruz A George A Arnoff TE . The role of p53 in anti-tumor immunity and response to immunotherapy. Front Mol Biosci. (2023) 10:1148389. doi: 10.3389/fmolb.2023.1148389, PMID: 37602328 PMC10434531

[3] Binnewies M Roberts EW Kersten K Chan V Fearon DF Merad M . Understanding the tumor immune microenvironment (TIME) for effective therapy. Nat Med. (2018) 24:541–50. doi: 10.1038/s41591-018-0014-x, PMID: 29686425 PMC5998822

[4] Sharma P Hu-Lieskovan S Wargo JA Ribas A . Primary, adaptive, and acquired resistance to cancer immunotherapy. Cell. (2017) 168:707–23. doi: 10.1016/j.cell.2017.01.017, PMID: 28187290 PMC5391692

[5] Chen DS Mellman I . Elements of cancer immunity and the cancer-immune set point. Nature. (2017) 541:321–30. doi: 10.1038/nature21349, PMID: 28102259

[6] Boyle ST Johan MZ Samuel MS . Tumour-directed microenvironment remodelling at a glance. J Cell Sci. (2020) 133:jcs247783. doi: 10.1242/jcs.247783, PMID: 33443095

[7] Chen X Zhang T Su W Dou Z Zhao D Jin X . Mutant p53 in cancer: from molecular mechanism to therapeutic modulation. Cell Death Dis. (2022) 13:974. doi: 10.1038/s41419-022-05408-1, PMID: 36400749 PMC9674619

[8] Hsiue EH Wright KM Douglass J Hwang MS Mog BJ Pearlman AH . Targeting a neoantigen derived from a common TP53 mutation. Science. (2021) 371:eabc8697. doi: 10.1126/science.abc8697, PMID: 33649166 PMC8208645

[9] Ishak CA Marhon SA Tchrakian N Hodgson A Loo Yau H Gonzaga IM . Chronic viral mimicry induction following p53 loss promotes immune evasion. Cancer Discov. (2025) 15:793–817. doi: 10.1158/2159-8290.CD-24-0094, PMID: 39776167 PMC12776606

[10] Li J Zhang S Wang B Dai Y Wu J Liu D . Pharmacological rescue of mutant p53 triggers spontaneous tumor regression via immune responses. Cell Rep Med. (2025) 6:101976. doi: 10.1016/j.xcrm.2025.101976, PMID: 39986271 PMC11970324

[11] Petronilho EC Pedrote MM Marques MA Passos YM Mota MF Jakobus B . Phase separation of p53 precedes aggregation and is affected by oncogenic mutations and ligands. Chem Sci. (2021) 12:7334–49. doi: 10.1039/d1sc01739j, PMID: 34163823 PMC8171334

[12] Bischoff P Reck M Overbeck T Christopoulos P Rittmeyer A Lüders H . Outcome of first-line treatment with pembrolizumab according to KRAS/TP53 mutational status for nonsquamous programmed death-ligand 1-high (≥50%) NSCLC in the German National Network Genomic Medicine Lung Cancer. J Thorac Oncol. (2024) 19:803–17. doi: 10.1016/j.jtho.2023.12.015, PMID: 38096950

[13] Liu N Jiang X Guo L Zhang C Jiang M Sun Z . Mutant p53 achieved gain-of-function by promoting tumor growth and immune escape through PHLPP2/AKT/PD-L1 pathway. Int J Biol Sci. (2022) 18:2419–38. doi: 10.7150/ijbs.67200, PMID: 35414774 PMC8990467

[14] Kim JY Park K Jung HH Lee E Cho EY Lee KH . Association between mutation and expression of TP53 as a potential prognostic marker of triple-negative breast cancer. Cancer Res Treat. (2016) 48:1338–50. doi: 10.4143/crt.2015.430, PMID: 26910472 PMC5080805

[15] Wang H Guo M Wei H Chen Y . Targeting p53 pathways: mechanisms, structures, and advances in therapy. Signal Transduction Targeted Ther. (2023) 8:92. doi: 10.1038/s41392-023-01347-1, PMID: 36859359 PMC9977964

[16] Degtjarik O Golovenko D Diskin-Posner Y Abrahmsén L Rozenberg H Shakked Z . Structural basis of reactivation of oncogenic p53 mutants by a small molecule: methylene quinuclidinone (MQ). Nat Commun. (2021) 12:7057. doi: 10.1038/s41467-021-27142-6, PMID: 34862374 PMC8642532

[17] Wang J Hu Y Escamilla-Rivera V Gonzalez CL Tang L Wang B . Epithelial mutant p53 promotes resistance to anti-PD-1-mediated oral cancer immunoprevention in carcinogen-induced mouse models. Cancers. (2021) 13:1471. doi: 10.3390/cancers13061471, PMID: 33806894 PMC8005156

[18] Lang GA Iwakuma T Suh YA Liu G Rao VA Parant JM . Gain of function of a p53 hot spot mutation in a mouse model of Li-Fraumeni syndrome. Cell. (2004) 119:861–72. doi: 10.1016/j.cell.2004.11.006, PMID: 15607981

[19] Marques MA de Oliveira GAP Silva JL . The chameleonic behavior of p53 in health and disease: the transition from a client to an aberrant condensate scaffold in cancer. Essays Biochem. (2022) 66:1023–33. doi: 10.1042/EBC20220064, PMID: 36350030

[20] Petronilho EC de Andrade GC de Sousa GDS Almeida FP Mota MF Gomes AVDS . Oncogenic p53 triggers amyloid aggregation of p63 and p73 liquid droplets. Commun Chem. (2024) 7:207. doi: 10.1038/s42004-024-01289-x, PMID: 39284933 PMC11405828

[21] Mahat DB Kumra H Castro SA Metcalf E Nguyen K Morisue R . Mutant p53 exploits enhancers to elevate immunosuppressive chemokine expression and impair immune checkpoint inhibitors in pancreatic cancer. Immunity. (2025) 58:1688–1705.e9. doi: 10.1016/j.immuni.2025.06.005, PMID: 40592342 PMC12233199

[22] Zong Z Xie F Wang S Wu X Zhang Z Yang B . Alanyl-tRNA synthetase, AARS1, is a lactate sensor and lactyltransferase that lactylates p53 and contributes to tumorigenesis. Cell. (2024) 187:2375–2392.e33. doi: 10.1016/j.cell.2024.04.002, PMID: 38653238

[23] Yu Y Liu Q Zeng J Tan Y Tang Y Wei G . Multiscale simulations reveal the driving forces of p53C phase separation accelerated by oncogenic mutations. Chem Sci. (2024) 15:12806–18. doi: 10.1039/d4sc03645j, PMID: 39148776 PMC11323318

[24] Boija A Klein IA Sabari BR Dall'Agnese A Coffey EL Zamudio AV . Transcription factors activate genes through the phase-separation capacity of their activation domains. Cell. (2018) 175:1842–1855.e16. doi: 10.1016/j.cell.2018.10.042, PMID: 30449618 PMC6295254

[25] Mantovani A Allavena P Sica A Balkwill F . Cancer-related inflammation. Nature. (2008) 454:436–44. doi: 10.1038/nature07205, PMID: 18650914

[26] Wu J Yang Q Zhu Y Xia T Yi L Wang J . DNAJA1 promotes proliferation and metastasis of breast cancer by activating mutant P53/NF-κB pathway. Pathol - Res Pract. (2023) 252:154921. doi: 10.1016/j.prp.2023.154921, PMID: 37977037

[27] Lin Y Mallen-St Clair J Luo J Sharma S Dubinett S St John M . p53 modulates NF-κB mediated epithelial-to-mesenchymal transition in head and neck squamous cell carcinoma. Oral Oncol. (2015) 51:921–8. doi: 10.1016/j.oraloncology.2015.07.006, PMID: 26306422

[28] Rajagopalan A Feng Y Gayatri MB Ranheim EA Klungness T Matson DR . A gain-of-function p53 mutant synergizes with oncogenic NRAS to promote acute myeloid leukemia in mice. J Clin Invest. (2023) 133:e173116. doi: 10.1172/JCI173116, PMID: 37847561 PMC10721149

[29] Schwitalla S Ziegler PK Horst D Becker V Kerle I Begus-Nahrmann Y . Loss of p53 in enterocytes generates an inflammatory microenvironment enabling invasion and lymph node metastasis of carcinogen-induced colorectal tumors. Cancer Cell. (2013) 23:93–106. doi: 10.1016/j.ccr.2012.11.014, PMID: 23273920

[30] Wellenstein MD Coffelt SB Duits DEM van Miltenburg MH Slagter M de Rink I . Loss of p53 triggers WNT-dependent systemic inflammation to drive breast cancer metastasis. Nature. (2019) 572:538–42. doi: 10.1038/s41586-019-1450-6, PMID: 31367040 PMC6707815

[31] Rodriguez-Meira A Norfo R Wen S Chédeville AL Rahman H O'Sullivan J . Single-cell multi-omics identifies chronic inflammation as a driver of TP53-mutant leukemic evolution. Nat Genet. (2023) 55:1531–41. doi: 10.1038/s41588-023-01480-1, PMID: 37666991 PMC10484789

[32] Dong X Li C Deng C Liu J Li D Zhou T . Regulated secretion of mutant p53 negatively affects T lymphocytes in the tumor microenvironment. Oncogene. (2024) 43:92–105. doi: 10.1038/s41388-023-02886-1, PMID: 37952080 PMC10774126

[33] Blagih J Zani F Chakravarty P Hennequart M Pilley S Hobor S . Cancer-specific loss of p53 leads to a modulation of myeloid and T cell responses. Cell Rep. (2020) 30:481–496.e6. doi: 10.1016/j.celrep.2019.12.028, PMID: 31940491 PMC6963783

[34] Li L Muftuoglu M Ayoub E Lv J Basyal M Bidikian A . Somatic TP53 mutations drive T and NK cell dysfunction in AML and can be rescued by reactivating wild type p53. medRxiv. (2025). doi: 10.1101/2025.04.11.25325281, PMID: 40297420 PMC12036377

[35] Du Y Fan Z Li L Xue Y Zhao S . Kasumi-1 exosome plays a major T-cell immune evasion role in TP53-type acute leukemia. Immunobiology. (2025) 230:153102. doi: 10.1016/j.imbio.2025.153102, PMID: 40682963

[36] Jin K Xu J Su X Xu Z Li B Liu G . TP53 disruptive mutation predicts platinum-based chemotherapy and PD-1/PD-L1 blockade response in urothelial carcinoma. J Pathol. (2024) 263:139–49. doi: 10.1002/path.6266, PMID: 38380548

[37] Textor S Fiegler N Arnold A Porgador A Hofmann TG Cerwenka A . Human NK cells are alerted to induction of p53 in cancer cells by upregulation of the NKG2D ligands ULBP1 and ULBP2. Cancer Res. (2011) 71:5998–6009. doi: 10.1158/0008-5472.CAN-10-3211, PMID: 21764762

[38] Uddin MB Roy KR Hill RA Roy SC Gu X Li L . p53 missense mutant G242A subverts natural killer cells in sheltering mouse breast cancer cells against immune rejection. Exp Cell Res. (2022) 417:113210. doi: 10.1016/j.yexcr.2022.113210, PMID: 35597298

[39] Veneziani I Infante P Ferretti E Melaiu O Battistelli C Lucarini V . Nutlin-3a enhances natural killer cell-mediated killing of neuroblastoma by restoring p53-dependent expression of ligands for NKG2D and DNAM-1 receptors. Cancer Immunol Res. (2021) 9:170–83. doi: 10.1158/2326-6066.CIR-20-0313, PMID: 33303573

[40] Pan R Ryan J Pan D Wucherpfennig KW Letai A . Augmenting NK cell-based immunotherapy by targeting mitochondrial apoptosis. Cell. (2022) 185:1521–1538.e18. doi: 10.1016/j.cell.2022.03.030, PMID: 35447071 PMC9097966

[41] Chipuk JE Kuwana T Bouchier-Hayes L Droin NM Newmeyer DD Schuler M . Direct activation of Bax by p53 mediates mitochondrial membrane permeabilization and apoptosis. Science. (2004) 303:1010–4. doi: 10.1126/science.1092734, PMID: 14963330

[42] Chollat-Namy M Ben Safta-Saadoun T Haferssas D Meurice G Chouaib S Thiery J . The pharmacological reactivation of p53 function improves breast tumor cell lysis by granzyme B and NK cells through induction of autophagy. Cell Death Dis. (2019) 10:695. doi: 10.1038/s41419-019-1950-1, PMID: 31541080 PMC6754511

[43] Siolas D Vucic E Kurz E Hajdu C Bar-Sagi D . Gain-of-function p53R172H mutation drives accumulation of neutrophils in pancreatic tumors, promoting resistance to immunotherapy. Cell Rep. (2021) 36:109578. doi: 10.1016/j.celrep.2021.109578, PMID: 34433022 PMC8687588

[44] Wörmann SM Song L Ai J Diakopoulos KN Kurkowski MU Görgülü K . Loss of P53 function activates JAK2-STAT3 signaling to promote pancreatic tumor growth, stroma modification, and gemcitabine resistance in mice and is associated with patient survival. Gastroenterology. (2016) 151:180–193.e12. doi: 10.1053/j.gastro.2016.03.010, PMID: 27003603

[45] Nian Z Dou Y Shen Y Liu J Du X Jiang Y . Interleukin-34-orchestrated tumor-associated macrophage reprogramming is required for tumor immune escape driven by p53 inactivation. Immunity. (2024) 57:2344–2361.e7. doi: 10.1016/j.immuni.2024.08.015, PMID: 39321806

[46] Efe G Dunbar KJ Sugiura K Cunningham K Carcamo S Karaiskos S . p53 gain-of-function mutation induces metastasis via BRD4-dependent CSF-1 expression. Cancer Discov. (2023) 13:2632–51. doi: 10.1158/2159-8290.CD-23-0601, PMID: 37676642 PMC10841313

[47] Cooks T Pateras IS Jenkins LM Patel KM Robles AI Morris J . Mutant p53 cancers reprogram macrophages to tumor supporting macrophages via exosomal miR-1246. Nat Commun. (2018) 9:771. doi: 10.1038/s41467-018-03224-w, PMID: 29472616 PMC5823939

[48] Nuñez SY Ziblat A Secchiari F Torres NI Sierra JM Raffo Iraolagoitia XL . Human M2 macrophages limit NK cell effector functions through secretion of TGF-β and engagement of CD85j. J Immunol. (2018) 200:1008–15. doi: 10.4049/jimmunol.1700737, PMID: 29282306

[49] Liu Q Yu B Tian Y Dan J Luo Y Wu X . P53 mutant p53N236S regulates cancer-associated fibroblasts properties through Stat3 pathway. OncoTargets Ther. (2020) 13:1355–63. doi: 10.2147/OTT.S229065, PMID: 32104002 PMC7027832

[50] Ogawa T Kikuchi S Tabuchi M Mitsui E Une Y Tazawa H . Modulation of p53 expression in cancer-associated fibroblasts prevents peritoneal metastasis of gastric cancer. Mol Ther - Oncolytics. (2022) 25:249–61. doi: 10.1016/j.omto.2022.04.009, PMID: 35615263 PMC9108396

[51] Saiki H Hayashi Y Yoshii S Kimura E Nakagawa K Kato M . The apelin-apelin receptor signaling pathway in fibroblasts is involved in tumor growth via p53 expression of cancer cells. Int J Oncol. (2023) 63:139. doi: 10.3892/ijo.2023.5587, PMID: 37921070 PMC10631769

[52] Addadi Y Moskovits N Granot D Lozano G Carmi Y Apte RN . p53 status in stromal fibroblasts modulates tumor growth in an SDF1-dependent manner. Cancer Res. (2010) 70:9650–8. doi: 10.1158/0008-5472.CAN-10-1146, PMID: 20952507 PMC2999653

[53] Madar S Harel E Goldstein I Stein Y Kogan-Sakin I Kamer I . Mutant p53 attenuates the anti-tumorigenic activity of fibroblasts-secreted interferon beta. PloS One. (2013) 8:e61353. doi: 10.1371/journal.pone.0061353, PMID: 23630584 PMC3632588

[54] Agarwal H Tal P Goldfinger N Chattopadhyay E Malkin D Rotter V . Mutant p53 reactivation restricts the protumorigenic consequences of wild type p53 loss of heterozygosity in Li-Fraumeni syndrome patient-derived fibroblasts. Cell Death Differentiation. (2024) 31:855–67. doi: 10.1038/s41418-024-01307-4, PMID: 38745079 PMC11239894

[55] Xin H Zhao Z Guo S Tian R Ma L Yang Y . Targeting the JAK2-STAT3-UCHL3-ENO1 axis suppresses glycolysis and enhances the sensitivity to 5-FU chemotherapy in TP53-mutant colorectal cancer. Acta Pharm Sin B. (2025) 15:2529–44. doi: 10.1016/j.apsb.2025.03.041, PMID: 40487654 PMC12145010

[56] Li HJ Ke FY Lin CC Lu MY Kuo YH Wang YP . ENO1 promotes lung cancer metastasis via HGFR and WNT signaling-driven epithelial-to-mesenchymal transition. Cancer Res. (2021) 81:4094–109. doi: 10.1158/0008-5472.CAN-20-3543, PMID: 34145039

[57] Xia W Bai H Deng Y Yang Y . PLA2G16 is a mutant p53/KLF5 transcriptional target and promotes glycolysis of pancreatic cancer. J Cell Mol Med. (2020) 24:12642–55. doi: 10.1111/jcmm.15832, PMID: 32985124 PMC7686977

[58] Chen W Huang F Huang J Li Y Peng J Zhuang Y . SLC45A4 promotes glycolysis and prevents AMPK/ULK1-induced autophagy in TP53 mutant pancreatic ductal adenocarcinoma. J Gene Med. (2021) 23:e3364. doi: 10.1002/jgm.3364, PMID: 34010493 PMC8459293

[59] Fang JH Chen JY Zheng JL Zeng HX Chen JG Wu CH . Fructose metabolism in tumor endothelial cells promotes angiogenesis by activating AMPK signaling and mitochondrial respiration. Cancer Res. (2023) 83:1249–63. doi: 10.1158/0008-5472.CAN-22-1844, PMID: 36715635

[60] Hao ZN Tan XP Zhang Q Li J Xia R Ma Z . Lactate and lactylation: Dual regulators of T-cell-mediated tumor immunity and immunotherapy. Biomolecules. (2024) 14:1646. doi: 10.3390/biom14121646, PMID: 39766353 PMC11674224

[61] San-Millán I Julian CG Matarazzo C Martinez J Brooks GA . Is lactate an oncometabolite? Evidence supporting a role for lactate in the regulation of transcriptional activity of cancer-related genes in MCF7 breast cancer cells. Front Oncol. (2020) 10:1536. doi: 10.3389/fonc.2019.01536, PMID: 32010625 PMC6971189

[62] Ding R Yu X Hu Z Dong Y Huang H Zhang Y . Lactate modulates RNA splicing to promote CTLA-4 expression in tumor-infiltrating regulatory T cells. Immunity. (2024) 57:528–540.e6. doi: 10.1016/j.immuni.2024.01.019, PMID: 38417442

[63] Guo S Zhou J Lou P Weng L Ye X Guo J . Potentiated effects of lactate receptor GPR81 on immune microenvironment in breast cancer. Mol Carcinogenesis. (2023) 62:1369–77. doi: 10.1002/mc.23582, PMID: 37249360

[64] Brand A Singer K Koehl GE Kolitzus M Schoenhammer G Thiel A . LDHA-associated lactic acid production blunts tumor immunosurveillance by T and NK cells. Cell Metab. (2016) 24:657–71. doi: 10.1016/j.cmet.2016.08.011, PMID: 27641098

[65] Caronni N Simoncello F Stafetta F Guarnaccia C Ruiz-Moreno JS Opitz B . Downregulation of membrane trafficking proteins and lactate conditioning determine loss of dendritic cell function in lung cancer. Cancer Res. (2018) 78:1685–99. doi: 10.1158/0008-5472.CAN-17-1307, PMID: 29363545

[66] Manoharan I Prasad PD Thangaraju M Manicassamy S . Lactate-dependent regulation of immune responses by dendritic cells and macrophages. Front Immunol. (2021) 12:691134. doi: 10.3389/fimmu.2021.691134, PMID: 34394085 PMC8358770

[67] Gan X Zhang R Gu J Ju Z Wu X Wang Q . Acidic microenvironment regulates the severity of hepatic ischemia/reperfusion injury by modulating the generation and function of Tregs via the PI3K-mTOR pathway. Front Immunol. (2020) 10:2945. doi: 10.3389/fimmu.2019.02945, PMID: 31998287 PMC6962105

[68] Su J Mao X Wang L Chen Z Wang W Zhao C . Lactate/GPR81 recruits regulatory T cells by modulating CX3CL1 to promote immune resistance in a highly glycolytic gastric cancer. OncoImmunology. (2024) 13:2320951. doi: 10.1080/2162402X.2024.2320951, PMID: 38419759 PMC10900271

[69] Yang X Lu Y Hang J Zhang J Zhang T Huo Y . Lactate-modulated immunosuppression of myeloid-derived suppressor cells contributes to the radioresistance of pancreatic cancer. Cancer Immunol Res. (2020) 8:1440–51. doi: 10.1158/2326-6066.CIR-20-0111, PMID: 32917658

[70] Mu X Shi W Xu Y Xu C Zhao T Geng B . Tumor-derived lactate induces M2 macrophage polarization via the activation of the ERK/STAT3 signaling pathway in breast cancer. Cell Cycle. (2018) 17:428–38. doi: 10.1080/15384101.2018.1444305, PMID: 29468929 PMC5927648

[71] Chen P Zuo H Xiong H Kolar MJ Chu Q Saghatelian A . Gpr132 sensing of lactate mediates tumor-macrophage interplay to promote breast cancer metastasis. Proc Natl Acad Sci USA. (2017) 114:580–5. doi: 10.1073/pnas.1614035114, PMID: 28049847 PMC5255630

[72] Di Agostino S Strano S Emiliozzi V Zerbini V Mottolese M Sacchi A . Gain of function of mutant p53: the mutant p53/NF-Y protein complex reveals an aberrant transcriptional mechanism of cell cycle regulation. Cancer Cell. (2006) 10:191–202. doi: 10.1016/j.ccr.2006.08.013, PMID: 16959611

[73] Wanka C Brucker DP Bähr O Ronellenfitsch M Weller M Steinbach JP . Synthesis of cytochrome C oxidase 2: a p53-dependent metabolic regulator that promotes respiratory function and protects glioma and colon cancer cells from hypoxia-induced cell death. Oncogene. (2012) 31:3764–76. doi: 10.1038/onc.2011.530, PMID: 22120717

[74] Hernández-Reséndiz I Gallardo-Pérez JC López-Macay A Robledo-Cadena DX García-Villa E Gariglio P . Mutant p53R248Q downregulates oxidative phosphorylation and upregulates glycolysis under normoxia and hypoxia in human cervix cancer cells. J Cell Physiol. (2019) 234:5524–36. doi: 10.1002/jcp.27354, PMID: 30272821

[75] Eriksson M Ambroise G Ouchida AT Lima Queiroz A Smith D Gimenez-Cassina A . Effect of mutant p53 proteins on glycolysis and mitochondrial metabolism. Mol Cell Biol. (2017) 37:e00328–17. doi: 10.1128/MCB.00328-17, PMID: 28993478 PMC5705820

[76] Basu S Gnanapradeepan K Barnoud T Kung CP Tavecchio M Scott J . Mutant p53 controls tumor metabolism and metastasis by regulating PGC-1α. Genes Dev. (2018) 32:230–43. doi: 10.1101/gad.309062.117, PMID: 29463573 PMC5859965

[77] Liu Y Gu W . The complexity of p53-mediated metabolic regulation in tumor suppression. Semin Cancer Biol. (2022) 85:4–32. doi: 10.1016/j.semcancer.2021.03.010, PMID: 33785447 PMC8473587

[78] Poles M Pacchiana R Mortali C Cisterna B Mullappilly N Celesia A . Mutant p53 affects the mitochondrial proteome, promoting mitochondrial fragmentation and OXPHOS in pancreatic ductal adenocarcinoma cells. FEBS J. (2025) 293:156–174. doi: 10.1111/febs.70223, PMID: 40819215

[79] Chao CH Wang CY Wang CH Chen TW Hsu HY Huang HW . Mutant p53 attenuates oxidative phosphorylation and facilitates cancer stemness through downregulating miR-200c-PCK2 axis in basal-like breast cancer. Mol Cancer Res. (2021) 19:1900–16. doi: 10.1158/1541-7786.MCR-21-0098, PMID: 34312289

[80] Martinez-Bernabe T Morla-Barcelo PM Fiore A Donadelli M Roca P Oliver J . Hotspot mutant p53-R273H enhances mitochondrial biogenesis and cell migration in primary colorectal cancer in response to oxaliplatin. Biochim Biophys Acta – Mol Cell Res. (2025) 1873:120073. doi: 10.1016/j.bbamcr.2025.120073, PMID: 41161608

[81] Torrens-Mas M Cordani M Mullappilly N Pacchiana R Riganti C Palmieri M . Mutant p53 induces SIRT3/MnSOD axis to moderate ROS production in melanoma cells. Arch Biochem Biophysics. (2020) 679:108219. doi: 10.1016/j.abb.2019.108219, PMID: 31812668

[82] Lisek K Campaner E Ciani Y Walerych D Del Sal G . Mutant p53 tunes the NRF2-dependent antioxidant response to support survival of cancer cells. Oncotarget. (2018) 9:20508–23. doi: 10.18632/oncotarget.24974, PMID: 29755668 PMC5945496

[83] Cordani M Butera G Pacchiana R Masetto F Mullappilly N Riganti C . Mutant p53-associated molecular mechanisms of ROS regulation in cancer cells. Biomolecules. (2020) 10:361. doi: 10.3390/biom10030361, PMID: 32111081 PMC7175157

[84] Dibra D Xiong S Moyer SM El-Naggar AK Qi Y Su X . Mutant p53 protects triple-negative breast adenocarcinomas from ferroptosis *in vivo*. Sci Adv. (2024) 10:eadk1835. doi: 10.1126/sciadv.adk1835, PMID: 38354236 PMC10866549

[85] Oni TE Biffi G Baker LA Hao Y Tonelli C Somerville TDD . SOAT1 promotes mevalonate pathway dependency in pancreatic cancer. J Exp Med. (2020) 217:e20192389. doi: 10.1084/jem.20192389, PMID: 32633781 PMC7478739

[86] Cotton K Comer C Caporali S Butera A Gurres S Capradossi F . Lipidome atlas of p53 mutant variants in pancreatic cancer. Biol Direct. (2025) 20:51. doi: 10.1186/s13062-025-00635-w, PMID: 40217553 PMC11992884

[87] Liu J Shen Y Liu J Xu D Chang CY Wang J . Lipogenic enzyme FASN promotes mutant p53 accumulation and gain-of-function through palmitoylation. Nat Commun. (2025) 16:1762. doi: 10.1038/s41467-025-57099-9, PMID: 39971971 PMC11839913

[88] Chryplewicz A Tienda SM Nahotko DA Peters PN Lengyel E Eckert MA . Mutant p53 regulates LPA signaling through lysophosphatidic acid phosphatase type 6. Sci Rep. (2019) 9:5195. doi: 10.1038/s41598-019-41352-5, PMID: 30914657 PMC6435808

[89] Xu H Xia H Zhou S Tang Q Bi F . Cholesterol activates the Wnt/PCP-YAP signaling in SOAT1-targeted treatment of colon cancer. Cell Death Discov. (2021) 7:38. doi: 10.1038/s41421-021-00243-5, PMID: 33637695 PMC7910478

[90] Tombari C Zannini A Bertolio R Pedretti S Audano M Triboli L . Mutant p53 sustains serine-glycine synthesis and essential amino acids intake promoting breast cancer growth. Nat Commun. (2023) 14:6777. doi: 10.1038/s41467-023-42458-1, PMID: 37880212 PMC10600207

[91] Tran TQ Lowman XH Reid MA Mendez-Dorantes C Pan M Yang Y . Tumor-associated mutant p53 promotes cancer cell survival upon glutamine deprivation through p21 induction. Oncogene. (2017) 36:1991–2001. doi: 10.1038/onc.2016.360, PMID: 27721412 PMC5383530

[92] Li G Wu J Li L Jiang P . p53 deficiency induces MTHFD2 transcription to promote cell proliferation and restrain DNA damage. Proc Natl Acad Sci USA. (2021) 118:e2019822118. doi: 10.1073/pnas.2019822118, PMID: 34244426 PMC8285905

[93] Yoo YA Quan S Yang W Guo Q Rodríguez Y Chalmers ZR . Asparagine dependency is a targetable metabolic vulnerability in TP53-altered castration-resistant prostate cancer. Cancer Res. (2024) 84:3004–22. doi: 10.1158/0008-5472.CAN-23-2910, PMID: 38959335 PMC11405136

[94] Hamada S Matsumoto R Tanaka Y Taguchi K Yamamoto M Masamune A . Nrf2 activation sensitizes K-Ras mutant pancreatic cancer cells to glutaminase inhibition. Int J Mol Sci. (2021) 22:1870. doi: 10.3390/ijms22041870, PMID: 33672789 PMC7918355

[95] Guo Z Tsai MH Shiao YH Chen LH Wei ML Lv X . DNA (cytosine-5)-methyltransferase 1 as a mediator of mutant p53-determined p16(ink4A) down-regulation. J Biomed Sci. (2008) 15:163–8. doi: 10.1007/s11373-007-9222-y, PMID: 18038118

[96] Rahnamoun H Hong J Sun Z Lee J Lu H Lauberth SM . Mutant p53 regulates enhancer-associated H3K4 monomethylation through interactions with the methyltransferase MLL4. J Biol Chem. (2018) 293:13234–46. doi: 10.1074/jbc.RA118.003387, PMID: 29954944 PMC6109924

[97] Su Z Kon N Yi J Zhao H Zhang W Tang Q . Specific regulation of BACH1 by the hotspot mutant p53R175H reveals a distinct gain-of-function mechanism. Nat Cancer. (2023) 4:564–81. doi: 10.1038/s43018-023-00532-z, PMID: 36973430 PMC10320414

[98] Zhang W Qian W Gu J Gong M Zhang W Zhang S . Mutant p53 driven-LINC00857, a protein scaffold between FOXM1 and deubiquitinase OTUB1, promotes the metastasis of pancreatic cancer. Cancer Lett. (2023) 552:215976. doi: 10.1016/j.canlet.2022.215976, PMID: 36272615

[99] Zhang R Wang X Ying X Huang Y Zhai S Shi M . Hypoxia-induced long non-coding RNA LINC00460 promotes p53 mediated proliferation and metastasis of pancreatic cancer by regulating the miR-4689/UBE2V1 axis and sequestering USP10. Int J Med Sci. (2023) 20:1339–57. doi: 10.7150/ijms.87833, PMID: 37786443 PMC10542025

[100] Liu N Jiang X Zhang G Long S Li J Jiang M . LncRNA CARMN m6A demethylation by ALKBH5 inhibits mutant p53-driven tumour progression through miR-5683/FGF2. Clin Trans Med. (2024) 14:e1777. doi: 10.1002/ctm2.1777, PMID: 39039912 PMC11263751

[101] Di Agostino S Valenti F Sacconi A Fontemaggi G Pallocca M Pulito C . Long non-coding MIR205HG depletes hsa-miR-590-3p leading to unrestrained proliferation in head and neck squamous cell carcinoma. Theranostics. (2018) 8:1850–68. doi: 10.7150/thno.22167, PMID: 29556360 PMC5858504

[102] Madrigal T Ortega-Bernal D Herrera LA González-De la Rosa CH Domínguez-Gómez G Aréchaga-Ocampo E . Mutant p53 gain-of-function induces migration and invasion through overexpression of miR-182-5p in cancer cells. Cells. (2023) 12:2506. doi: 10.3390/cells12202506, PMID: 37887350 PMC10605582

[103] Neilsen P Noll J Mattiske S Bracken CP Gregory P Schulz RB . Mutant p53 drives invasion in breast tumors through up-regulation of miR-155. Oncogene. (2012) 32:2992–3000. doi: 10.1038/onc.2012.305, PMID: 22797073

[104] Garibaldi F Falcone E Trisciuoglio D Colombo T Lisek K Walerych D . Mutant p53 inhibits miRNA biogenesis by interfering with the microprocessor complex. Oncogene. (2016) 35:3760–70. doi: 10.1038/onc.2016.51, PMID: 26996669

[105] Muller PAJ Trinidad AG Caswell PT Norman J Vousden KH . Mutant p53 regulates Dicer through p63-dependent and -independent mechanisms to promote an invasive phenotype. J Biol Chem. (2013) 289:122–32. doi: 10.1074/jbc.M113.502138, PMID: 24220032 PMC3879536

[106] Luo P Wang Q Ye Y Zhang J Lu D Cheng L . MiR-223-3p functions as a tumor suppressor in lung squamous cell carcinoma by miR-223-3p-mutant p53 regulatory feedback loop. J Exp Clin Cancer Res. (2019) 38:74. doi: 10.1186/s13046-019-1079-1, PMID: 30755230 PMC6373043

[107] Wang Z Li Y Yang J Sun Y He Y Wang Y . CircCFL1 promotes TNBC stemness and immunoescape via deacetylation-mediated c-Myc deubiquitylation to facilitate mutant TP53 transcription. Advanced Sci. (2024) 11:e2404628. doi: 10.1002/advs.202404628, PMID: 38981022 PMC11425638

[108] Verduci L Ferraiuolo M Sacconi A Ganci F Vitale J Colombo T . The oncogenic role of circPVT1 in head and neck squamous cell carcinoma is mediated through the mutant p53/YAP/TEAD transcription-competent complex. Genome Biol. (2017) 18:237. doi: 10.1186/s13059-017-1368-y, PMID: 29262850 PMC5738800

[109] Fang L Du WW Lyu J Dong J Zhang C Yang W . Enhanced breast cancer progression by mutant p53 is inhibited by the circular RNA circ-Ccnb1. Cell Death Differentiation. (2018) 25:2195–208. doi: 10.1038/s41418-018-0115-6, PMID: 29795334 PMC6261950

[110] De Leo A Ugolini A Yu X Scirocchi F Scocozza D Peixoto B . Glucose-driven histone lactylation promotes the immunosuppressive activity of monocyte-derived macrophages in glioblastoma. Immunity. (2024) 57:1105–1123.e8. doi: 10.1016/j.immuni.2024.04.008, PMID: 38703775 PMC11114377

[111] Wang R Li C Cheng Z Li M Shi J Zhang Z . H3K9 lactylation in Malignant cells facilitates CD8^+^ T cell dysfunction and poor immunotherapy response. Cell Rep. (2024) 43:114686. doi: 10.1016/j.celrep.2024.114686, PMID: 39216002

[112] Xue Q Peng W Zhang S Wei X Ye L Wang Z . Lactylation-driven TNFR2 expression in regulatory T cells promotes the progression of Malignant pleural effusion. J ImmunoTherapy Cancer. (2024) 12:e010040. doi: 10.1136/jitc-2024-010040, PMID: 39721754 PMC11683941

[113] Huang ZW Zhang XN Zhang L Liu LL Zhang JW Sun YX . STAT5 promotes PD-L1 expression by facilitating histone lactylation to drive immunosuppression in acute myeloid leukemia. Signal Transduction Targeted Ther. (2023) 8:391. doi: 10.1038/s41392-023-01637-8, PMID: 37777506 PMC10542808

[114] Sun J Feng Q He Y Wang M Wu Y . Lactate activates CCL18 expression via H3K18 lactylation in macrophages to promote tumorigenesis of ovarian cancer. Acta Biochim Biophys Sin. (2024) 56:1373–86. doi: 10.3724/abbs.2024088, PMID: 39010846 PMC11543520

[115] Wang S Huang T Wu Q Yuan H Wu X Yuan F . Lactate reprograms glioblastoma immunity through CBX3-regulated histone lactylation. J Clin Invest. (2024) 134:e176851. doi: 10.1172/JCI176851, PMID: 39545414 PMC11563687

[116] Hou X Hong Z Zeng H Zhang C Zhang P Ma D . Lactylation in cancer biology: Unlocking new avenues for research and therapy. Cancer Commun. (2025) 45:1367–1406. doi: 10.1002/cac2.70054, PMID: 40831053 PMC12629867

[117] Hasan MN Capuk O Patel SM Sun D . The role of metabolic plasticity of tumor-associated macrophages in shaping the tumor microenvironment immunity. Cancers. (2022) 14:3331. doi: 10.3390/cancers14143331, PMID: 35884391 PMC9316955

[118] Tahiliani M Koh KP Shen Y Pastor WA Bandukwala H Brudno Y . Conversion of 5-methylcytosine to 5-hydroxymethylcytosine in mammalian DNA by MLL partner TET1. Science. (2009) 324:930–5. doi: 10.1126/science.1170116, PMID: 19372391 PMC2715015

[119] Chen Z Zang J Whetstine J Hong X Davrazou F Kutateladze TG . Structural insights into histone demethylation by JMJD2 family members. Cell. (2006) 125:691–702. doi: 10.1016/j.cell.2006.04.024, PMID: 16677698

[120] Li L Zeng X Chao Z Luo J Guan W Zhang Q . Targeting alpha-ketoglutarate disruption overcomes immunoevasion and improves PD-1 blockade immunotherapy in renal cell carcinoma. Advanced Sci. (2023) 10:e2301975. doi: 10.1002/advs.202301975, PMID: 37526345 PMC10520657

[121] Liu PS Wang H Li X Chao T Teav T Christen S . α-ketoglutarate orchestrates macrophage activation through metabolic and epigenetic reprogramming. Nat Immunol. (2017) 18:985–94. doi: 10.1038/ni.3796, PMID: 28714978

[122] Klysz D Tai X Robert PA Craveiro M Cretenet G Oburoglu L . Glutamine-dependent α-ketoglutarate production regulates the balance between T helper 1 cell and regulatory T cell generation. Sci Signaling. (2015) 8:ra97. doi: 10.1126/scisignal.aab2610, PMID: 26420908

[123] Matias MI Yong CS Foroushani A Goldsmith C Mongellaz C Sezgin E . Regulatory T cell differentiation is controlled by αKG-induced alterations in mitochondrial metabolism and lipid homeostasis. Cell Rep. (2021) 37:109911. doi: 10.1016/j.celrep.2021.109911, PMID: 34731632 PMC10167917

[124] López-Moyado IF Ko M Hogan PG Rao A . TET enzymes in the immune system: From DNA demethylation to immunotherapy, inflammation, and cancer. Annu Rev Immunol. (2024) 42:455–88. doi: 10.1146/annurev-immunol-080223-044610, PMID: 38360546

[125] Wellen KE Hatzivassiliou G Sachdeva UM Bui TV Cross J Thompson CB . ATP-citrate lyase links cellular metabolism to histone acetylation. Science. (2009) 324:1076–80. doi: 10.1126/science.1164097, PMID: 19461003 PMC2746744

[126] Gao X Lin SH Ren F Li JT Chen JJ Yao CB . Acetate functions as an epigenetic metabolite to promote lipid synthesis under hypoxia. Nat Commun. (2016) 7:11960. doi: 10.1038/ncomms11960, PMID: 27357947 PMC4931325

[127] Wang H Yi X Wang X Yang Y Zhang H Wang H . Nucleo-cytosolic acetyl-CoA drives tumor immune evasion by regulating PD-L1 in melanoma. Cell Rep. (2024) 43:115015. doi: 10.1016/j.celrep.2024.115015, PMID: 39602308

[128] Gautam J Wu J Lally JSV McNicol JD Fayyazi R Ahmadi E . ACLY inhibition promotes tumour immunity and suppresses liver cancer in MASH-HCC models. Nature. (2025) 645:507–17. doi: 10.1038/s41586-025-09297-0, PMID: 40739358 PMC12422966

[129] Wang J Yang Y Shao F Meng Y Guo D He J . Acetate reprogrammes tumour metabolism and promotes PD-L1 expression and immune evasion by upregulating c-Myc. Nat Metab. (2024) 6:914–32. doi: 10.1038/s42255-024-01037-4, PMID: 38702440

[130] Dang Q Wang T Wang Y Ye Z Li B Pan X . ACSS2/AATF drives soluble FasL-mediated CD8^+^ T cell apoptosis in pancreatic neuroendocrine tumors. Advanced Sci. (2025) 12:e06883. doi: 10.1002/advs.202506883, PMID: 40791180 PMC12561415

[131] Deng S Wang M Wang C Zeng Y Qin X Tan Y . p53 downregulates PD-L1 expression via miR-34a to inhibit the growth of triple-negative breast cancer cells: A potential clinical immunotherapeutic target. Mol Biol Rep. (2023) 50:577–87. doi: 10.1007/s11033-022-08047-z, PMID: 36352176

[132] Hassin O Nataraj NB Shreberk-Shaked M Aylon Y Yaeger R Fontemaggi G . Different hotspot p53 mutants (R175H vs R273H) exert distinct oncogenic phenotypes with differential transcription programs. bioRxiv. (2022) 13:2800. doi: 10.1101/2022.03.15.484392 PMC9120190 35589715

[133] Tseng YH Tran TTM Tsai Chang J Huang YT Nguyen AT Chang IY . Utilizing TP53 hotspot mutations as effective predictors of gemcitabine treatment outcome in non-small-cell lung cancer. Cell Death Discov. (2025) 11:26. doi: 10.1038/s41420-025-02300-7, PMID: 39870629 PMC11772833

[134] Zeng HH Yang Z Qiu YB Bashir S Li Y Xu M . Detection of a novel panel of 24 genes with high frequencies of mutation in gastric cancer based on next-generation sequencing. World J Clin Cases. (2022) 10:4761–4775. doi: 10.12998/wjcc.v10.i15.4761, PMID: 35801059 PMC9198883

[135] Cortez MA Ivan C Valdecanas D Wang X Peltier HJ Ye Y . PDL1 regulation by p53 via miR-34. J Natl Cancer Institute. (2015) 108:djv303. doi: 10.1093/jnci/djv303, PMID: 26577528 PMC4862407

[136] Yu J Ling S Hong J Zhang L Zhou W Yin L . TP53/mTORC1-mediated bidirectional regulation of PD-L1 modulates immune evasion in hepatocellular carcinoma. J ImmunoTherapy Cancer. (2023) 11:e007479. doi: 10.1136/jitc-2023-007479, PMID: 38030304 PMC10689408

[137] Cheng M Shi Y Kong L . Expression and correlation analysis of p53, PD-1, and PD-L1 in breast cancer. J Clin Exp Pathol. (2018) 34:1307–10. doi: 10.13315/j.cnki.cjcep.2018.12.003

[138] George J Maas L Abedpour N Cartolano M Kaiser L Fischer RN . Evolutionary trajectories of small cell lung cancer under therapy. Nature. (2024) 627:880–9. doi: 10.1038/s41586-024-07177-7, PMID: 38480884 PMC10972747

[139] S A Chakraborty A Patnaik S . Clonal evolution and expansion associated with therapy resistance and relapse of colorectal cancer. Mutat Res Rev Mutat Res. (2022) 790:108445. doi: 10.1016/j.mrrev.2022.108445, PMID: 36371022

[140] Färkkilä A Rodríguez A Oikkonen J Gulhan DC Nguyen H Domínguez J . Heterogeneity and clonal evolution of acquired PARP inhibitor resistance in TP53- and BRCA1-deficient cells. Cancer Res. (2021) 81:2774–87. doi: 10.1158/0008-5472.CAN-20-2912, PMID: 33514515 PMC8323804

[141] Hoell JI Ginzel S Kuhlen M Kloetgen A Gombert M Fischer U . Pediatric ALL relapses after allo-SCT show high individuality, clonal dynamics, selective pressure, and druggable targets. Blood Adv. (2019) 3:3143–56. doi: 10.1182/bloodadvances.2019000051, PMID: 31648313 PMC6849953

[142] Malcikova J Pavlova S Kunt Vonkova B Radova L Plevova K Kotaskova J . Low-burden TP53 mutations in CLL: Clinical impact and clonal evolution within the context of different treatment options. Blood. (2021) 138:2670–85. doi: 10.1182/blood.2020009530, PMID: 33945616 PMC8703362

[143] Haertle L Munawar U Hernández HNC Arroyo-Barea A Heckel T Cuenca I . Clonal competition assays identify fitness signatures in cancer progression and resistance in multiple myeloma. Hemasphere. (2024) 8:e110. doi: 10.1002/hem3.110, PMID: 38993727 PMC11237348

[144] Cowzer D Darmofal M Seier K Thummalapalli R Walch H El Dika I . Clinical utility and prognostic implications of circulating cell-free DNA in biliary tract cancer. JCO Precis Oncol. (2025) 9:e2500355. doi: 10.1200/PO-25-00355, PMID: 40956997 PMC12443323

[145] Moding EJ Liu Y Nabet BY Chabon JJ Chaudhuri AA Hui AB . Circulating tumor DNA dynamics predict benefit from consolidation immunotherapy in locally advanced non-small cell lung cancer. Nat Cancer. (2020) 1:176–83. doi: 10.1038/s43018-019-0011-0, PMID: 34505064 PMC8425388

[146] Stasik S Mende M Schuster C Mahler S Aust D Tannapfel A . Sensitive quantification of cell-free tumor DNA for early detection of recurrence in colorectal cancer. Front Genet. (2022) 12:811291. doi: 10.3389/fgene.2021.811291, PMID: 35069704 PMC8766716

[147] Deveson IW Gong B Lai K LoCoco JS Richmond TA Schageman J . Evaluating the analytical validity of circulating tumor DNA sequencing assays for precision oncology. Nat Biotechnol. (2021) 39:1115–28. doi: 10.1038/s41587-021-00857-z, PMID: 33846644 PMC8434938

[148] Zhu L Xu R Yang L Shi W Zhang Y Liu J . Minimal residual disease (MRD) detection in solid tumors using circulating tumor DNA: a systematic review. Frontiers in Genetics. (2023) 14:1172108. doi: 10.3389/fgene.2023.1172108, PMID: 37636270 PMC10448395

[149] Gundem G Van Loo P Kremeyer B Alexandrov LB Tubio JMC Papaemmanuil E . The evolutionary history of lethal metastatic prostate cancer. Nature. (2015) 520:353–7. doi: 10.1038/nature14347, PMID: 25830880 PMC4413032

[150] Zhang Q Bykov VJN Wiman KG Zawacka-Pankau J . APR-246 reactivates mutant p53 by targeting cysteines 124 and 277. Cell Death Dis. (2018) 9:439. doi: 10.1038/s41419-018-0463-7, PMID: 29670092 PMC5906465

[151] Ceder S Eriksson SE Cheteh EH Dawar S Corrales Benitez M Bykov VJN . A thiol-bound drug reservoir enhances APR-246-induced mutant p53 tumor cell death. EMBO Mol Med. (2021) 13:e10852. doi: 10.15252/emmm.201910852, PMID: 33314700 PMC7863383

[152] U.S. National library of medicine. Bethesda, MD, USA: ClinicalTrials.gov (2022). Available online at: https://clinicaltrials.gov/study/NCT03072043 (Accessed October 8, 2025).

[153] U.S. National library of medicine. Bethesda, MD, USA: ClinicalTrials.gov (2020). Available online at: https://clinicaltrials.gov/study/NCT03588078 (Accessed October 8, 2025).

[154] U.S. National library of medicine. Bethesda, MD, USA: ClinicalTrials.gov (2023). Available online at: https://clinicaltrials.gov/study/NCT04419389 (Accessed October 8, 2025).

[155] U.S. National library of medicine. Bethesda, MD, USA: ClinicalTrials.gov (2022). Available online at: https://clinicaltrials.gov/study/NCT03745716 (Accessed October 8, 2025).

[156] U.S. National library of medicine. Bethesda, MD, USA: ClinicalTrials.gov (2022). Available online at: https://clinicaltrials.gov/study/NCT04383938 (Accessed October 8, 2025).

[157] Puzio-Kuter AM Xu L McBrayer MK Dominique R Li HH Fahr BJ . Restoration of the tumor suppressor function of Y220C-mutant p53 by Rezatapopt, a small-molecule reactivator. Cancer Discov. (2025) 15:1159–79. doi: 10.1158/2159-8290.CD-24-1421, PMID: 39945593 PMC12130801

[158] Glimmers of hope for targeting p53. Cancer Discov. (2022) 12:OF5. doi: 10.1158/2159-8290.CD-ND2022-0009, PMID: 35686841

[159] Tang Y Song H Wang Z Xiao S Xiang X Zhan H . Repurposing antiparasitic antimonials to noncovalently rescue temperature-sensitive p53 mutations. Cell Rep. (2022) 39:110622. doi: 10.1016/j.celrep.2022.110622, PMID: 35417717

[160] U.S. National library of medicine. Bethesda, MD, USA: ClinicalTrials.gov (2021). Available online at: https://clinicaltrials.gov/study/NCT04906031 (Accessed October 8, 2025).

[161] Li D Marchenko ND Moll UM . SAHA shows preferential cytotoxicity in mutant p53 cancer cells by destabilizing mutant p53 through inhibition of the HDAC6-Hsp90 chaperone axis. Cell Death Differentiation. (2011) 18:1904–13. doi: 10.1038/cdd.2011.71, PMID: 21637290 PMC3170683

[162] U.S. National library of medicine. Bethesda, MD, USA: ClinicalTrials.gov (2017). Available online at: https://clinicaltrials.gov/study/NCT01339871 (Accessed October 8, 2025).

[163] Pereira M Matuszewska K Glogova A Petrik J . Mutant p53, the mevalonate pathway and the tumor microenvironment regulate tumor response to statin therapy. Cancers. (2022) 14:3500. doi: 10.3390/cancers14143500, PMID: 35884561 PMC9323637

[164] Chou CW Lin CH Hsiao TH Lo CC Hsieh CY Huang CC . Therapeutic effects of statins against lung adenocarcinoma via p53 mutant-mediated apoptosis. Sci Rep. (2019) 9:20403. doi: 10.1038/s41598-019-56532-6, PMID: 31892709 PMC6938497

[165] Wang G Peng T Chen L Xiong K Ju L Qian K . Mevalonate pathway inhibition reduces bladder cancer metastasis by modulating RhoB protein stability and integrin β1 localization. Commun Biol. (2024) 7:1476. doi: 10.1038/s42003-024-07067-8, PMID: 39521858 PMC11550803

[166] Sethunath V Hu H De Angelis C Veeraraghavan J Qin L Wang N . Targeting the mevalonate pathway to overcome acquired anti-HER2 treatment resistance in breast cancer. Mol Cancer Res. (2019) 17:2318–30. doi: 10.1158/1541-7786.MCR-19-0756, PMID: 31420371 PMC6825570

[167] U.S. National library of medicine. Bethesda, MD, USA: ClinicalTrials.gov (2023). Available online at: https://clinicaltrials.gov/study/NCT04767984 (Accessed October 8, 2025).

[168] U.S. National library of medicine. Bethesda, MD, USA: ClinicalTrials.gov (2022). Available online at: https://clinicaltrials.gov/study/NCT03560882 (Accessed October 8, 2025).

[169] U.S. National library of medicine. Bethesda, MD, USA: ClinicalTrials.gov (2023). Available online at: https://clinicaltrials.gov/study/NCT03358017 (Accessed October 8, 2025).

[170] Palomar-Siles M Heldin A Zhang M Strandgren C Yurevych V van Dinter JT . Translational readthrough of nonsense mutant TP53 by mRNA incorporation of 5-fluorouridine. Cell Death Dis. (2022) 13:997. doi: 10.1038/s41419-022-05431-2, PMID: 36433934 PMC9700717

[171] U.S. National library of medicine. Bethesda, MD, USA: ClinicalTrials.gov (2022). Available online at: https://clinicaltrials.gov/study/NCT02340117 (Accessed October 8, 2025).

[172] U.S. National library of medicine. Bethesda, MD, USA: ClinicalTrials.gov (2022). Available online at: https://clinicaltrials.gov/study/NCT03554707 (Accessed October 8, 2025).

[173] Seligmann JF Fisher DJ Brown LC Adams RA Graham J Quirke P . Inhibition of WEE1 is effective in TP53- and RAS-mutant metastatic colorectal cancer: A randomized trial (FOCUS4-C) comparing adavosertib (AZD1775) with active monitoring. J Clin Oncol. (2021) 39:3705–15. doi: 10.1200/JCO.21.01435, PMID: 34538072 PMC8601321

[174] Oza AM Estevez-Diz M Grischke EM Hall M Marmé F Provencher D . A biomarker-enriched, randomized phase II trial of adavosertib (AZD1775) plus paclitaxel and carboplatin for women with platinum-sensitive TP53-mutant ovarian cancer. Clin Cancer Res. (2020) 26:4767–76. doi: 10.1158/1078-0432.CCR-20-0219, PMID: 32611648

[175] Hardwick NR Carroll M Kaltcheva T Qian D Lim D Leong L . p53MVA therapy in patients with refractory gastrointestinal Malignancies elevates p53-specific CD8^+^ T-cell responses. Clin Cancer Res. (2014) 20:4459–70. doi: 10.1158/1078-0432.CCR-13-3361, PMID: 24987057 PMC4155000

[176] U.S. National library of medicine. Bethesda, MD, USA: ClinicalTrials.gov (2023). Available online at: https://clinicaltrials.gov/study/NCT03897881 (Accessed October 8, 2025).

[177] Miller ZA Muthuswami S Mueller A Ma RZ Sywanycz SM Naik A . GLUT1 inhibitor BAY-876 induces apoptosis and enhances anti-cancer effects of bitter receptor agonists in head and neck squamous carcinoma cells. Cell Death Discov. (2024) 10:339. doi: 10.1038/s41420-024-02106-z, PMID: 39060287 PMC11282258

[178] U.S. National library of medicine. Bethesda, MD, USA: ClinicalTrials.gov (2022). Available online at: https://clinicaltrials.gov/study/NCT01791595 (Accessed October 8, 2025).

[179] U.S. National library of medicine. Bethesda, MD, USA: ClinicalTrials.gov (2024). Available online at: https://clinicaltrials.gov/study/NCT05681709 (Accessed October 8, 2025).

